# Role of ROS and RNS Sources in Physiological and Pathological Conditions

**DOI:** 10.1155/2016/1245049

**Published:** 2016-07-12

**Authors:** Sergio Di Meo, Tanea T. Reed, Paola Venditti, Victor Manuel Victor

**Affiliations:** ^1^Dipartimento di Biologia, Università di Napoli “Federico II”, 80126 Napoli, Italy; ^2^Department of Chemistry, Eastern Kentucky University, Richmond, KY 40475, USA; ^3^Service of Endocrinology, University Hospital Dr. Peset, Foundation for the Promotion of Health and Biomedical Research in the Valencian Region (FISABIO), 46010 Valencia, Spain

## Abstract

There is significant evidence that, in living systems, free radicals and other reactive oxygen and nitrogen species play a double role, because they can cause oxidative damage and tissue dysfunction and serve as molecular signals activating stress responses that are beneficial to the organism. Mitochondria have been thought to both play a major role in tissue oxidative damage and dysfunction and provide protection against excessive tissue dysfunction through several mechanisms, including stimulation of opening of permeability transition pores. Until recently, the functional significance of ROS sources different from mitochondria has received lesser attention. However, the most recent data, besides confirming the mitochondrial role in tissue oxidative stress and protection, show interplay between mitochondria and other ROS cellular sources, so that activation of one can lead to activation of other sources. Thus, it is currently accepted that in various conditions all cellular sources of ROS provide significant contribution to processes that oxidatively damage tissues and assure their survival, through mechanisms such as autophagy and apoptosis.

## 1. Introduction

The existence of free radicals, known in chemistry since the beginning of the 20th century [1], was discovered in biological systems in 1954 [2]. In the same year, Gerschman et al. proposed that the noxious effects of hyperbaric oxygen and X irradiation had a common mechanism which involved radical and nonradical species, resulting from partial reduction of oxygen [3]. Soon after, Harman suggested that these species, at present referred to as reactive oxygen species (ROS), might play a role in the aging process [4].

Progress in the free radical knowledge occurred in 1969 when the enzyme superoxide dismutase (SOD) was isolated [5]. The SOD discovery inspired a large number of studies, which contributed to the knowledge of the ROS, even though for several decades they were thought to cause exclusively damaging effects. This view was mainly supported by the finding that ROS readily react with most biological macromolecules, causing their oxidative modification, ultimately resulting in the loss of their function [6]. Actually, ROS include species, such as the hydroxyl radical (^•^OH), whose reactivity is so high that it reacts very close to its site of formation [7], and other species, such as superoxide (O_2_^•−^) and hydrogen peroxide (H_2_O_2_), which are less reactive (Table 1). Thus, nitrogen containing species, which are now indicated as reactive nitrogen species (RNS), include nitric oxide (NO^•^), which is relatively unreactive, and its derivative the peroxynitrite (ONOO^−^), a powerful oxidant, able to damage many biological molecules [8].

Nonetheless, ROS were considered as one of the key players in tissue injury, if occurring in organisms with a system of biochemical defenses to neutralize the oxidative effects of ROS [9], but the balance between ROS generation and antioxidant system activity is slightly tipped in favor of the ROS so that, in living systems, there is a continuous low level of oxidative damage. Moreover, when a greater imbalance occurs in favor of the ROS, oxidative stress ensues [10]. Oxidative stress is a phenomenon which was related to the development of many pathological conditions. Pathologies where ROS were identified as causal factors include cardiovascular disease, diabetes, rheumatoid arthritis, cancer, and neurodegenerative disorders [11] and the use of exogenous antioxidants was proposed for their treatment [12].

The finding that ^•^OH radical stimulated formation of cyclic guanosine monophosphate (cGMP) [13] led to an opposing view about the ROS role in biological systems. Since then, it became clear that living systems not only adapted to a coexistence with free radicals but also developed methods to turn these toxicants to their own advantage utilizing them in critical physiological processes. This view has been supported by the observation that, at the cellular level, ROS regulate growth, apoptosis, and other signaling, while, at the system level, they contribute to complex functions, including blood pressure regulation, cognitive function, and immune function [14]. It has also been shown that whereas accumulation of oxidative damage results in organism death [15], several longevity-promoting interventions increase generation of ROS that activate stress responses that are beneficial to the organism and extend lifespan [16].

Like ROS, RNS play a dual role since they can be either harmful or beneficial to living systems. Nitric oxide, early identified as a signaling molecule in blood vessel modulation [17] and now known as a regulator of important physiological processes [18], can mediate cellular toxicity damaging metabolic enzymes and generating, by reaction with superoxide, peroxynitrite [19].

Although the role of ROS and RNS in cellular damage and signal transduction is well-established, several controversial questions remain open. At low concentrations, ROS and RNS play an important role as regulatory mediators in signaling processes, whereas, at moderate or high concentrations, they are harmful for living organisms inactivating important cellular molecules. This suggests that the concentrations of reactive species determine the shift from their advantageous to detrimental effects, but the concentrations to which this shift happens are not generally known. Moreover, given the wide range of possible targets and the general reactivity of cellular oxidants, it is unclear how any specificity in their opposite actions can be achieved. It has been suggested that contributing factors may include cell type, duration of oxidant production, reactive species produced, and the localization of their source and their targets [20], but our information about such a matter is still scarce and discordant. For example, it has often been assumed that mitochondria are the main cellular source of ROS in physiological and pathological conditions [21], so that these organelles have been thought to play a crucial role in several human diseases and aging [22]. However, in the light of available data it seems that evidence that mitochondria are the main source of cellular ROS is lacking [23]. The issue is complicated by the existence of a strong interaction among the various sources of ROS generation in the cell [24, 25], which makes it difficult to establish what source of reactive species plays a main role in different physiological and pathological conditions.

To contribute to the understanding of the role of reactive species sources in health and disease, the present review, after examining the cellular localization and supposed involvement of such sources in tissue dysfunction and protection, examines experimental evidence concerning their harmful and protective effects on a normal physiological activity, such as exercise, and on pathologic conditions, such as diabetes and neurodegenerative diseases.

## 2. Cellular ROS and RNS Sources

**Figure 1:**
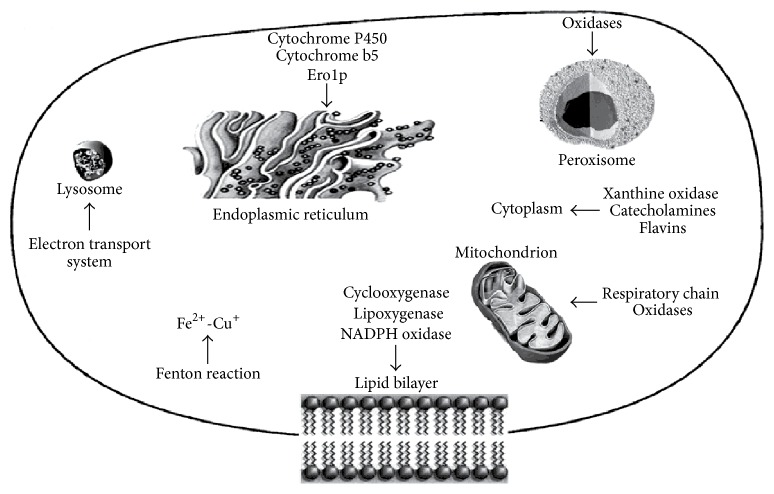
Cellular sources of ROS production. Subcellular organelles and structural and soluble cell components all contribute to production of a wide variety of reactive species (modified from Venditti et al. [116], with permission).

In the living organisms, ROS are generated in several cellular systems localized on the plasma membrane, in the cytosol, in the peroxisomes, and on membranes of mitochondria and endoplasmic reticulum (Figure 1).

### 2.1. ROS Production in the Cytosol

Several soluble cell components, including thiols, hydroquinones, catecholamines, and flavins, can contribute to intracellular ROS production as they are able to undergo redox reactions [26]. Moreover, several cytosolic enzymes produce ROS during their catalytic activity. Probably the most studied ROS producing enzyme is xanthine oxidase (XO). In healthy tissues, the enzyme catalyzing oxidation of hypoxanthine to xanthine and xanthine to uric acid is xanthine dehydrogenase (XDH), which uses NADP^+^ as an electron acceptor. Conversely, in damaged tissues, by either reversible oxidation of cysteine residues or irreversible Ca^2+^-stimulated proteolysis, the enzyme is converted from the dehydrogenase form into the oxidase form, which transfers electrons to molecular oxygen producing the superoxide radical during xanthine or hypoxanthine oxidation [27].

### 2.2. ROS Production by Mitochondria

In aerobic cells, mitochondria are necessary for numerous fundamental functions, including respiration and oxidative energy production, regulation of the intracellular calcium concentration, and control of the fatty acid *β*-oxidation.

For a long time, mitochondria have only been considered for their role in energy production. They utilize about 95% of the oxygen assumed from aerobic animals to obtain energy by oxidizing substances contained in food by transfer of electrons to electron carriers such as NAD^+^, FMN, and FAD. The reduced forms of these molecules, in turn, transfer electrons to the components of the respiratory chain and finally to oxygen in a process which happens in a series of subsequent steps, so that the energy, resulting by the fall in the electron potential energy, is gradually released. In the process, several redox centers, in great part organized in four protein complexes inserted in the inner mitochondrial membrane, are involved [28]. Complexes I and II transfer electrons to the lipid-soluble carrier ubiquinone. From this, the electrons pass through Complex III, cytochrome c (another mobile carrier), and Complex IV, to the oxygen. The fall in electron potential energy is used to pump protons from the mitochondrial matrix to the intermembrane space, thus setting up a proton-motive force [29]. The process by which such a force drives protons back into the matrix through the mitochondrial ATP synthase leading to ATP synthesis is named oxidative phosphorylation [30].

#### 2.2.1. Respiratory Chain

It has now been about 50 years since mitochondrial H_2_O_2_ production in the presence of respiratory substrates was first recorded [31], followed shortly after by the detection of mitochondrial generation of superoxide radical anion [32]. The discovery that electron-transfer along the inner mitochondrial membrane carriers is associated with formation of ROS suggested the mitochondrial involvement in degenerative processes linked to several diseases and aging.

The primary ROS generated within mitochondria by univalent autooxidation of electron carriers is O_2_^•−^ [33], which is converted by mitochondrial SOD into H_2_O_2_, which can be turned into ^•^OH radical via the Fenton reaction:

(1) H2O2+Fe2+⟶OH•+OH−+Fe3+

The main sites involved in mitochondrial ROS production are localized at Complexes I and III [34]. However, succinate dependent ROS production by Complex II from rat skeletal muscle [34] and glycerol 3-phosphate dependent production by Complex II from several rat tissues [35] have also been reported.

To date, the relative importance of each mitochondrial site to ROS production is still controversial, partly due to utilization of different assays, substrates [36], and sources of mitochondria [37]. On the other hand, the localization of the generators is important for establishing ROS effects since it determines if O_2_^•−^ is produced in the mitochondrial matrix or in the intermembrane space. Thus, both generators of Complex I and Complex III release O_2_^•−^ into the matrix where it can damage mitochondrial DNA, whereas Complex III generator also releases O_2_^•−^ into the intermembrane space, where it has easier access to the cytosol [36]. In their classic work, Boveris et al. [38] reported that H_2_O_2_ production by isolated liver mitochondria was about 1-2% of the total oxygen consumption during State 4 respiration and decreased during State 3 respiration. However, lower values, around 0.1–0.2% of the total oxygen consumption, have subsequently been found [39].

#### 2.2.2. Other Mitochondrial Sites of ROS Production

Experiments with isolated enzymes or mitochondria have shown ROS production by several oxidoreductases located in mitochondrial membranes [40], whose contribution to mitochondrial ROS production is, however, unknown. They include monoamine oxidases, which produce H_2_O_2_ at rates that, in brain mitochondria, may be higher than those of other mitochondrial sources [41]; dihydroorotate dehydrogenase, which* in vitro*, in the absence of coenzyme Q, its natural electron acceptor, can produce H_2_O_2_ [42]; *α*-glycerophosphate dehydrogenase, which has been reported to produce H_2_O_2_ in mouse [37] and rat [34] mitochondria oxidizing glycerol-3-phosphate; succinate dehydrogenase, which produces ROS when isolated and incorporated in liposomes in the absence of coenzyme Q [43]; and *α*-ketoglutarate dehydrogenase complex, which has been found to generate both O_2_^•−^ and H_2_O_2_ in isolated mouse brain mitochondria [44].

### 2.3. ROS Production by Peroxisomes

Although peroxisomes have long been known as organelles involved in cellular metabolism of H_2_O_2_ [45], it is now clear that they are involved in several metabolic pathways [46]. Important functions performed by peroxisomes include fatty acid *β*- and *α*-oxidation, amino acid and glyoxylate metabolism, and synthesis of lipidic compounds [47], and most enzymes catalyzing these processes produce ROS during their activity [48].

Early studies indicated that about 35% of the peroxide formed in rat liver is derived from peroxisomal oxidases [38]. Moreover, the observation that about 20–60% of the H_2_O_2_ generated inside peroxisomes diffused to the surrounding medium demonstrated that H_2_O_2_ can readily cross the peroxisomal membrane. The peroxide diffuses through the Pxmp2 channel permeable to small solutes [49], even though H_2_O_2_ generated by urate oxidase localized in the peroxisome core can be directly released in the cytosol through crystalloid core tubules [50]. In any case, it is apparent that, despite high content of catalase (CAT), peroxisomes are unable to prevent the H_2_O_2_ release.

Peroxisomes also contain xanthine oxidase [51] and the inducible form of nitric oxide synthase (see below) [52], which produce O_2_^•−^ and NO^•^, respectively. Because such species react rapidly forming ONOO and H_2_O_2_ gives rise to ^•^OH radicals via Fenton reaction, peroxisomes are a potential source of such highly reactive species. Because, if uncontrolled, ROS and RNS are very damaging, peroxisomes, in addition to catalase, contain other antioxidant enzymes [50]. However, in the light of their capacity to produce membrane permeant reactive species, such as H_2_O_2_ and NO^•^, it is highly likely that, under some physiological or pathological conditions, peroxisomes may act as a source of H_2_O_2_ and NO^•^ in living cells [50].

### 2.4. ROS Production by Endoplasmic Reticulum

The endoplasmic reticulum (ER) is involved in multiple functions, such as synthesis, folding, and transport of Golgi, lysosomal, secretory, and cell-surface proteins [53], calcium storage [54], lipid metabolism, and, in some cell types, drug detoxification [55].

Smooth endoplasmic reticulum presents a chain of electron transport, constituted by two systems devoted to xenobiotic metabolism and introduction of double bonds in fatty acids, which are also able to produce ROS. Another microsomal system, which shares this ability, provides oxidative protein folding.

#### 2.4.1. Xenobiotic Metabolism

The metabolism of xenobiotics generally occurs in two phases. Phase I reactions introduce a polar group in a lipophilic substrate (AH) using O_2_ and a reducing agent (RH_2_):

(2) $$\begin{array}{c} AH+O_{2}+{RH}_{2}⟶AOH+R+H_{2}O \end{array}$$

In the reaction, known as a monooxygenase reaction, it is involved in a system constituted by a flavoprotein (*NADPH*-cytochrome P450 reductase) and cytochromes known collectively as cytochromes P450 (CYPs).

The Phase II reactions are conjugation reactions in which an endogenous molecule is added to the Phase I reaction product or sometimes directly to the xenobiotic.

In whole, the process detoxifies xenobiotics converting them into species that are more water soluble and easier to excrete in urine or can be conjugated with substances which make their urinary or biliary excretion further easier.

The membranes of the endoplasmic reticulum system were recognized as a source of H_2_O_2_ in 1957 by Gillette et al. [56] that assumed that the NADPH-cytochrome c reductase might be the microsomal H_2_O_2_ generator. However, it was subsequently reported that O_2_^•−^ [57] and H_2_O_2_ [58] could be formed by decay of two intermediates of the catalytic cycle.

The microsomal cytochrome P450-dependent monooxygenase system is one of the major producers of ROS in the liver cell [59]. Indeed, estimates performed using NADPH as substrate indicated that microsomes contribute to H_2_O_2_ production in rat liver by 45% [38].

#### 2.4.2. Unsaturated Fatty Acid Production

In most organisms, unsaturated fatty acids are produced by desaturases, which convert a single bond between two carbon atoms in a fatty acyl chain into a double bond [60]. In the ER system, which allows for fatty acid desaturation, cytochrome b5 acts as an electron-transfer component, with two possible modes of action. First, desaturation can be carried out by a multienzyme system, composed of desaturase, NADH cytochrome b5 reductase, and cytochrome b5. In the reaction, cytochrome b5 transfers electrons by lateral diffusion, from NADH cytochrome b5 reductase to the desaturase which introduces carbon-carbon double bonds into fatty acids using one molecule of O_2_ and forming two molecules of H_2_O [61]. Secondly, many desaturases are modular proteins that are composed of desaturase and cytochrome b5 modules [62]. The fusion of the desaturase and cytochrome b5 domains makes the NADH cytochrome b5 reductase able to directly transfer electrons to the catalytic site of the cytochrome b5 fusion desaturases via the cytochrome b5-like domain without the requirement of an independent cytochrome b5 [63]. This can increase the rate of electron-transfer by presenting a correctly oriented heme group with respect to the dioxo-iron cluster, eliminating the need for diffusion and reorientation of the reduced cytochrome b5 [63].

The two electron transport systems do not act independently of each other and cytochrome b5 may play a role in the NADPH-dependent oxidation of xenobiotics. Indeed, cytochrome b5 exhibits a positive action on cytochrome P450 monooxygenase reaction, which is due to the transfer of the second of the two electrons necessary for molecular oxygen activation to cytochrome P450 [64]. This transfer makes the catalysis faster and reduces the time for formation of side products, such as H_2_O_2_ and O_2_^•−^ [64]. Recently, it has also been demonstrated that NADH cytochrome b5 reductase can leak electrons to O_2_ to make O_2_^•−^ and this can be an additional source of O_2_^•−^ *in vivo* [65].

#### 2.4.3. Protein Folding

Most proteins synthesized in ER are stabilized by formation of intramolecular disulfide bonds, process that requires oxidation of free sulphydryl groups. The feasibility of protein oxidative folding in intraluminal ER milieu is ensured by an oxidized (GSSG) to reduced (GSH) glutathione ratio higher than that within the cytosol [66]. The formation of disulfide bonds in proteins is driven by protein disulfide isomerase (PDI), a member of the thioredoxin family, and endoplasmic reticulum-resident protein (Ero1p), which is tightly associated with FAD moiety [66]. Ero1p functions as an oxidase for PDI: oxidizing equivalents flow from Ero1p to substrate proteins via PDI, through direct dithiol-disulfide exchange between PDI and Ero1p [67].

The process of oxidative protein folding uses molecular oxygen as the source of oxidizing equivalents. Indeed, Ero1p transfers electrons from PDI to molecular oxygen by a FAD-dependent reaction, resulting in ER protein folding-induced oxidative stress [68]. By theoretical calculation, it was estimated that protein thiol oxidation via PDI and Ero1 could account for up to 25% of cellular ROS produced during protein synthesis [69].

### 2.5. ROS Production by Plasma Membrane

The plasma membrane is involved in several cellular processes such as cell adhesion, ion conductivity, and cell signaling. It is also a key site of free radical reactions because it is generally exposed to an oxidizing environment. ROS, which in tissues could be generated from dysfunctional cells [70], cause oxidative damage of membrane components unless efficient antioxidant systems are operative. The increase in membrane permeability, caused by oxidation of lipids or structurally important proteins, can result in a decrease in transmembrane ion gradients, loss of secretory functions, and inhibition of cellular metabolic processes.

Free radicals can be produced during the conversion of arachidonic acid into products, such as prostaglandins, thromboxanes, and leukotrienes, by membrane associated enzymes such as lipoxygenase and cyclooxygenase [71]. Such enzymes metabolize arachidonic acid released from membrane phospholipids via phospholipase A2 activity and generate ROS as by-products during arachidonic acid oxidation.

However, the main source of ROS is represented by O_2_^•−^ production by the membrane-bound enzyme NADPH oxidases. O_2_^•−^ production by the phagocyte enzyme is a well-known phenomenon, which helps to kill bacterial intruders [72].

The phagocyte NADPH oxidase is composed of two membrane proteins gp91phox (cytochrome b558 heavy chain, later designated as NOX2, which is the catalytic subunit of the enzyme) and p22phox, three cytosolic proteins p67phox, p47phox, and p40phox, and a small GTP binding protein Rac [73]. In resting cells, the enzyme is dormant, and its components are distributed between the cytosol and plasma membrane. Bacterial infection induces translocation of the cytosolic components to the phagosome membrane where they associate with cytochrome b558 and give rise to the catalytically active NADPH oxidase [73].

The presence of NOX2 homologs was firstly suggested by the observation that O_2_^•−^ is produced in a NADPH-dependent manner in nonphagocytic cells, in which NOX2 is not expressed [74]. To date, five NOX isoforms (NOX1, NOX2, NOX3, NOX4, and NOX5) and two related enzymes, DUOX1 and DUOX2, have been reported and most, if not all, isoforms were targeted to cellular membranes.

The NOX proteins constitute the only enzyme family with the sole function of producing ROS. These proteins have different regulation and specific subcellular localization and generate distinct ROS [75]. NOX1, present in smooth muscle cells and other vascular cells, NOX2, present in endothelial and phagocytic cells, and NOX3, found in the brain and inner ear, generate O_2_^•−^. NOX4, constitutively expressed and active in vascular smooth muscle and endothelial cells, is responsible for the basal production of H_2_O_2_. NOX5, identified in human immature lymphatic tissues and endothelial cells, produces H_2_O_2_ in a Ca^2+^ dependent fashion. The DUOX1 and DUOX2, originally isolated from the thyroid, produce H_2_O_2_ that oxidizes iodide during thyroid hormone synthesis.

The phagocyte NADPH oxidase, when activated, generates quantities of O_2_^•−^ and H_2_O_2_ accounting for a significant fraction (10–90%) of total oxygen consumption of neutrophils, macrophages, and microglia, but the contribution of such and other NADPH oxidases to total cellular ROS production in resting or during activation is less clear [23].

It has also been reported that arachidonic acid [76] and its metabolites generated by lipoxygenase [77] and cyclooxygenase [78] stimulate the generation of ROS by NOXs, thus revealing the existence of an interconnecting signaling system between eicosanoids and NOXs.

### 2.6. ROS Production by Lysosomes

Although lysosomes are involved in several functions, until recently they were considered as pure sites for terminal degradation of macromolecules [79].

On rat liver membranes, flavins, ubiquinone, and a b-type cytochrome [80] form a functional electron transport system, starting with the donor NADH and ending to acceptor O_2_ through the sequence FAD→cytochrome b→ubiquinone [81]. The role of this redox chain is to support proton accumulation within lysosomes [81] to maintain an optimal pH for the acidic hydrolases [79]. The electron transport chain appeared to give rise to ^•^OH radical, which required the transfer of three electrons to molecular oxygen, whereas O_2_^•−^ was not detected. This does not exclude the intermediate existence of O_2_^•−^, because the acid pH-milieu inside lysosomes favors spontaneous dismutation of O_2_^•−^ to H_2_O_2_, which is cleaved by intralysosomal ferrous iron into ^•^OH [82].

### 2.7. RNS Production

NO^•^ is produced from the metabolism of the amino acid, L-arginine. The enzymes catalyzing this process, known as nitric oxide synthases (NOS), convert L-arginine into L-citrulline and NO^•^ by a 5-electron oxidation of a guanidine nitrogen of L-arginine [83].

To date, three isoforms of nitric oxide synthase have been identified. Two isoforms, neuronal NOS (nNOS; type I NOS) and endothelial NOS (eNOS; type III NOS), are expressed constitutively and regulated by the interaction of Ca^2+^ with calmodulin [84]. The other isoform, inducible-NOS (iNOS; type II NOS), is induced in response to infection, inflammation, or trauma and is not regulated by Ca^2+^ because it forms a complex with calmodulin at very low concentrations of Ca^2+^ [84].

NO^•^ generated by the NOS isoforms located in different cell types plays different roles. NO^•^ generated by nNOS in neurons serves in communication between nerve cells, whereas the free radical generated by iNOS in macrophages and smooth muscle cells contributes to their killing mechanism, and NO^•^ generated by eNOS in endothelium, brain, and heart relaxes blood vessels and maintains normal blood pressure [84].

The subcellular distribution of NOS is dynamically regulated so that the enzymes are exposed to different concentrations of ROS depending on where in the cell they are localized. For example, eNOS is mainly found in plasma membranes of cardiac and endothelial cells and, in both cells, it is localized at the caveolae of the sarcolemma and T tubules, where it is associated with caveolin, the structural protein of caveolae [85]. However, as a result of different stimuli, eNOS shuttles between caveolae and distinct intracellular sites and it is likely that the selective movement of eNOS serves to determine specific responses to the agonists [86].

Interestingly, the study of the subcellular localization of iNOS showed that during sepsis a substantial amount of the enzyme of the rat hepatocytes localizes to peroxisomes [52], but subsequently it was found that only monomeric iNOS is associated with peroxisomes. Peroxisomal iNOS could be reactivated* in vitro*, but it had a lower specific activity than iNOS in the soluble pool [87]. Thus, uncoupled or deficient iNOS may be targeted to the peroxisome, even though it is possible that iNOS plays a role in the regulation of peroxisomal function. Furthermore, there is growing evidence supporting the existence of mitochondrial NOS (mtNOS) and its involvement in the regulation of mitochondrial as well as cellular functions in several tissues [88].

Some functions of NO^•^ in signaling and regulation of cell function are performed through cGMP-independent pathways including those involving mitochondria [89]. At physiological concentrations, most mitochondrial effects of NO^•^ are exerted on the respiratory chain. First, NO^•^ competes with O_2_ for the binding site at the binuclear center of cytochrome *c* oxidoreductase, leading to a reversible inhibition of cytochrome *c* oxidase activity [90]. Secondly, NO^•^, reacting with respiratory Complex III, inhibits electron-transfer and enhances O_2_^•−^ production [91]. NO^•^ also gives rise to protein nitrosation, reacting reversibly with the nucleophilic centers in protein thiol residues [92], and mitochondria, treated with NO^•^ donors, exhibit* S*-nitrosation and inhibition of Complex I [93]. Moreover, the reaction of NO^•^ with O_2_^•−^, which is formed by the mitochondrial respiratory chain, leads to the switch from reversible inhibition of cellular respiration by NO^•^ to the pathological inhibition of mitochondrial function by ONOO^−^ [94].

## 3. Role of ROS Sources in Oxidative Stress and Tissue Dysfunction

Tissue oxidative damage and consequent dysfunction shown in various pathological conditions depend on increased cellular production of ROS and RNS or on impaired removal of such species. Extensive information is available on mitochondrial ROS production and its relationship with mitochondrial oxidative damage and dysfunction. Because mitochondria are required for oxidative energy production and multiple biosynthetic reaction pathways in aerobic cells, it is understandable that a disturbance of mitochondrial function can lead to impaired cell function and development of several pathologies [22].

However, the larger available information and relevance of mitochondrial function for cell viability do not make mitochondria the main source responsible for tissue dysfunction in conditions of oxidative stress. Indeed, even increases in reactive species production by other cellular sources can cause cellular alterations. On the other hand, available data about ROS and RNS production have mostly been obtained using isolated organelles or cellular cultures and, until recently, the direct measurement of ROS in living systems remained difficult due to the lack of adequate methodology. The recently introduced fluorescent protein-based probes for H_2_O_2_ and GSH/GSSG redox state [95] will likely facilitate reliable organelle-specific ROS measurements, but presently we are not able to provide a well-founded answer to the question concerning the role played by the various sources of reactive species in tissue dysfunction.

### 3.1. Mitochondria

Normally, the rate of mitochondrial ROS generation is rather low and results in minimal damage, because mitochondria have a highly efficient antioxidant defense system able to scavenge a large number of the ROS produced. However, in several circumstances, high rates of ROS production occur, so that a substantial part of oxidants may escape the scavenging systems and compromise important mitochondrial functions. Moreover, even though it is extremely unlikely that ^•^OH radicals can be released by mitochondria, oxidative damage to components of cytoplasm and other cellular structures can result from mitochondrial leakage of other ROS, such as H_2_O_2_ that is able to readily cross mitochondrial membranes and reach such structures where, in the presence of Fe^2+^ ligands, it can generate ^•^OH radical.

Information on the role of an increased ROS production in decline of mitochondrial function and cellular derangement is supplied by experimental work dealing with myocardial ischemia-reperfusion (IR) injury.

Myocardial ischemia occurs when myocardial oxygen demand exceeds oxygen supply. Unless reversed, this situation results in irreversible tissue injury and myocardial infarction. Although restoration of blood flow is necessary to salvage ischemic tissues, it may create another form of myocardial damage, termed “reperfusion injury” [96], which is partly due to the generation of toxic oxygen radicals [97].

Initially, O_2_^•−^ was postulated to be the species responsible [98], but now it is clear that the several ROS and RNS are involved in reperfusion injury [99]. ROS are produced in reperfused myocytes from several sources, including xanthine oxidase, NADPH oxidase, and mitochondria. ROS may be produced by xanthine oxidase which is activated during hypoxia [98]. NADPH oxidases account for an important part of the ROS formed during ischemia-reperfusion [100]. However, there is now strong evidence that mitochondrial ROS generation plays a critical role in damaging cellular components and initiating cell death.

The proposal that the respiratory chain is a major source of ROS during reperfusion of ischemic myocardium [97] was supported by the observation that a generation of oxygen radicals was induced* in vitro* upon reoxygenation of mitochondria isolated from hearts that had been subjected to ischemia [101]. Further support was obtained demonstrating by electron paramagnetic resonance that resumption of mitochondrial oxidative phosphorylation upon postischemic reflow can be a source of oxygen radicals in intact rabbit hearts [102].

Because mitochondrial ROS generation depends on the degree of reduction of the autoxidizable electron carriers, the increased reduction of the respiratory chain associated with ischemia promotes ROS generation upon the respiration resumption. It was proposed that ROS generation is induced by interaction with ubisemiquinone, which accumulates in mitochondria during ischemia because of respiratory chain inhibition [103]. This ROS generation ends rapidly when the mitochondrial components of the respiratory chain are reoxidized. However, it is long-lasting mitochondria that have accumulated large amounts of reducing equivalents, so that the severity of reperfusion-induced oxidative damage and mitochondrial dysfunction should increase with ischemia duration. In fact, it was determined that mitochondrial function impairment was enhanced when coronary occlusion periods increased [104] and mitochondria lipid peroxidation increased gradually with ischemia duration [105]. These changes were well related to a gradual decline in mitochondrial respiration, which reflected damage to electron transport chain components. It was also shown that, after reperfusion of ischemic heart, functional recovery of the tissue was inversely correlated to mitochondrial derangement [105], supporting the idea that heart performance is strongly conditioned by mitochondrial functionality. Further support was provided by the observation that the antioxidant protection of mitochondrial function was associated with decreased impairment of cardiac function following ischemia-reperfusion [106]. Thus, mitochondria are a site of reperfusion-induced oxidative damage, whose severity increases with ischemia duration.

It is likely that mitochondrial oxidative damage and dysfunction is due to ^•^OH radicals produced within mitochondria. These oxyradicals are highly reactive, short-lived species and are expected to cause damage at or near the site of formation. Therefore, they may inactivate components of the respiratory chain, enzymes of the Krebs cycle, and other mitochondrial proteins, leading to mitochondrial dysfunction.

NOS stimulation [107] and inhibition of mitochondrial function by both NO^•^ [108] and ONOO^−^ [109] upon ischemia-reperfusion were reported, suggesting that the reduction in mitochondrial respiration induced by ischemia-reperfusion also depends on increased RNS production.

RNS involvement in reperfusion-linked alteration in mitochondrial and tissue function was demonstrated studying ischemia-reperfusion in the presence of the NOS inhibitor, N^*ω*^-nitro-L-arginine (L-NNA) [110]. Indeed, L-NNA improved heart functional recovery and mitochondrial respiration protecting mitochondria from the oxidative and nitrosative damage.

### 3.2. Strengthening of Mitochondrial Oxidative Damage

In light of the aforementioned results, the mechanism which, during reperfusion, causes tissue damage and dysfunction appears to be a positive feedback loop. Indeed, the concomitance of reflow-mediated perturbations, such as NOS activation and increased ROS production, strengthens mitochondrial damage and dysfunction thus leading to increased tissue derangement. Furthermore, there is evidence that other mechanisms, involving mitochondria, are able to alter the tissue susceptibility to stressful conditions, leading to pathological consequences.

A mechanism of ROS production strengthening is the process named ROS-induced ROS release (RIRR) [111]. RIRR is generated by circuits requiring mitochondrial membrane channels, including nonspecific mitochondrial channels called the mitochondrial permeability transition (MPT) pores [112] and the inner membrane anion channel (IMAC) [113].

A condition that leads to RIRR is the exposure to high oxidative stress resulting by an increase in ROS that reaches a threshold level that triggers the opening of MPT pore. Under oxidative stress, mitochondrial Ca^2+^ overload takes place, which depresses mitochondrial function [114] and triggers several processes, including MPT pore opening [111]. This, in turn, causes collapse of mitochondrial membrane potential and transient increase in ROS generation [111]. In addition to ROS effects in mitochondria where the RIRR is originated, the ROS release into cytosol, which seems to occur through IMAC [115], can lead to RIRR activation in the neighboring mitochondria. ROS trafficking between mitochondria could constitute a positive feedback mechanism for enhanced ROS production potentially leading to significant mitochondrial and cellular injury.

Although externally generated O_2_^•−^ and H_2_O_2_ can readily cross mitochondrial membranes, it is likely that H_2_O_2_ is the messenger molecule leading to whole cell RIRR because of its longer lifetime in the cytosol and higher permeability in membrane lipids [116]. In addition, it is conceivable that a phenomenon similar to RIRR can also depend on NO^•^. Indeed, NO^•^ diffuses from mitochondria to cytosol, as well as from cytosol to mitochondria, a process called mitochondria-cytosol NO^•^ cross talk [117]. Within mitochondria, NO^•^ is able to act as an inducer of permeability transition [118], through a direct effect on the MPT pore and an indirect effect secondary to oxidative phosphorylation inhibition [119].

Excess oxidants may also augment mitochondrial ROS by upregulating the expression of the lifespan regulator, the 66-kDa isoform of growth factor adaptor Shc (p66^Shc^) protein, which has been implicated in the development of aging and aging-related diseases [120]. The protein resides mainly in the cytosol [121], with a small fraction localized in the mitochondrial intermembrane space [122]. The protein is kept by thioredoxin (TRX) 1 and glutathione in the inactive reduced state. However, stress factors, including ROS, can increase the expression of the protein that is activated by thiol oxidation which causes a dimer-tetramer transition [123]. Activated p66^Shc^ translocates to the mitochondrial intermembrane space where it associates with cytochrome c producing H_2_O_2_, which can trigger MPT pore opening [121]. The importance of p66^Shc^ *in vivo* has been demonstrated by observation that knockout of p66^Shc^ increases lifespan, reduces H_2_O_2_ generation, and enhances survival to oxidant stress [120].

Mitochondrial ROS production can be increased by mitochondrial fission. As it is known, mitochondrial shape can be modified by fusion and fission, resulting in elongated, interconnected mitochondrial networks and fragmented, discontinuous mitochondria, respectively [124].

Specific changes in mitochondrial shape suggest that morphology and function of mitochondria are closely linked, so that loss of fusion or division activity results in dysfunctional mitochondria [125]. An explanation for the importance of mitochondrial fusion could be the need for exchange of intermembrane space and matrix contents between mitochondria, so that defects and transient stresses may be partially buffered. On the other hand, mitochondrial division should create organelles of the appropriate size for transport along actin or microtubule networks [125].

The fission process seems to have a remarkable impact on ROS metabolism. Indeed, it seems that oxidative stress causes mitochondrial fragmentation via differential modulation of mitochondrial fission-fusion proteins [126], leading to reduced respiratory capacity and enhanced ROS production [127]. It has also been suggested that changes in mitochondrial network structure provide an example of ROS-mediated ROS generation where ROS play a role in mitochondrial fission to augment ROS generation from restructured mitochondria [128].

NO^•^ appears to play opposite roles in mitochondrial fission-fusion. Indeed, it may enhance mitochondrial fragmentation and cell death in neurodegenerative diseases by its effects on dynamin-related protein-1 (Drp1), which promotes mitochondrial fission [129]. In myogenesis, NO^•^ has the opposite effect promoting the fusion of mitochondria into an elongated network by inhibiting Drp1-mediated fission [130].

### 3.3. Other Cellular ROS Sources

Until recently, the functional significance of ROS sources different from mitochondria has received lesser attention. However, in recent years, greater attention has been turned to the potential role of ROS produced by outer sources in cell signaling and dysfunction.

**Figure 2:**
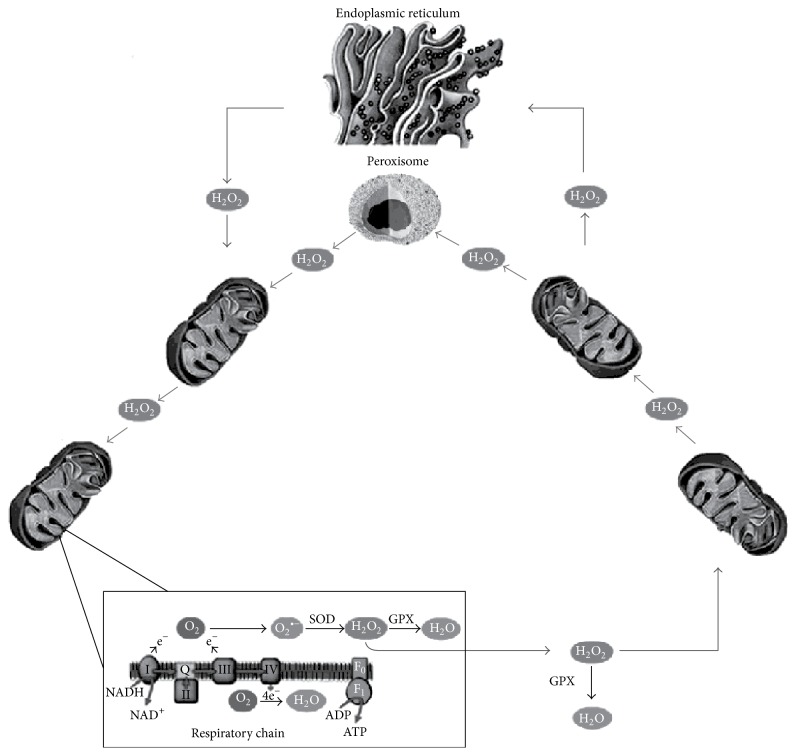
ROS propagation among mitochondria and other ROS sources. Mitochondrial ROS production can cause collapse of mitochondrial membrane potential and increase in ROS generation. ROS produced in only small number of mitochondria can influence neighboring mitochondria and other cellular organelles, eventually propagating the ROS surge to the whole cell (modified from Venditti et al. [116], with permission).

Moreover, there are reasons to think that ROS, released by mitochondria, interact not only with other mitochondria but also with other sources of ROS. It is now apparent that there is a substantial interplay between ROS sources, so that activation of one can lead to activation of the others, resulting in RIRR that further increases ROS production and oxidative stress (Figure 2).

#### 3.3.1. Peroxisomes

Normally, peroxisomes display mechanisms to maintain the equilibrium between production and scavenging of ROS, but in some situations antioxidant system capacity is overwhelmed. One such situation is the increase in peroxisome numbers stimulated by a heterogeneous class of chemicals, known as peroxisome proliferators, whose effects are mediated by peroxisome proliferator activated receptors (PPARs) which belong to the family of nuclear transcription factors [131]. Whereas the expression of the genes of the lipid *β*-oxidation, particularly of acyl-CoA oxidase, is induced 10–30-fold depending on compound and dosage, the catalase does not exceed 1-2-fold induction [132]. The disproportionate increase of H_2_O_2_-generating oxidases in comparison to H_2_O_2_-scavenging catalase was suggested to be responsible for oxidative stress leading to the development of hepatic tumors in rodents treated with peroxisome proliferating compounds [133].

The central event in the carcinogenesis seems to be the activation of PPAR*α*, because PPAR*α*−/− mice, fed a diet containing a potent nongenotoxic carcinogen, are refractory to both peroxisome proliferating effect and carcinogenesis [134]. This is consistent with resistance to the carcinogenic effect of peroxisome proliferators of primates and humans, which have low hepatic levels of PPAR*α* [135].

Peroxisomes rely heavily on cross talk with other subcellular organelles, notably mitochondria, to further metabolize the end products of their metabolism [136]. Peroxisomes and mitochondria also share an intricate redox sensitive relationship [137] and seem to cooperate in the maintenance of cellular ROS homeostasis. It has been suggested that when mitochondrial H_2_O_2_ generation increases and the system constituted by glutathione peroxidase (GPX) and glutathione reductase (GR), limited by GSH and NADPH levels, is unable to cope with the increased H_2_O_2_, the peroxide that escapes across the mitochondrial membrane may be degraded by catalase in the peroxisomes [138].

Actually, the very high content of catalase inside peroxisomes suggests that these organelles may serve as an intracellular sink for H_2_O_2_. This idea is supported by the finding that overexpression of catalase in pancreatic islets of transgenic mice produces a marked protection of islet insulin secretion against H_2_O_2_ [139]. However, other studies indicate that peroxisomes represent a potential source of oxidative stress, causing cell damage or modulating redox sensitive pathways [140], and disturbances in peroxisomal redox metabolism lead to mitochondrial oxidative stress [141]. For example, human fibroblasts treated with a catalase inhibitor not only exhibit high levels of cellular H_2_O_2_, protein carbonyls, and peroxisomal numbers but also increase mitochondrial ROS levels and decrease mitochondrial aconitase activity and inner membrane potential, demonstrating that peroxisome oxidative imbalance elicits oxidative damage throughout the cell and in particular to mitochondria [142]. Peroxisome dysfunction also has a profound impact on mitochondrial function, as demonstrated by the observation that Pex5p (peroxisomal cycling receptor) knockout mice possess increased levels of mitochondria, which show structural abnormalities and alterations in the expression and activity of respiratory chain complexes [143]. Mitochondrial oxidative phosphorylation is also impaired, with consequent increase in mitochondrial ROS generation, in X-linked adrenoleukodystrophy, the most common peroxisomal disorder [144].

#### 3.3.2. Endoplasmic Reticulum

ROS production in endoplasmic reticulum is normally neutralized by antioxidant system components, but in some conditions there is an excessive production of ROS leading to oxidative stress. An example is provided by the accumulation of unfolded and misfolded proteins in the ER lumen, a condition called ER stress, which has been associated with several diseases, such as neurodegenerative disorders, stroke, bipolar disorder, cardiac disease, cancer, diabetes, and muscle degeneration [145].

It is well documented that only properly folded proteins can be exported to the Golgi apparatus for further modification and translocated to their destined sites, while misfolded or incompletely folded proteins are retained in the ER [146]. ER stress can be due to extracellular stimuli and changes in intracellular homeostasis, including ER Ca^2+^, glycosylation, energy stores, redox state, and expression of proteins that are prone to misfolding. In response to ER stress, cells activate an adaptive response, the unfolded protein response (UPR), to resolve the protein folding defect. The UPR attenuates protein synthesis to reduce protein load, induces ER chaperone genes to accelerate protein folding, and degrades misfolded proteins by the ER-associated degradation (ERAD) machinery [24]. However, when ER stress is too severe or prolonged, processes need to be elicited to remove overstressed cells [147].

The ER protein folding homeostasis and ER redox state are closely linked since disulfide bond formation in the ER lumen is highly sensitive to altered redox balance so that both reducing and oxidizing reagents disrupt protein folding and cause ER stress [148]. During oxidative protein folding, the thiol groups on cysteines of substrate peptides are oxidized and H_2_O_2_ is generated as a by-product. During ER stress, dysregulated disulfide bond formation and breakage may lead to oxidative stress by generating large amounts of H_2_O_2_ and depleting ER GSH [149].

Studies have indicated that ROS, produced in the ER during ER stress, subsequently caused mitochondrial dysfunction, impairing the respiratory chain, and increase mitochondrial ROS production particularly when ER stress is severe or sustained. Indeed, the increase in ROS levels causes a Ca^2+^ influx from the ER into the cytoplasm through the ER-localized channels and a large portion of the ion is taken up by the mitochondria resulting in ROS production [145]. Mitochondrial ROS can in turn increase further the ER stress response thereby amplifying ROS accumulation [150]. Thus, it seems that the ER is placed in a vicious cycle where ER stress can be caused by oxidative stress and increases the perturbed redox state. This process is likely favored by the existence of close ER-mitochondria contacts [151], which facilitate the ROS shift between organelle compartments.

ROS production can also be amplified when exposure to a variety of foreign compounds, such as phenobarbital (PB), increases the P450 levels. Induction by PB causes proliferation of the smooth endoplasmic reticulum with incorporation of the structural gene products (the P450s) into microsomal membrane [152]. In rat liver, increases in P450 levels, microsomal and mitochondrial protein content, and alteration of mitochondrial composition were reported [153]. PB-induced increase in P450 levels enhances H_2_O_2_ production by NADPH-supplemented liver microsomes [154] and gives rise to an increase in peroxidative reactions, involving the whole cell [153].

Although these results suggest a damaging role of P450 induction in hepatic tissue, a consequence of the high rates of production of ROS by P450 is its own labilization and subsequent rapid inactivation. Indeed, ROS, generated by some cytochrome P450 forms, are able to lead to oxidative inactivation of the P450 themselves modifying apoenzymes or oxidizing the heme groups. Attack on sulfur-containing amino acids results in the conversion of P450 into P420 [155], whereas attack on the heme moiety leads to its breakdown and loss of microsomal P450 [156]. Such a loss was observed during microsome catalytic turnover under the action of oxygen species generated at the hemoprotein active center [157]. The cytochrome P450 self-inactivation is important in situations in which increases in P450 levels occur due to uptake by the organism of enhanced amounts of xenobiotics.

Although P450 induction serves to help “detoxication” of xenobiotics, it can be considered an adaptive response that has survival value for the organism, as it can also increase “toxification” [158]. First, cytochrome P450 generates toxic chemically reactive intermediates from relatively unreactive compounds. Moreover, because cytochrome P450 inducibility is generally higher than the conjugating enzyme inducibility [159], there is a potential imbalance between the rate of generation of chemically reactive intermediates and their rate of inactivation and removal. The reactive metabolites that are not conjugated and the ROS released by P450 may attack proteins, membrane components, or nucleic acids, leading to cytotoxicity, mutations, and cancer [160]. Therefore, P450 inactivation may be considered a mechanism to prevent cellular accumulation of high levels of the enzyme. Moreover, it appears to be part of a concerted adaptive response to oxidative stress, consisting of repression of ROS-generating systems and induction of antioxidant defenses, as observed in human hepatoma HepG2 cell lines, expressing CYP2E, the ethanol-inducible cytochrome P450 [161].

#### 3.3.3. Lysosomes

Lysosomes contain the major pool of low mass redox-active intracellular iron, arising from the intralysosomal degradation of iron-containing proteins, such as ferritin, and iron-rich organelles, such as mitochondria [162]. Iron accumulation predisposes lysosomes to oxidant-induced damage and rupture with consequent cellular injury. Indeed, some H_2_O_2_, formed outside the organelles and escaping the extralysosomal degradation, can diffuse into lysosomes and, together with that formed directly in the lysosomes, can generate ^•^OH radicals by Fenton reaction. The ensuing oxidative damage on the lysosomal membranes leads to membrane permeabilization with release to cytosol of hydrolytic enzymes and low mass iron. This can relocate to other cellular sites, causing site-specific ^•^OH production and oxidative damage in conditions of H_2_O_2_ production.

It seems that H_2_O_2_ formation and lysosomal destabilization are important for the radiation-induced cellular injury and death [163], which for a long time have been considered to depend on ^•^OH formation due to radiolytic cleavage of water. Indeed, radiation, besides ^•^OH radicals, produces significant amounts of H_2_O_2_ [164], which can enter the lysosomal compartment and cause membrane permeabilization. Employing a model of irradiated murine histiocytic lymphoma (J774) cells [163], it was found that the cells irradiated twice demonstrated progressive lysosomal damage from 2 h after the second irradiation, which in turn resulted in extensive cell death [163]. Irradiation-induced lysosomal disruption and cell death were significantly reduced by incubation with desferrioxamine conjugated with starch which forms a stable complex with iron and renders the metal inactive [163]. The protective effect of iron chelators against ionizing radiation damage was subsequently confirmed on several cell lines previously exposed to salicylaldehyde isonicotinoyl hydrazone, a lysosome targeted iron chelator [165].

#### 3.3.4. NADH-Oxidase

Although multiple sources of ROS during ischemia-reperfusion have been identified, convincing evidence supports NADPH oxidases as important contributors to oxidant generation in several tissues, including cardiac tissue [166]. However, depending on the phase of IR injury, NADPH oxidases can be either detrimental or protective, shown to have a double-edged role. Low ROS levels are cardioprotective in pre- and postconditioning therapies, while high ROS levels are deleterious and lead to cardiomyocyte death [167]. In addition to the ROS level, the types of NOX expressed in cardiomyocytes and their localization are also important in determining the cell fate. In cardiomyocytes, three NOXs are expressed, NOX1 and NOX2, predominantly localized to the plasma membrane, and NOX4, which is constitutively active and is localized on the intracellular membranes of organelles [168]. Although the role of NOX isoforms in ischemia-reperfusion has not yet clarified, a study showed that deleterious effects of NOX1 and NOX2 occurred during reperfusion phase in agreement with the idea that oxygen supply during reperfusion provides substrate for NOX-mediated ROS generation [169]. Moreover, indications were also obtained that NOX2 generated higher levels of ROS than NOX1, leading to direct myocardial damage [169].

Several examples of interplay between mitochondrial and NOX-derived ROS have been reported [170]. In the whole, they suggest the presence of a feedforward cycle in which NADPH oxidases increase mitochondrial ROS that further activate cytoplasmic NADPH oxidases and increase cellular ROS production.

The NADPH oxidase involvement in the ROS-induced ROS production is very well documented. For example, exposure of smooth muscle cells and fibroblasts to exogenous H_2_O_2_ activates these cells to produce O_2_^•−^ via a NADPH oxidase [171]. This mechanism could help to explain why micromolar concentrations of H_2_O_2_ cause oxidant-mediated injury to many different types of cells during chronic oxidative stress. Moreover, H_2_O_2_-induced NADPH oxidase activation in nonphagocytic cells could be an important mechanism by which the degree of oxidative stress, as well as the subsequent cellular damage, is amplified during inflammatory disorders.

It was also observed that endothelial levels of xanthine oxidase, a source of H_2_O_2_ and O_2_^•−^, depend on NADPH oxidase [172]. The study of the mechanism showed that H_2_O_2_ was able to induce transformation of xanthine dehydrogenase into xanthine oxidase [173]. The observation that increase in O_2_^•−^ production was a late effect of cell exposure to H_2_O_2_ suggested that the peroxide not only stimulated conversion of xanthine dehydrogenase into xanthine oxidase but also activated the NADPH oxidase, leading to prolonged H_2_O_2_ production and sustained xanthine dehydrogenase conversion [173].

## 4. ROS as Signaling Molecules

ROS are now appreciated as signaling molecules that regulate a wide variety of physiological functions. Indeed, they play crucial roles in gene activation [174], cellular growth [175], and modulation of chemical reactions in the cell [176]. They also participate in blood pressure control [177], are mediators in the biosynthesis of prostaglandins [178], function in embryonic development [179], and act as signaling molecules within the individual cell and among cells during their lifespan [180].

An important development in the field of ROS beneficial effects was the discovery that, in organisms from simple bacteria to complex mammals, ROS are able to induce redox sensitive signal cascades leading to increased expression of antioxidant enzymes. The increase in effectiveness of the antioxidant defense system provided by this genetic response enables cells to survive an oxidant exposure that would normally be lethal.

In mammals, gene transcription determining cell survival can be activated by ROS in two ways: either via transcription factors, which can interact directly with specific DNA motifs on promoters of target genes, or via activation of mitogen-activated protein kinase cascades, which in turn activate transcription factors that trigger target gene transcription [181].

There are two ideas concerning the mechanism by which ROS initiate cellular signaling, namely, modification of target protein molecules and changes of intracellular redox state [182], even though the distinction between them is not easy.

While ROS, such as ^•^OH, may cause irreversible damage to macromolecules with low specificity, the main target of a mild oxidant, such as H_2_O_2_, is thiol groups of protein cysteine residues. Oxidation of these residues forms reactive sulfenic acid (-SOH) that can form disulfide bonds with nearby cysteines (-S-S-) or undergo further oxidation to sulfinic (-SO_2_H) or sulfonic (-SO_3_H) acid. These modifications result in changes in structure and/or function of the protein. With the exception of sulfonic acid and to a lesser degree sulfinic acid the modifications are reversible by reducing systems such as thioredoxin and peroxiredoxins [183].

The cytosol redox state is normally achieved by the “redox-buffering” capacity of intracellular thiols, such as GSH and thioredoxin, which counteract cellular oxidative stress by reducing H_2_O_2_. The high ratios of reduced to oxidized forms are maintained by the activity of GSH reductase and TRX reductase, respectively. Accumulating evidence suggests that GSH and TRX participate in cell signaling processes. GSH can regulate redox signaling by alterations both in the level of total GSH and in the ratio of its reduced to oxidized forms, while TRX can regulate the activity of some proteins by directly binding to them [182].

Whatever the mechanism by which ROS initiate cell signaling, there is increasing evidence that ROS cellular levels are strongly linked to the regulation of cellular antioxidant levels. A well-known example of this phenomenon is Nrf2. This transcription factor regulates the expression of several antioxidant and detoxifying genes by binding to promoter sequences containing a consensus antioxidant response element [184]. In turn, the subcellular localization and hence activity of Nrf2 are at least in part regulated by its interaction with specific reactive cysteine residues on an inhibitory protein called Keap1 [185].

Interestingly, ROS not only are involved in the regulation of the expression of antioxidant genes but also interact with critical signaling molecules such as MAP kinases, PI3 kinase, PTEN, and protein tyrosine phosphatases, to initiate signaling in several cellular processes, including proliferation and survival [186].

### 4.1. ROS Role in Protection against Tissue Excessive Dysfunction

An enhanced ROS production in a cellular site can involve further ROS production by other cellular sources giving rise to a self-destructive phenomenon as well as the propagation of a fire in a room containing inflammable material. If, as in the fire example, it occurs to confine the more dangerous focuses and, above all, avoid that the fire reaches the other rooms and the entire house catches fire, the cell exposed to oxidative stress should be equipped with mechanisms able to avoid its death or that of surrounding cells, which should lead to excessive tissue dysfunction.

Several results indicate that ROS-linked mechanisms are operative to provide tissue protection against excessive dysfunction, in which mitochondrial systems seem to play a major role.

#### 4.1.1. Mitoptosis

High mitochondrial ROS production triggers opening of the MPT pore leading to potentially significant mitochondrial and cellular injury. However, in many cases mitochondrial swelling due to megachannel opening is a signal for programed mitochondrial destruction [187], a phenomenon called mitoptosis [188], which was proposed to represent a line of the antioxidant defense [70].

It is well-established that the mitochondrial population is heterogeneous with regard to its characteristics because it consists of fractions with different properties [189]. Studies on liver mitochondrial fractions, resolved by differential centrifugation, suggested that the light fractions, with low respiratory activity, contained transitional forms in the process of development into the heavy mitochondrial structures with high respiratory activity [190]. The heavy fraction also exhibited the lowest antioxidant level [190, 191] and the highest rates of H_2_O_2_ production and susceptibility to Ca^2+^-induced swelling [190]. It is interesting that conditions leading to increased ROS production, such as exercise [192] and cold exposure [193, 194], decrease the amount of heavy mitochondria and increase that of light mitochondria in rat muscle and liver, respectively. This suggests that, in rat tissues, conditions of increased ROS production favor the substitution of the oldest ROS-overproducing mitochondria with neoformed mitochondria endowed with a smaller capacity to produce free radicals [190]. If so, the mechanism that, during oxidative stress, enhances the swelling of Ca^2+^-loaded mitochondria constitutes a negative feedback loop. In fact, the perturbation itself, represented by an enhancement in ROS generation, should lead to accelerated mitoptosis, thus limiting tissue oxidative damage during oxidative stress.

#### 4.1.2. Autophagy

Autophagy is a cytoprotective process by which organelles and bits of cytoplasm are sequestered in double-membrane vesicles, called autophagosomes, and subsequently delivered to lysosomes for hydrolytic digestion [195]. Autophagy serves as an adaptation strategy for stress conditions, such as amino acid starvation, unfolded protein response, or viral infection, and it is widely accepted that it regulates cell homeostasis by adjusting organelle number and clearing damaged structures. However, if autophagy prevents or promotes cell death and if it is a selective or nonelective process are still controversy questions. It is likely that autophagy can be involved in both survival and death. It assures cell survival when it removes damaged organelles that might activate programed cell death as apoptosis (type I cell death) [196]. On the other hand, it promotes cell death when it is excessive and deregulated, since enzymes leaking from lysosomes, such as cathepsins and other hydrolases, can initiate mitochondrial permeabilization and, eventually, apoptosis [197].

Clearly, autophagy leading to cell death is not selective, whereas the controversy persists about autophagy developing as a survival process. The observation that autophagosomes often contain various cytoplasmic elements, including cytosolic proteins and organelles [198], suggested that autophagy is a nonspecific form of lysosomal degradation. However, subsequent observations showed that the autophagy can be a selective process in which specific proteins or organelles are delivered to the autophagosome for degradation [197].

Such selective types of autophagy include selective degradation of mitochondria (mitophagy) [197], peroxisomes (pexophagy) [199], endoplasmic reticulum (reticulophagy) [200], or even nucleophagy during which parts of the nucleus are specifically degraded by an autophagic process [201].

Mitochondria subjected over time to multiple attacks become damaged and possibly dangerous to the cell, so that elimination of such mitochondria is essential to protect cells from the harm due to their disordered metabolism. The view that autophagic processes can remove damaged and dysfunctional mitochondria was directly confirmed by experiments in which selected mitochondria inside living hepatocytes were subjected to laser-induced photodamage [202]. Mitochondrial depolarization and inner membrane permeabilization seemed to be required for autophagy signaling [202] suggesting involvement of MPT pore opening and swelling in the mitophagy.

A growing body of evidence now suggests that processes of autophagy and/or apoptosis involving other cellular organelles are able to protect tissues in conditions leading to oxidative stress.

Peroxisomes were the first organelles for which selective organelle degradation by autophagy was described [203]. Studies on selective degradation of peroxisomes in methylotrophic yeasts showed that when grown on methanol as the carbon source, yeast species included several large peroxisomes containing the enzymes necessary to assimilate methanol. When methanol grown cells were shifted to a different carbon source, such as glucose or ethanol, whose metabolism did not involve these peroxisomal enzymes, pexophagy occurred [199]. Hence, this autophagic process occurred during a rapid intracellular remodeling process to remove organelles containing enzymes no longer needed for methanol utilization.

More recent studies indicate that autophagic degradation of peroxisomes in yeast also occurs as part of cellular housekeeping [204]. It was observed that, during chemostat cultivation of wild type* H. polymorpha* cells, entire peroxisomes were constitutively degraded by autophagy during normal vegetative growth, likely to enable the cells to rejuvenate the peroxisome population.

There is convincing evidence that ER stress and reticulophagy induction are strongly linked. For example, it was reported that, during unfolded protein response to ER stress, induced by DTT, portions of the ER were sequestered by double-membrane vesicles, similar to autophagosomes [200]. The presence of ribosomes on the outer membrane of these structures suggested a role of the ER as a membrane source for this type of autophagosomes, which subsequently fused with the vacuole releasing its content for degradation. This process was highly selective as cytosol and other organelles were not included into the vesicles [200]. Not much is known about ER degradation and its role in ER maintenance, but it is possible that this selective pathway serves to degrade damaged portions of the ER or resize it after the folding stress induced enlargement.

Although numerous factors and signaling pathways contribute to autophagy induction in different cellular contexts, ROS are indicated to be essential signals to activate autophagy stress by several stimulating conditions [205]. ROS appear to be implicated in the regulation of autophagy through distinct mechanisms, depending on cell types and stimulation. Autophagy, in turn, can reduce ROS production removing ROS-overproducing organelles.

It seems that ROS, generated from both mitochondria and NADPH oxidases, activate autophagy to protect cells from nutrient starvation, dysfunctional mitochondria, cell death, and invading pathogens [206]. The involvement of ROS as signaling molecules in starvation-induced autophagy was demonstrated for the first time showing that starvation triggered accumulation of ROS, most probably H_2_O_2_, which was necessary for autophagosome formation and the resulting degradation pathway. The oxidative signal is partially dependent on phosphatidyl inositol 3 kinase (PI3K), which plays a critical role in the early stages of autophagosome formation. Furthermore, a direct target for oxidation by H_2_O_2_, the cysteine protease Atg4, has been identified [207]. Whereas mitochondria were found to be involved in starvation-induced autophagy [208], activation of antibacterial autophagy is due to NADPH oxidase that generates ROS necessary for targeting of the autophagic protein LC3 to the phagosome [205].

#### 4.1.3. Autophagic and Apoptotic Cell Death

Autophagy usually promotes cell survival but when cellular damage is pronounced and survival mechanisms fail, death programs are activated in response to oxidative stress. The activation of the autophagic pathway beyond a certain threshold has recently been found to directly promote cell death by causing the collapse of cellular functions as a result of cellular atrophy (autophagic or type II cell death) [209]. This form of autophagic cell death, which is necessary under certain conditions, such as in apoptosis-defective cells [210], is activated in response to oxidative stress in nervous cells, as shown by oxidative stress and autophagic death induced in human neuroblastoma SH-SY5Y cells [211] and sympathetic neurons [212] by dopamine and growth factor deprivation, respectively.

Autophagy can also lead to the execution of apoptotic or necrotic (type III cell death) programs, likely via common regulators such as proteins from the Bcl-2 family [196]. Apoptotic death is an adaptive process, allowing for renewal of the organism constituents and life maintenance, which, differently from necrosis, is a form of cell death that causes minimal damage to surrounding cells.

Apoptosis plays an important role in the elimination of unnecessary, damaged, or diseased cells during the whole lifespan and particularly during embryogenesis, when a majority of newly formed cells undergo programed cell death. Although both types of cell death have been linked to autophagy, cell death does not necessarily result from a previous autophagic process. Depending on the cellular context and death trigger, apoptosis and necrosis either cooperate in a balanced interplay involving autophagy or are employed by cells in a complementary way to facilitate cellular destruction. A further complication results by the observation that apoptosis, necrosis, and autophagy are often regulated by similar pathways, engage the same subcellular sites and organelles, and share initiator and effector molecules.

An example is supplied by the cellular response to MPT pore opening. This process provides a common pathway leading to mitophagy, apoptosis, and necrosis [213]. With low intensity stress, limited MPT leads to mitoptosis, which is followed by the elimination of degraded mitochondria. Recent studies have shown that removal can occur through two different processes which rid cells of damaged mitochondria, the formation of mitoptotic bodies which are then extruded from the cell [214] or selective autophagy [215]. With increasing stress MPT involves an increasing proportion of mitochondria and cellular response progresses from mitoptosis to apoptosis driven by mitochondrial release of cytochrome c and other proapoptotic factors. This event occurs if permeabilization affects a great number of mitochondria, in the absence of excessive reduction in ATP levels, and can purify tissue from cells that produce large amounts of ROS [70]. Lastly, when extreme stress causes MPT pore opening in virtually all cellular mitochondria, ATP levels drop, and, because of bioenergetic failure, neither autophagy nor apoptosis can progress, and only necrotic cell death, which is not dependent on energy supply, ensues [197].

Another example involves cellular responses to ER stress, which include the activation of UPR, autophagy, and cell death [216]. These processes are not mutually exclusive, and there is significant cross talk between these cellular stress responses. Autophagy upregulation during ER stress is a prosurvival response directed to removal of unfolded proteins, protein aggregates, and damaged organelles, which is triggered in order to relieve the stress and restore ER homeostasis [217]. However, prolonged or unresolved ER stress results in apoptotic program activation [218]. Sometimes, the effect of autophagy on cell survival in ER stress also depends on the tissue type. In colon and prostate cancer cells, ER-induced autophagy protects against cell death, whereas in normal colon cells autophagy does not alleviate ER stress but rather contributes to ER-induced apoptosis [219].

Recent studies have shown several pathways that mediate the interplay between autophagy and apoptosis providing mechanistic insight into the network regulating both processes [220]. However, the mechanism by which ER stress induces apoptosis is not fully delineated, even though it is clear that mitochondria-dependent and independent cell death pathways can mediate apoptosis in response to ER stress.

In ER-mitochondria-independent cell death pathway, caspases are required for apoptosis, and some members of this family of proteases are associated with the ER. In rodents, caspase-12 is ER-membrane proapoptotic protease specifically activated upon prolonged UPR. Several pathways involve caspase-12 activation in cell apoptosis after ER-induced stress. A pathway triggers apoptosis by ER stress-specific caspase cascade leading to the caspase-9 and caspase-3 activation in cytochrome c and apoptotic protease activating factor-1 (Apaf-1) independent manner [221].

ER stress can also trigger apoptosis by mitochondria-dependent pathways [222] which can share actors with mitochondria-independent pathways. The phosphorylation of inositol-requiring enzyme 1 (IRE1) leads to the phosphorylation of c-Jun N-terminal kinase (JNK) that, in turn, can activate p53 transcription factor. The p53-induced expression of a protein, the Bcl-2 interacting mediator of cell death (Bim) [223], leads to the formation of the BAX/BAK pore on the outer mitochondrial membrane and cytochrome c release resulting in activation of the canonical apoptosis pathway.

Whereas the relationship between mitochondrial oxidative stress and cell death is well-established [224], that of peroxisomes in cell death pathways is just beginning to emerge. In light of the strong impact of peroxisome dysfunction on mitochondria, it is possible that peroxisome-derived mediators of oxidative stress have an influence on mitochondria that would have profound implications for cell fate.

In fact, it has been reported that excess ROS generated inside peroxisomes quickly perturbs the mitochondrial redox balance and leads to remarkable mitochondrial fragmentation [225]. In a recent study using targeted variants of the photosensitizer Killer Red it has been showed that the phototoxic effects of peroxisomal Killer Red induce mitochondria-mediated apoptotic death and that this process is inhibited by targeted overexpression of antioxidant enzymes, including peroxisomal glutathione S-transferase kappa 1, superoxide dismutase 1, and mitochondrial catalase [226].

Over the last decades, the intricate molecular events underlying the process of apoptosis have been elucidated [227]. It is apparent that crucial steps involve mitochondrial release of proapoptotic factors, although the exact mechanisms involved in this release are less well understood. In this regard, it seems that, in some circumstances, the release into the cytosol of lysosomal constituents may be an initiating event in apoptosis and that mitochondrial release of proapoptotic factors might be a consequence of earlier lysosomal destabilization [228].

The destabilization of the lysosomal membrane is due to its vulnerability to oxidative stress, because agents, which induce oxidative stress, such as H_2_O_2_ [229] and radiation [230], also promote lysosomal membrane rupture. Different cell types but also cells of the same type and lysosomes of individual cells exhibit different lysosomal resistance to oxidative stress [231]. It has been suggested that such a resistance mainly depends on the capacity to degrade H_2_O_2_ before it reaches the acidic vacuolar compartment, resistance to ROS of lysosomal membranes, and lysosomal content of redox-active iron [231]. Lysosomal membrane permeabilization per se triggers intracellular formation of ROS, a process which can be mediated by the action of lysosomal proteases, such as cathepsins B and D, which leak into the cytosol [208]. These enzymes affect mitochondria inducing further cytochrome c release and activation of caspase-mediated cell death [208, 228]. The involvement of lysosomes and their iron content in radiation-induced cell death is supported by the observation that cells are significantly protected from radiation damage if exposed to iron chelators [163, 165]. Conversely, the involvement of lysosomal proteases in cell death is supported by the observation that apoptosis inhibition by cyclosporin A-induced block of MPT pore opening favors the development of a necrotic form of cell death, which is attenuated by ROS scavengers and inhibition of cathepsin D activity [232].

## 5. Harmful and Beneficial Effects of ROS during Exercise

It is well documented that acute physical exercise can produce significant damage, including alterations in membranes of mitochondria and sarcoplasmic and endoplasmic reticulum [233–236] in skeletal muscles and other tissues. The contraction form most damaging to skeletal muscle is that in which the muscle is contracting while being lengthened (eccentric contraction). During such a contraction, disruption of cytoskeletal structures, loss of muscle force generation, and influx of phagocytic cells and neutrophils into the damaged fibers occur [237].

Different from acute exercise, aerobic physical activity regularly performed (training) induces adaptive responses in the whole organism and particularly in the cardiorespiratory and musculoskeletal systems [238], which lead to an increased ability to perform prolonged strenuous exercise [235]. Moreover, it has several healthy effects, including the maintenance of insulin sensitivity and cardiorespiratory fitness, so that it is able to prevent type 2 diabetes [239] and coronary heart diseases [240], and can also be used as adjunctive therapy in the treatment of patients with diabetes [241] and chronic heart failure [242].

To date, the idea that the opposite effects of acute exercise and training are in great part due to the ability of ROS to play a dual role in animal organisms is widely shared. In fact, it is well documented that, during a single session of prolonged aerobic exercise, ROS production increases and this can lead to cellular damage and dysfunction. On the other hand, some results suggest that the ROS produced during each session of a training program can act as factors inducing cellular adaptations to exercise.

During physical activity several ROS sources can be activated contributing to the oxidative damage and/or to the adaptive processes. It is reasonable to imagine that an initial source of ROS can activate the ROS release from other sources, inducing a positive feedback loop. In the next sections, we will point out our attention to what is known about production, sources, and double action of ROS during acute exercise and training.

## 6. Acute Exercise

### 6.1. ROS Production

ROS involvement in the tissue damage found after prolonged aerobic exercise was suggested in the late 1970s [243], but there is still no direct evidence that ROS production increases during exercise. Electron spin resonance (ESR) spectroscopy was able to furnish direct information of the presence of free radical species, but such a technique allows obtaining information on the ROS production after exercise. Davies et al. [244] first reported that signals of free radicals were enhanced in rat muscle and liver after a bout of exhaustive running. Subsequently, increased generation of free radical signals was observed in rat heart after an acute bout of exhaustive endurance exercise [245]. The idea that free radical activity might increase after extensive muscular activity was also confirmed on skeletal muscles subjected to electrical stimulation [246]. Using ESR spectroscopy in conjunction with the spin tapping technique, enhanced free radical concentration in human serum following exhaustive exercise was found [247, 248] which was prevented by ascorbic acid supplementation [249].

Indirect information on ROS production during acute exercise has been obtained by the changes in indexes of oxidative damage to lipids, proteins, and DNA and in the cellular redox state. A plethora of information exists concerning the increase in such indexes in various animal species, including human, but here we focus our attention on what is available in literature on rat.

Lipid oxidation can be evaluated measuring tissue levels of oxidized lipids, such as lipid hydroperoxides, or those coming from their degradation, such as malondialdehyde (MDA) and 4-hydroxyl-2-nonenal. Numerous studies showed increase in lipid oxidation markers in skeletal [244, 250–254] and cardiac muscle [251–253, 255], liver [244, 250–254, 256], brain [257], erythrocytes [258], and kidney [254] of untrained rats after acute exercise.

Exercise-induced increases in protein carbonyl content, a marker of protein oxidative damage, were reported in rat skeletal [253] and cardiac muscle [253, 255], liver [253, 256], and plasma [259]. Furthermore, increases in 8-hydroxy-2′-deoxyguanosine (8-OH-dG), a marker of oxidative damage to DNA, were also found in rat skeletal [260] and cardiac [255] muscle, liver, and lung [260] after acute exercise.

Some studies reported that the extent of oxidative damage depends on duration or intensity of exercise. One such study showed that exhaustive maximal exercise caused plasma MDA increase while short periods of submaximal exercise (less than 70%  VO_2max_) reduced lipid peroxidation [261]. Liver oxidative damage indexes did not change after 5 h of swimming and increased after 8 h of exercise [192]. Another study showed that an acute bout of a moderate as well as high intensity exercise led to an increase in malonaldehyde and lipid hydroperoxide levels in red vastus, white vastus, and soleus muscle [262]. The study also showed that when intensity of exercise was considerably decreased, lower MDA levels were found [262].

Reduced glutathione (GSH), a thiol-containing tripeptide playing a vital role in maintaining cells in the reduced state and in protecting tissues from oxidative stress [263], is involved in reducing radicals arising from a variety of antioxidants, such as *α*-tocopherol and ascorbic acid, to the native structure [9]. GSH is oxidized to glutathione disulfide (GSSG) donating a pair of hydrogen atoms. The ratio of GSH to GSSG is used as an indicator of intracellular GSH redox status. A decrease in GSH/GSSG ratio suggests that the production of ROS goes over the reducing capacity of GSH and other antioxidants. Therefore, change of GSH redox status has been used as a footprint of oxidative stress during exercise. Several studies showed that prolonged exhaustive exercise promoted oxidation of GSH to form GSSG in the blood [264], liver [253, 264], heart [253, 265], and skeletal muscle [253, 264] of rats, even though it is possible that the GSH redox state in the muscle is not altered by short term exercise. Indeed, it was reported that physical exercise at submaximal level determined a progressive depletion of liver GSH to about 20% of the levels found in sedentary rats which persisted for several hours following the cessation of exercise [266]. However, skeletal muscle appeared to be spared by this severe depletion phenomenon, whereas the levels of plasma glutathione exhibited a transient increase at the beginning of the exercise bout followed by a linear decrease with increased running time [266]. In liver, the early decrease in GSH level during exercise did not depend on increased ROS production, even though it could be one of the factors inducing liver oxidative stress. Evidence that a decrease in liver GSH content precedes oxidative stress was supplied by the finding that prolonged exercise led to increased lipid peroxidation and decreased GSH content, while exercise of shorter duration was not able to induce oxidative stress in liver, although it reduced GSH content [192]. The liver GSH reduction can be due to several factors. It has been suggested that the exercise linked increase in plasma levels of glucagon, vasopressin, and epinephrine stimulates hepatic efflux of GSH which is delivered to tissues, such as skeletal muscle that necessitates a larger tripeptide supply and its uptake from the plasma [267, 268].

### 6.2. Sources of ROS during Physical Activity

Notwithstanding most work indicates that exercise increases ROS production in rat tissues, the debate about the cellular sources of such ROS is still open. Several intracellular sources of free radicals have been identified and it is possible that all contribute to the increased ROS production during exercise even though the extent of their contribution can depend on several factors including the type of tissue and exercise.

Mitochondria, NADPH oxidase, and xanthine oxidase are considered the main ROS sources during exercise but it is necessary to point out that reports concerning other cellular sources are scarce or lacking.

#### 6.2.1. Mitochondria

Mitochondria were long considered the main source of ROS in the cell during physical activity. Initially, this belief stemmed from the widespread idea that in tissue free radical production was closely related to oxygen consumption [269]. Since over 90% of the oxygen consumed by a mammal is utilized in the mitochondria, which appear to generate free radicals in all tissues studied [270], it was speculated that the increase in muscular oxygen consumption during exercise was associated with an increase in free radical production by the respiratory chain localized in the inner mitochondrial membrane [269].

During muscle contraction, ADP concentration increases and stimulates mitochondrial oxidative phosphorylation [271], shifting the mitochondrial respiration nearer to State 3 than to State 4 oxygen consumption. Therefore, the close link between ROS production and O_2_ consumption should require that the percentage of total electron flow escaping from the respiratory chain to reduce O_2_ to superoxide radical (the mitochondrial free radical leak) is not modified during the transition from State 4 to State 3 happening during muscle contraction. However, this idea is theoretically and experimentally inconsistent. The rate of the mitochondrial ROS generation is related to the degree of reduction of electron carriers able to donate electrons to oxygen, and such a degree of reduction decreases during transition from State 4 to State 3 [272]. In agreement with such prediction, measurements performed on isolated mitochondria show that ROS mitochondrial release is higher in State 4 and in all conditions when the rate of electron-transfer is lowered [272].

Nevertheless, results obtained measuring ROS release from mitochondria isolated from tissues of animals that exercised suggest that mitochondrial ROS release might increase during prolonged aerobic exercise not only in muscle but also in other tissues, such as liver and heart [272].

An increased release of ROS during basal and stimulated respiration, with respiratory substrates linked to Complex I (pyruvate plus malate) or Complex II (succinate), was reported for mitochondria isolated from the muscles of the hind limbs [273], gastrocnemius (red portion), heart, and liver [253] of rats subjected to prolonged swimming. Increased ROS release was also reported following prolonged treadmill running [274] in heart mitochondria. The increased ROS release was associated with an increase in the levels of oxidative stress markers in mitochondria isolated from cardiac and skeletal muscles and liver [253] of rats subjected to prolonged aerobic exercise. It was also accompanied by alterations in mitochondrial functionality. Indeed, exercise increased State 4 respiration in liver, muscle [253], and heart [253, 274] mitochondria and decreased State 3 respiration in liver and muscle mitochondria [253]. Such results suggest the possibility that, whatever the initial source of ROS during exercise is, such ROS can damage mitochondrial components inducing both functionality impairment and increasing ROS release. It was initially proposed that the ROS formation during exercise could involve the loss in the cytochrome oxidase activity and a consequent increase in the electron pressure within the respiratory chain [275]. The finding that in hind limb muscle mitochondria exercise-induced change in mitochondrial respiration is not associated with changes in the cytochrome oxidase activity [273] seems to exclude such a possibility suggesting that oxidative modifications of other components of mitochondrial membrane are involved. Oxidative modifications of lipids and proteins located in the inner mitochondrial membrane could be responsible for the increase in State 4 respiration. The increase in State 4 respiration rate represents a compensatory response to the increased leak of protons back in the mitochondrial matrix. It has been proposed that adenine nucleotide translocase (ANT) and uncoupling proteins (UCPs) are involved in proton conductance of mitochondrial membrane [276]. However, the observation that in skeletal muscle ANT expression is not affected by acute exercise [277] excludes that ANT is responsible for the increase in State 4 respiration induced by exercise. Moreover, it is known that an uncoupling protein 1 (UCP1) catalyzes inducible proton conductance in brown adipose tissue (BAT) [278], and exercise upregulates UCP1 homologue expression in skeletal [279] and cardiac muscle [274]. However, whether in tissues differently from BAT UCP1 homologues are responsible for mitochondrial basal or inducible proton conductance is yet controversial [280].

More support is available for the idea that the observed enhancement in State 4 respiration is due to high production of ROS and RNS, which seem to be able to affect proton leak through an indirect mechanism. Both O_2_^•−^ [281] and ONOO^−^ [282] increase mitochondrial proton leak by enhancing the extent of peroxidative processes [282, 283]. Therefore, it is conceivable that the increase in State 4 respiration induced by exercise may be due to a lipid peroxidation-mediated increase in proton leak.

The decrease observed in State 3 respiration can be due to a direct action of ROS and/or RNS. Indeed, damage to respiratory chain components by ROS [284] and inhibition of mitochondrial function by ONOO^−^ [109] have been reported. The decline of the respiration rate in mitochondria from rats that exercised is likely to involve ONOO^−^, which during exercise could be formed in greater amount and cause irreversible inhibition of many mitochondrial components different from cytochrome aa3 [285].

Inhibition of mitochondrial function and increase in ROS release could also be due to the increase in mitochondrial Ca^2+^ content, which occurs in skeletal muscle after prolonged exercise [286] and leads to oxidative phosphorylation inhibition [287]. The Ca^2+^ effect on mitochondrial function results from induction of mitochondrial permeability transition (MPT) [114], which leads to degradation of the heaviest mitochondrial subpopulation characterized by high respiratory capacity and susceptibility to Ca^2+^-induced swelling [190].

Following long-lasting exercise, a transfer of damaged mitochondria in the lightest fraction happened in rat liver, as demonstrated by the increase in protein percent content in such a fraction and its decrease in the heaviest one [192], whereas a similar transfer did not happen in skeletal muscle [273]. This agrees with the observation that acute exercise enhances mitochondrial resistance to Ca^2+^ overload in human skeletal muscle [277]. The Ca^2+^ tolerance of mitochondria after exercise could be due to the maintenance of relatively high levels of GSH, which should prevent mitochondrial membrane potential collapse [288] which precedes MPT pore opening. Although this can appear as a protective mechanism, it slows removal of ROS-overproducing mitochondria thus enhancing exercise-induced muscle oxidative damage and dysfunction.

#### 6.2.2. NADPH Oxidases

In the cells of skeletal muscle, the isoform NOX2 is expressed [289] and protein subunits have been identified in transverse tubules and triads obtained from rabbit skeletal muscle but not in sarcoplasmic reticulum vesicles [290]. Some authors suggested that this is one of the main sources of ROS during* in vitro* muscle contraction or electrical stimulation [291, 292]. Moreover, it was demonstrated that muscle contraction increased O_2_^•−^ in cytosol and subsequently in mitochondria, suggesting that NADPH oxidase could be a potential primary source of ROS production during muscle contraction [293]. It is conceivable that during muscular activity the increased activity of NADPH oxidase can produce excessive ROS release which can contribute to damage cellular components. However, it is also possible that such a release can have beneficial roles. The increased level of ROS induces changes in intracellular calcium levels which are the result of oxidative modification of calcium channels or other proteins involved in calcium signaling [294] and it has been suggested that NOX2 ROS production may be necessary for the excitation-contraction coupling process [290].

The mechanism of activation of NADPH oxidase has not been completely defined but a recent paper suggests that this can involve the increase in ATP release. Electrical stimulation of adult muscular fibers isolated from the muscle flexor* digitorum brevis* activates a voltage gated L-type calcium channel (Cav1.1) with each depolarizing event. This activation induces ATP release via pannexin-1 (PnX1) channel which colocalizes in the transverse tubules with Cav1.1 [295]. These events, in turn, trigger a signaling cascade where, through ATP activation of a purinergic receptor (P2Y), phosphatidylinositol 3-kinase-*γ* and phospholipase C and consequently protein kinase C, which activates NOX2 oxidase and ROS release [296], are activated. It is conceivable that a similar activation can happen also in* in vivo* muscle because an increase in ATP concentration has been found in the interstitial muscular space after exercise and contraction [297, 298].

#### 6.2.3. Xanthine Oxidases

Studies performed using XO inhibitors suggested a potential role of enzyme XO as a source of oxidative stress, during ischemia-reperfusion in various tissues, such as intestine, heart, lung, kidney, and liver [98, 299, 300]. In particular, XDH/XO activity in liver has been reported to be relatively higher with respect to other organs [301, 302]. On the other hand, it has been demonstrated that liver blood flow during severe exercise may be reduced to half of normal, indicating that exercise can induce ischemia or hypoxia in the hepatic tissue [303]. These findings would suggest that XO produces free radicals which may influence the function of hepatic cells during and/or after strenuous exercise. Furthermore, inosine and hypoxanthine produced by the skeletal muscle during severe exercise have been reported to be taken up by the liver via the blood stream and oxidized to uric acid mainly after exercise [304]. According to these observations, it was shown that the liver has a substantially higher risk of oxidative stress following a single bout of exhausting exercise, rather than during the exercise itself [305].

It has been reported that xanthine oxidase produces O_2_^•−^ in the contracting rat [306] and human skeletal muscles [307]. However, there is controversy about the role xanthine oxidase plays in O_2_^•−^ production of human skeletal muscle during contraction, because such a muscle appears to possess low amounts of xanthine dehydrogenase or oxidase [308], even though these enzymes are present in associated endothelial cells. It has been speculated that postexercise oxidative stress in mouse skeletal muscles may be due to the conversion of XDH into XO in capillary endothelial cells [309] and enhanced adenosine 5′-triphosphate (ATP) degradation together [310].

More recently, it was found that the administration of allopurinol, XO inhibitor, did not prevent the increase in protein and lipid oxidative stress markers in rat plasma, erythrocytes, and gastrocnemius muscle due to swimming exhaustion [311]. Moreover, in rats treated with allopurinol alone, a similar increase in protein and lipid oxidative stress markers in erythrocytes and gastrocnemius muscle was found. The combination of allopurinol and exercise appeared to increase protein oxidative damage in plasma and protein and lipid oxidative damage in erythrocytes. Interestingly, allopurinol provoked a marked reduction in physical performance as demonstrated by the 35% decrease in the swimming time to exhaustion [311]. This result is in contrast with another work reporting a lack of effects of allopurinol administration on the time to exhaustion in rats performing treadmill running [305]. The discrepancy can be due to the different type of exercise and/or to the different doses of allopurinol. However, it is interesting that notwithstanding allopurinol seems to have intrinsic antioxidant properties being a potent hydroxyl radical scavenger [312, 313], it either did not change or decreased the time to exhaustion, differently from what was found administering antioxidants before an exhaustive exercise [314].

Another study [315] reported that, following acute exercise (60 min treadmill running 27 m/min, 5% incline), skeletal muscle oxidized glutathione (GSSG) significantly increased in allopurinol- and vehicle-treated rats despite XO activity and uric acid levels were unaltered. This suggests that XO was not the source of ROS during exercise. In the whole, the available data obtained by XO inhibition suggest that the enzyme can be a source of ROS during exercise but not the main.

## 7. Training

### 7.1. Effects of Training on Tissue Oxidative Damage

Contrary to acute exercise, aerobic exercise training induces adaptations which reduce liver [316] and skeletal [317] and cardiac muscle [318] oxidative damage of lipids and proteins. Such adaptations also render tissues less susceptible to the oxidative damage induced by conditions leading to oxidative damage. Indeed, training attenuates lipid and protein oxidative damage and glutathione depletion in rat heart subjected to ischemia-reperfusion [319]. Moreover, training prevents lipid peroxidation increase induced by moderate intensity exercise in rat liver and muscle [250]. However, other studies suggested that training does not affect the extent of lipid peroxidation due to exhaustive swimming but, delaying the rate of the peroxidative reactions, allows trained animals to sustain the activity for a longer period before the fatigue becomes limiting [251, 252].

It is clear that training exerts protective effects reducing oxidative damage of tissues and increasing their resistance to oxidative challenges. Such effects seem to be associated with increased cellular antioxidant defenses. Several studies examined the effect of training on the activities of antioxidant enzymes. Much of these studies may not be directly compared to each other because of the differences in experimental design, animal model, and analytical procedures. However, in the whole they show that training results in an increase in skeletal [320] and cardiac [321] activity of antioxidant enzymes, such as superoxide dismutase, glutathione peroxidase, glutathione reductase, and catalase, even though some studies failed to find enhanced antioxidant activity after training.

The training effect on liver antioxidant enzymes has been less studied and the results are rather contrasting. On the other hand, it was previously reported that liver total antioxidant capacity was increased by training as well as those of skeletal and cardiac muscle [251, 252].

Surprisingly, there are few studies concerning the effect of training on antioxidant enzyme expression. However, increases induced by training in CuZnSOD mRNA abundance in rat liver and heart [322] and Cu, ZnSOD, and MnSOD protein level only in some muscles but not in others [322, 323] were reported.

It is likely that training exerts its protective effects also decreasing H_2_O_2_ production, even though scarce information is available on training impact on cellular ROS sources. The rate of H_2_O_2_ release was decreased in liver [316], skeletal muscle [317, 324], and heart [318] mitochondria from rats trained to swim and in heart mitochondria [325] from rats trained to run. Conversely, no effect on H_2_O_2_ release was found in skeletal muscle mitochondria following voluntary wheel training [326].

Measurements of H_2_O_2_ release rate in the presence of respiratory inhibitors suggested that training reduces the concentration of the autoxidizable electron carriers located at Complexes I and III in the liver [316] and muscle [317] mitochondria and that of the autoxidizable electron carrier located at Complex III in the heart mitochondria [318]. However, other swim training-induced adaptations can contribute to the reduction of the H_2_O_2_ release rate found in the mitochondria isolated from liver heart and muscle. These could include the increased activity of the H_2_O_2_ metabolizing enzyme GPX, which is coupled with the increase in GR activity, in the mitochondria of the three tissues [316–318]. Conversely, it is unlikely that the decrease in H_2_O_2_ release is due to increased uncoupling of the inner mitochondrial membrane, since that training reduces mRNA expression of uncoupling protein 3 in skeletal muscle and uncoupling protein 2 in skeletal and cardiac muscles [327].

The effects of exercise training on NADPH oxidase activity are limited but some data suggest that exercise training is able to modulate NADPH oxidase activity. For example, it has been reported that exercise training mitigates age-related upregulation of NOX2 subunits gp91phox and p47phox in rat heart [328], reduces microvascular endothelial NOX content in muscle biopsies from vastus lateralis of obese men [329] but not of lean men [330], and downregulates NADPH oxidase expression in obese rats [331]. Studies using XO inhibition by allopurinol show that such an inhibition produces complex effects that are not yet fully elucidated so that the relative data are not conclusive and do not allow us to point out the relevance of the ROS produced by XO for the training adaptations.

### 7.2. Mitochondrial Biogenesis

The improved cardiovascular function and aerobic capacity elicited by aerobic exercise training require an increased tissue metabolic activity. The first proof that the increase induced by the training in metabolic capacity was due to the increased tissue mitochondrial protein content was obtained in the skeletal muscle [332, 333]. Subsequent studies confirmed this finding [317] and showed that swim training increased mitochondrial protein content also in other tissues such as liver [316] and heart [318]. However, the changes in mitochondrial protein content seemed to differ in various tissues and were associated with different changes in the metabolic capacity of tissues and mitochondria. Indeed, in the skeletal muscle swim training increased tissue metabolic capacity by inducing a moderate increase in the tissue content of the mitochondrial population characterized by a lightly reduced aerobic capacity [317]. In liver, swim training did not induce increases in tissue metabolic capacity because the modest increase in mitochondrial population content was balanced by the reduction in mitochondrial metabolic capacity [316]. In heart, swim training increased tissue metabolic capacity by enhancing lightly both mitochondrial population content and mitochondrial respiratory capacity [318]. These different responses are consistent with the different functions of liver and muscles as energy supplier and consumers, respectively [334], during aerobic long-lasting exercise.

### 7.3. Mechanisms of Adaptive Response to Exercise

Mitochondrial biogenesis requires that expression of the mitochondrial genome and nuclear genes encoding mitochondrial proteins is finely organized. The process is controlled by the peroxisome proliferator-activated-receptor-gamma coactivator 1 (PGC-1) which regulates the expression of transcription factors such as nuclear respiratory factors 1 and 2 (NRF-1 and NRF-2) [335]. NRF-1 and NRF-2 control the expression of many genes, among which are those implicated in mitochondrial biogenesis, adaptive thermogenesis, glucose and fatty acid metabolism, fiber type switching in skeletal muscle, and heart development [336].

PGC-1 expression increases quickly in muscle cells stimulated to contract [337], in rat skeletal muscle after a single bout of exercise [338] and in human skeletal muscle after endurance training [339]. A study using PGC-1*α* knockout mice showed that in liver PGC-1*α* plays a pivotal role in regulation of cytochrome c and cytochrome oxidase subunit I expression in response to a single bout of treadmill exercise and prolonged exercise training, which suggests that the exercise-induced changes in tissue oxidative capacity are regulated by PGC-1*α* [340].

Increases in PGC-1*α* expression were also found in heart after short term training to treadmill run [341] and in liver [316], skeletal muscle [317], and heart [318] after 10 weeks of training to swimming. In these tissues, the increases in PGC-1*α* expression were associated with an increase in the expression of NRF-1 and NRF-2.

Interestingly, in addition to regulating mitochondrial biogenesis, PGC-1 is able to regulate endogenous antioxidant expression, such as Cu, ZnSOD, MnSOD, and GPX, in skeletal muscle [342, 343]. This coordination of the proliferation of ROS producing organelles with increase in antioxidant levels likely helps to maintain redox homeostasis. In addition, it has been shown that PGC-1*α* promotes mSIRT3 gene expression, which is mediated by ER-binding element mapped to the SIRT3 promoter region [344]. In turn, SIRT3 binds to, deacetylates, and activates mitochondrial enzymes, including MnSOD, through a posttranslational mechanism [345].

PGC-1*α* is also able to regulate the mRNA expression of uncoupling proteins 2 and 3 in cell culture [346], suggesting that PGC-1*α* may also increase the uncoupling capacity and concomitantly reduce ROS production in the mitochondria [343].

Several initiating stimuli, activated during exercise, can contribute to eliciting the PGC-1 gene response. These include (i) increase in cytosolic calcium concentration, which activates various signaling pathways regulated by the calcineurin phosphatase and the calmodulin-modulated kinase, (ii) the decrease in levels of high-energy phosphates, leading to the activation of the AMP-sensitive kinase (AMPK), and (iii) stimulation of the adrenergic system, leading to cyclic AMP synthesis, and activation of protein kinase A and other kinases, such as mitogen-activated protein kinase (MAPK) [342]. However, it is necessary to point out that PGC-1*α* regulation is not only due to variation in expression but also caused by covalent modifications among which are phosphorylation, acetylation methylation, and ubiquitination. Indeed,* in vitro* experiments showed that p38 MAPK and AMPK phosphorylate PGC-1*α* producing a more active protein [342].

It seems that PGC-1 expression is also upregulated by ROS. Indeed, the observation that antioxidant incubation prevents the increase in PGC-1*α* mRNA induced by electrical stimulation in rat skeletal muscle cell culture [347] indicates that increases in ROS may contribute to exercise-induced increases in skeletal muscle PGC-1*α* mRNA content. Thus, the observation that the H_2_O_2_-induced increase in the mRNA content of SOD, catalase, and GPX in PGC-1*α* KO fibroblasts is lower than that in wild type fibroblasts [348] indicates a role of PGC-1*α* in the upregulation of ROS removing enzymes in response to increases in ROS.

Moreover, notwithstanding conflicting results exist in literature [349], it was reported that antioxidant supplementation attenuates the PGC-1*α* expression increase due to training [316–318, 350–352]. It was also reported that vitamin E supplementation prevents the increase in activator and coactivator levels and mitochondrial population adaptation to physical training [316–318]. These results suggest that the ROS produced during each session of exercise training are able to regulate cellular functions acting as signals regulating molecular events crucial for adaptive responses of liver, muscle, and heart.

The role of ROS as signaling molecules in the tissue adaptation induced by training seems to contrast with the oxidative damage and dysfunction elicited by acute exercise. However, this can be explained by differences in extent and temporal pattern of ROS generation. Thus, a moderate, intermittent ROS production during short time periods in a program of graduate aerobic training can activate signaling pathways leading to cellular adaptation and protection against future stresses. In contrast, moderate levels of ROS production over long time periods (e.g., hours) or high levels produced during brief exercise at high intensity may result in structural and functional tissue damage.

## 8. ROS Production and Type 2 Diabetes

Type 2 diabetes and other related diseases, such as metabolic syndrome and coronary heart disease, are a serious problem worldwide [353]. Specifically, diabetes is very closely related to microvascular and macrovascular complications that seriously affect the quality of life and life expectancy of patients. At present, there are around 350 million people worldwide with diabetes, a figure that will rise to 500–600 million over the following years. Diabetes, insulin resistance, and cardiometabolic diseases are associated and constitute an active field of research [354, 355].

Mitochondria are known to produce ATP after metabolization of nutrients and are capable of generating energy. In this sense, it has been demonstrated that mitochondrial dysfunction is characterized by decreased levels of ATP, inhibition of mitochondrial O_2_ consumption, enhanced ROS production, a decrease in the antioxidant content, and alterations in mitochondrial membrane potential (ΔΨ_*m*_). These effects are due mainly to an imbalance between energy intake and expenditure [356]. In fact, a decrease in the activity of the electron transport chain (ETC) complexes or increased uncoupling produced by the activity of uncoupling proteins or the ADP/ATP translocator (also called adenine nucleotide translocase, ANT) can induce changes in ΔΨ_*m*_ that eventually lead to apoptosis [354, 355].

Different factors, both genetic and environmental (diet, exercise, and stress), have been shown to modulate mitochondrial function and alter insulin sensitivity [357, 358]. In this context, the presence of mitochondrial impairment has been demonstrated in different types of leukocytes [359] and tissues such as liver, lung, skeletal muscle, spleen, or heart in type 2 diabetes [360, 361], confirming the relationship between this condition and mitochondrial dysfunction.

Mitochondria are the main source of reactive oxygen species (ROS), as mentioned previously. ROS are key to the development of diabetic complications [362–364], and studies have demonstrated that the use of antioxidants such as lipoic acid (LA) can reduce insulin resistance and ROS production (by improving mitochondrial function) and prevent CVD in humans [365]. Recently, Faid et al. have demonstrated that resveratrol can alleviate diabetes-induced apoptosis by modulating caspase-3 activities, oxidative stress, and JNK signaling [366].

Electron transport chain (ETC) dysfunction is directly related to diabetes and its complications, including retinopathy, nephropathy, and neuropathy [355]. Furthermore, some studies have documented that deleterious genetic mutations related to a reduction in the activity of Complex I can lead to mitochondrial impairment and enhanced ROS production [367]. Therefore, mitochondria-targeted antioxidant therapy has been proposed as a beneficial tool in the treatment of mitochondria-related diseases [368].

Victor's group has performed several studies of type 2 diabetes patients which have shown that oxidative stress and mitochondrial dysfunction occur due to a decrease in O_2_ consumption, Complex I activity, membrane potential, and glutathione levels and an increase in ROS production [359, 369–371], thus confirming mitochondria as a key target for diabetes treatment. In other related pathologies, such as polycystic ovary syndrome (PCOS), in which patients can develop insulin resistance, there is also an impairment of mitochondrial Complex I and an increase in leukocyte-endothelium interactions [372]. This mitochondrial dysfunction increases ROS production, reduces ATP and Ca^2+^, and alters membrane potential and mitochondrial morphology. However, in an animal model of diabetes (db/db mice), mitochondrial and renal function are improved in the presence of mitochondria-targeted antioxidants, such as CoQ10, highlighting the crucial role of mitochondria in the development and pathogenesis of diabetic nephropathy [373]. In addition, Victor's group has demonstrated that mitochondrial dysfunction and, especially, mitochondrial ROS production are related to the development of silent myocardial ischemia and endothelial dysfunction due to increased leukocyte/endothelium interactions [374].

Mitochondria-targeted antioxidants have been shown to have beneficial effects on conditions of oxidative stress. In this sense, MitoQ is an antioxidant which, due to a covalent attachment to the lipophilic triphenylphosphonium cation, is selectively taken up 1000-fold by mitochondria [363, 375]. Chacko et al. demonstrated an example of the beneficial effects of MitoQ on diabetes when they reported that MitoQ decreased urinary albumin levels to the same level as those of nondiabetic controls in a mouse model of diabetic nephropathy (Ins2(+/)-(AkitaJ) mice) [376]. Furthermore, glomerular damage and interstitial fibrosis were significantly reduced in the treated animals, and there was a nuclear accumulation of the profibrotic transcription factors *β*-catenin and phospho-Smad2/3, which was prevented by MitoQ treatment. These results support the hypothesis that mitochondrially targeted therapies could be beneficial for the treatment of diabetic nephropathy.

### 8.1. Insulin Resistance and Mitochondrial Dysfunction

Glucose homeostasis is regulated by insulin. In addition, insulin has important cardiovascular, renal, and neural functions, which may explain why insulin resistance is a risk factor for microvascular complications such as retinopathy, nephropathy, hypertension, and CVD [25].

A series of conditions are related to the development of insulin resistance, such as obesity, changes in lipid and glucose metabolism, chronic inflammation, stress, or other oxidative factors. In these conditions, the appearance of insulin resistance is frequently associated with a diminished capacity of tissues or cells to respond to levels of insulin [377]. This process is related to mitochondrial dysfunction, changes in mitochondrial dynamics, and enhanced ROS production.

In relation to this theory, it has been demonstrated that mitochondrial impairment, oxidative stress, excess energy intake, and lipodystrophy can enhance circulating free fatty acids (FFAs), which can lead to the accumulation of triglycerides, FFAs, and diacylglycerol (DG) in different tissular locations, including liver, skeletal muscle, heart, kidney, and *β*-cells. Furthermore, alterations in cholesterol subfractions, such as an increase in the atherogenic potential of small dense LDL, may be related to several metabolic properties of these particles, facilitating their transport into the subendothelial space [378], reducing LDL receptor affinity [379, 380], and increasing susceptibility to oxidative modifications [378, 381]. The cited studies feed into the idea that small dense LDL are related to arterial damage in patients with dyslipidemia associated with diabetes.

Insulin resistance, mitochondrial dysfunction, and enhanced production of ROS, which act as secondary messengers by activating serine kinases that phosphorylate IRS proteins, modulate the insulin response [377]. In addition, ROS can trigger the inflammatory process by activating IKKb, which phosphorylate IRS-1 [382]. Several studies have demonstrated that insulin sensitivity and mitochondrial function can be modulated by antioxidants, with a subsequent decrease in ROS production and an increase in the expression of UCP2/3 and a decrease in ROS levels [382]. However, the results of the said studies have generated some controversy [383].

Mitochondrial impairment and insulin resistance have also been shown to be related to diminishing levels of mitochondrial oxidative enzymes, which reduce mitochondrial complex activity, alter mitochondrial morphology, and limit mitochondrial number [384]. For example, mitochondrial oxidative capacity can correlate negatively with insulin sensitivity after the accumulation of intramyocellular lipids [385].

During obesity, there is an increase of triglycerides in adipose tissue, and consequently glucose metabolism is altered in other nonadipose tissues. In this sense, it has been speculated that lipodystrophy induces insulin resistance, mitochondrial dysfunction, and type 2 diabetes [386]. In diabetes and obesity, adipocytes can release high amounts of adipokines, such as resistin, leptin, adiponectin, and TNF-*α*, which can regulate metabolic pathways [387]. Furthermore, the number and morphology of mitochondria, as well as the expression of genes involved in mitochondrial biogenesis, are significantly decreased by the energetic alterations that appear as a result [388]. All of these studies support the idea that insulin resistance is present in lipodystrophy, obesity, and type 2 diabetes. This action leads to the accumulation of intracellular fatty acid metabolites (e.g., diacylglycerol, fatty acyl CoAs) in muscle and liver, which triggers the activation of a serine kinase cascade and finally induces defects in insulin signaling and insulin action in these tissues [389].

Oxidative stress, mitochondrial-endothelial dysfunction, and insulin resistance are very common in cardiovascular diseases such as stroke, silent myocardial ischemia, coronary artery disease, or hypertension [390]. In this sense, type 2 diabetic patients exhibit high blood pressure, whose appearance is related to hyperglycaemia [391]. In relationship to this idea, Katz et al. have highlighted that diabetes is associated with a higher prevalence of calcified atherosclerotic plaque in the thoracic arteries [392]. Furthermore, endothelial impairment has been associated with intramyocardial lipid accumulation and glucose intolerance and, eventually, heart failure [393].

Not all organs are specifically protected against oxidative stress. For example, the heart, which has a high metabolic rate and high beta oxidation and ROS production, contains low levels of antioxidants, making it particularly susceptible to oxidative stress, mitochondrial dysfunction, and subsequent structural and functional abnormalities [394].

Mitochondrial oxidative stress damage and changes in the morphology/function of mitochondria have been reported in an animal model of obesity, namely, insulin-resistant obese Zucker rats [395]. Obesity and lipotoxicity can also enhance mitochondrial damage during the development of diabetic retinopathy [396]. Experiments involving transmission electron microscopic analysis of myocardial tissue have demonstrated an increase of abnormal mitochondria in an insulin-resistant rat model [397]. Another study showed an increase in the number of mitochondria in hypertrophied rat hearts under oxidative stress conditions [398]. In disagreement with these studies, others have failed to find changes in the number of mitochondria and their DNA content, while some have even reported reduced numbers in patients and animal models of pathological hypertrophy [399]. In conclusion, these results point out the importance of mitochondria in the heart and would suggest that they enhance CVD, including heart failure, stroke, cardiomyopathy, coronary heart disease, silent myocardial ischemia, and hypertension.

Insulin resistance is related to endothelial dysfunction [400], but the underlying mechanisms are yet to be confirmed. In the endothelium, mitochondria play an essential role by acting as sensors of local alterations in the concentration of O_2_ and as regulators of intracellular Ca^2+^ concentrations [401]. Taking into account that mitochondrial dysfunction is related to endothelial dysfunction, different studies have demonstrated that blockade of ROS generation can improve endothelial function under hyperglycemia conditions [362, 402].

It is generally recognized that endothelial nitric oxide synthase (eNOS) plays a key role in the maintenance of vascular tone and insulin-stimulated NO^•^ production in the endothelium [403]. In fact, poor eNOS activity has been related to insulin resistance, hypertension, and dyslipidemia [404]. Therefore, there is an impairment in the NO^•^ production under insulin resistance conditions, which is related to the appearance of CVD such as coronary artery disease, heart failure, stroke, or silent myocardial ischemia.

Insulin-resistant patients can develop type 2 diabetes when there is impairment in *β*-cells, the result of which is an incapacity to sense glucose properly and release insulin and failed glucose homeostasis. In this sense, mitochondrial activity can modulate the potassium channels (*K*_ATP_) modulating ATP/ADP ratio. *β*-cell function and mitochondrial function are related through the ATP/ADP ratio [405]. Furthermore, mitochondrial Ca^2+^ levels are crucial to the maintenance of insulin secretion, as demonstrated by Han et al. who showed that taurine can enhance the glucose sensitivity of UCP2-overexpressing *β*-cells, probably by enhancing mitochondrial Ca^2+^ influx and subsequently increasing the ATP/ADP ratio and mitochondrial function [406]. Other studies have highlighted the importance of mitochondrial function in glucose homeostasis by using knockout Tfam (Transcription Factor A, Mitochondrial) mice, Tfam being a nuclear DNA-encoded mitochondrial protein, which results in a dramatic mtDNA depletion, a decreased of insulin secretion, reduced *β*-cell mass, and the development of diabetes [407]. In conclusion, all of the abovementioned studies highlight the fact that preserving mitochondrial function is essential for *β*-cell function under oxidative stress conditions and that mitochondrial impairment contributes to the pathogenesis of type 2 diabetes by interfering with insulin action and secretion. Furthermore, high levels of fatty acids can induce mitochondrial dysfunction and impair insulin signaling due to oxidative stress and enhanced ROS production. In summary, we consider that mitochondria should be considered a key target in therapy for insulin resistance in general and diabetes in particular.

Finally, we would like to mention that the possible beneficial effects of RNS and ROS can occur at low levels and can exert different physiological functions. Cells can produce H_2_O_2_, O_2_^•−^, or NO^•^ at physiological levels, but, in the case of diabetes, basal levels of ROS are elevated, and so these ROS and RNS are generally harmful. For example, in basal conditions, leukocytes kill pathogens by phagocytosis after which ROS are released. Basal levels of ROS can also trigger energy production by mitochondria, induce mitogenic responses, or activate the release of cytokines or nuclear transcription factors.

NO^•^ can also modulate vascular pressure, leukocyte adhesion, and angiogenesis. Furthermore, NO^•^ is an important neurotransmitter and is a key mediator of the immune response when generated by activated macrophages. Although different studies suggest that ROS act as secondary messengers, it is clear that they can be harmful when they accumulate and disrupt molecules and tissues [12, 408–410].

## 9. ROS and Neurodegeneration

The brain is composed of two main types of cells: glia and neurons. Glial cells encompass a wide variety of cells including astrocytes, microglia, and oligodendrocytes. Glia cells act as a neuronal support system and are the most abundant cells in the nervous system. Astrocytes support neurons in the brain and regulate the chemical and extracellular environment. They maintain low levels of ammonia and glutamate and produce neuroprotective enzymes. Upon activation, astrocytes repair cellular damage, mount an inflammatory response, and activate microglia. Microglia, the smallest glial cells, repair damage due to injury via phagocytosis. Oligodendrocytes are responsible for axon myelination, a protective layer required for propagation of action potentials and maintenance of intracellular communication.

Neurons (nerve cells) are the basic structural elements of the nervous system. Their primary function is to transmit and receive information through nerve impulses, electrochemical signals that travel down the neuron. Neurons “sense” changes in environmental conditions and respond to such changes via neurotransmission. Neurons consist of three major components: the axon, dendrite, and perikaryon (or soma). The axon, typically ending at a specialized structure called the synapse, carries impulses to distant locations. The dendrite acts as the neuronal “receiver” but can also communicate via neurotransmitters to adjacent neurons. Dendrites become denser during neurogenesis. Small dendrites do not contain organelles, but large dendrites have neurofilaments (only found in neurons), microtubules, ribosomes, and endoplasmic reticulum. The perikaryon is the “metabolic hub” of the neuron. It houses mitochondria, ribosomes, Golgi apparatus, and endoplasmic reticulum, essential machinery in protein synthesis and energy production. Neurons are essential in contributing to emotions, perceptions, and memory and learning. Neuronal damage can alter these functions and ultimately lead to overall brain dysfunction and decline in the cognitive functions previously described. Neurodegeneration, an example of neuronal damage, is the loss of neuronal structures or function. During the aging process, myelin and neuronal loss occur, dendritic length and branching are decreased, and global brain volume is reduced. Consequences of these events include but are not limited to age associated cognitive decline, memory loss, epigenetic changes, reduced autophagy, and synaptic plasticity. Excessive neuronal death resulting in accelerated cognitive decline and memory loss has been observed in those suffering from neurodegenerative disorders. Recent findings from Villeda demonstrated a significant improvement in hippocampal learning exercises and contextual fear conditioning tasks when old mice were given plasma from young mice [411]. Other findings from this study showed an increase in dendritic spine number and synaptic plasticity in the old mice associated with reversal of cognitive decline.

Astrocytes are highly abundant throughout the central nervous system. Due to their extension-like end feet, they cover the free surfaces of neuronal dendrites and soma. Astrocytes also cover the inner surface of the one of the brain's most important meningeal membranes, the pia mater, and all blood vessels in the CNS. More importantly, these end feet surround the brain's capillary endothelial cells of the blood-brain barrier. They participate in neurotransmitter metabolism and play a pivotal role in glutamate uptake to prevent excitotoxicity. Glutamate is then converted into glutamine via glutamine synthetase, in which the basic amino acid is transported into the neuron. Furthermore, astrocytes maintain the pH of the extracellular space and ionic environment. In neurodegeneration, these cells can release cytokines which regulate the inflammatory response.

Microglia act as macrophages in the brain. Phenotypic characteristics include short spiny projections, which become enlarged under ROS conditions. Neurodegeneration is a key promoter of microglial activation; therefore this phenomenon is observed in neurodegenerative disorders including Alzheimer's disease, Parkinson's disease, amyotrophic lateral sclerosis, and Huntington's disease [412]. Microglia also play a role in generating reactive nitrogen species as nitric oxide synthases, iNOS and NOX2, and NADPH oxidase are induced in these glial cells [413]. This NOX2 activation can lead to a respiratory burst of superoxide flooding the mitochondria further contributing to neurodegeneration. In addition to their inflammatory response, microglia also express multiple ion channels, namely, the sodium, proton, voltage gated Ca^2+^ and Cl^−^, and potassium channels. Changes in ionic concentrations may play a role in depolarization and action potential initiation, which can trigger inflammation and neuronal activation.

Activation of these glial cells can have dramatic cellular effects including prompting an inflammatory response. This occurs in the normal aging process due to mitochondrial derived ROS production, which promotes inflammation and cytokine production [414]. This consequence can be observed as cognitive deficits as NF*κ*-B is generated and neuroinflammation occurs. NF*κ*-B serves as a proinflammatory agent and a prosurvival molecule by regulating the inflammatory response. This is an age-dependent process. If the NF*κ*-B pathway is blocked in old mice, a reversal of gene expression occurs [415]. Interestingly, NF*κ*-B is highly associated with RNS as this family of proteins induces nitric oxide synthase, further promoting nitrosative stress. Prostaglandin synthesis is also initiated through the NF*κ*-B signaling cascade, propagating the inflammatory process. Neuroinflammation can be defined as the increased production of a multitude of proinflammatory molecules, mostly notably interleukin-1*β* (IL-1*β*), tumor necrosis factor *α* (TNF-*α*), and transforming growth factor *β* (TGF-*β*). IL-1*β* is a proinflammatory agent that recruits neutrophils as part of the inflammatory response. This cytokine is frequently observed in several neurodegenerative disorders [416]. TNF-*α* is a key regulator of the immune system. It has been shown to be increased in microglia from aged mice in a lipopolysaccharide induced mouse model [417]. TNF-*α* is found predominantly in macrophages. It is released by activated microglia and astrocytes which perpetuates neurodegeneration and neuroinflammation by increasing levels of reactive oxygen species, specifically superoxide. The more the neuronal damage, the more frequent the neuroinflammation as microglia and astrocytes are constantly activated. In addition to the inflammatory properties of TNF-*α*, it directly activates NADPH oxidase which increases levels of superoxide in the cells similar to NF*κ*-B. NOX2 is highly expressed via TNF-*α*, which is linked to excess levels of RNS [418]. TGF-*β* regulates neuroinflammation and apoptosis by releasing inflammatory cytokines and reactive oxygen species [419]. TGF-*β* is mediated by the Smad3 pathway, which inhibits the production of free radicals which are normally promoted in the inflammatory pathway. This pathway is impaired during neurodegeneration and could contribute to the disease progression as neuronal loss and neuroinflammation are observed [420]. As these cytokines have been tested as biomarkers of oxidative stress, a link between ROS, neurodegeneration, and neurodegenerative disorders can be further bolstered.

*Oligodendrocytes.* Oligodendrocytes are vulnerable to oxidative damage as they have a higher ATP requirement than other glial cells, contain low levels of glutathione, a potent antioxidant, and have a high intracellular iron level which can form prooxidants through Fenton chemistry [421]. The main function of oligodendrocytes is myelination. The myelin sheath is a necessary axonal component that increases saltatory conduction of action potentials, thereby stimulating neurotransmission. Demyelinating disorders such as multiple sclerosis exhibit motor function decline due to disruption of action potential propagation from the loss of myelin.

It has been well-established that, in neurodegenerative disease, neurodegeneration can occur as the result of oxidative stress, the imbalance of antioxidant and prooxidant levels [422]. Reactive oxygen species levels increase as a function of age and are even higher in age-related neurodegenerative disorders [423]; therefore oxidative stress can also occur if there is an excess of ROS/RNS production or an antioxidant deficiency [424]. This section of the review will focus on the interconnection between oxidative stress, reactive oxygen and nitrogen species, and neurodegeneration in the aforementioned cellular systems and research directed at neuroprotection, the delay or prevention of neurodegeneration.

### 9.1. Oxidative Stress

Mitochondria are the key source for free radicals [425, 426]. A minute amount of electrons leaks from the mitochondria and reacts with molecular oxygen to form superoxide. Other sources for free radicals can include environmental toxins [427], metal catalyzed reactions, certain enzymatic reactions (e.g., xanthine/xanthine oxidase), and cellular processes. During phagocytosis, oxidants are needed to ingest bacteria, viruses, and other pathogens [428].

#### 9.1.1. Hypochlorous Acid

Hydrogen peroxide can react with a chloride anion to form hypochlorous acid (HOCl) via myeloperoxidase (MPO). Myeloperoxidase is largely present in neutrophils [429] but has also been located in neuronal cells under certain conditions [430]. Hypochlorous acid can further react with nitrogen dioxide (NO_2_^•^) to form nitryl chloride (NO_2_Cl), a potent chlorinating and nitrating oxidant [431]. Phagocytes use HOCl as one of their agents [432]. Hydrogen sulfide (H_2_S) is an inhibitor of HOCl from Cl^−^. Myeloperoxidase in the presence of H_2_O converts Cl^−^ into Cl^+^; therefore hydrogen sulfide may have a protective effect. H_2_S levels are lowered in Alzheimer's disease, a neurological disorder [433]:

(3) $$\begin{array}{c} H_{2}O_{2}+{Cl}^{-}\overset{MPO}{\to }HClO \end{array}$$

#### 9.1.2. Reactive Nitrogen Species

Similar to ROS, reactive nitrogen species are highly toxic [434]. Some examples of RNS, nitric oxide, and nitrogen dioxide will be discussed below. Reactive nitrogen species cause protein nitration by various methods, which can result in protein dysfunction and neuronal loss.

NO^•^ has been shown to play a role in neurodegenerative diseases by acting as a neurotoxin when excessively produced [435]. Hara has shown that glyceraldehyde-3-phosphate dehydrogenase (GAPDH) acts as NO^•^ sensor [436]. NO^•^ is involved in cell signaling pathways immune response and vasodilation. NO^•^ activates protein kinase C (*ε* isoform), which activates a specific family of tyrosine kinases that can stimulate apoptosis [437]. NO^•^ can also bind to glutamate channels and indirectly to calcium and potassium channels [438]. As glutamate is an excitatory neurotransmitter in the cell, depolarization of the membrane occurs. Glutamate binds to the NMDA receptor and an influx of Ca^2+^ enters the cell and causes a disruption in calcium homeostasis. This disruption can eventually lead to cell death.

NO_2_^•^ acts as an outdoor and indoor air pollutant from car emissions, fossil fuels, cigarette smoke, heaters, and gas stoves, just to name a few [439]. NO_2_^•^ is primarily found in the airways of the terminal bronchi; however, NO_2_^•^ may be found in other areas of the respiratory tract. Minimal exposure to NO_2_^•^ results in long morphological changes resulting in possible inflammation, pulmonary edema, and cellular injury [440]. Nitrogen dioxide exposure also leads to an increase in TBARS, a marker for lipid peroxidation in lung tissue, and vitamin E treatment showed a reduced pulmonary injury [441]. NO_2_^•^ serves as an oxidant in inflammation mediated by the peroxidases, eosinophil peroxidase, and myeloperoxidase [442–446]. This gas can oxidize the antioxidant glutathione and increase activity of glutathione reductase and glutathione peroxidase [447, 448]. The depletion of glutathione shifts the balance to the side of nitrosative stress. NO_2_^•^ can also be formed by the oxidation of ONOO^−^, another potent reactive nitrogen species.

## 10. An Overview of Some Neurodegenerative Diseases

Neurodegenerative diseases are a classification of disorders in which neuronal loss and progressive cognitive decline are observed. These two consequences contribute to the memory loss exhibited by those with neurodegenerative disorders. This level of decline is accelerated in contrast to the normal aging process. Depending on the specific disease other characteristics and symptoms include dementia, a decline in motor function, and depression may be evident. The phenomenon of oxidative stress has been well-established in such neurodegenerative disorders as Huntington's disease [449, 450], Parkinson's disease [451], amyotrophic lateral sclerosis [452], and Alzheimer's disease [453–455]. This section will only highlight the correlation between autophagy, apoptosis, and oxidative stress in the following neurodegenerative diseases: Alzheimer's disease, Parkinson's disease, and amyotrophic lateral sclerosis.

### 10.1. Alzheimer's Disease

Alzheimer's disease (AD), the most common form of dementia, is a neurodegenerative disease currently affecting millions of people worldwide. Age is one risk factor of AD, as the onset of disease typically occurs at about 65 years old. Although AD is an age-related neurodegenerative disease, it can be inherited (familial AD) and sporadic. Familial AD (FAD) is very rare, as it affects less than 10% of those afflicted with the disease. There are four distinct types of FAD caused by the genetic factors described below [456–458]. Sporadic AD usually occurs in the late stage of AD and is associated with apolipoprotein E4 (APOE4) allele. Apolipoprotein E (APOE) helps transport cholesterol into the bloodstream. APOE4 is also involved in learning and memory as persons with the APOE4 allele showed a decline in processing new information [459]. APOE4 shows an increase of amyloid beta peptide and neurofibrillary tangles (NFT) [460, 461]. Increased oxidative stress is prevalent in AD and also linked to APOE4 [462, 463]. Ramassamy et al. demonstrated that tissue from patients with the APOE4 allele showed a decrease in activity of the antioxidant enzymes glutathione peroxidase and catalase [463]. Other risk factors include family history of disease, reduced brain volume, traumatic brain injury, and low education and mental ability early in life [464–468].

Extreme neurofibrillary tangles and senile plaques are the hallmarks of AD. These NFT are composed of paired helical filaments, which consist of hyperphosphorylated tau protein. The senile plaques are composed of amyloid beta (A*β*) peptide. Amyloid precursor protein (APP) is a transmembrane protein that plays a role in neuronal plasticity, long term potentiation, and memory loss [469]. APP is proteolytically cleaved by the enzymes *β*-secretase and *γ*-secretase to form a 40–42 amino acids' peptide, amyloid beta (A*β*) peptide. The two major forms of A*β* found in human brain are A*β*(1–40) and A*β*(1–42). A*β*(1–42) is the more toxic form of A*β* and is the primary component of the senile plaque [470, 471]. A*β*(1–42) induces oxidative stress* in vivo* [472, 473] and* in vitro* [474]. After this toxic peptide is formed, it can aggregate and these aggregates can accumulate outside or inside the cell contributing to the pathogenesis of AD.

There are three human causative genetic alterations in AD (*APP*;* PS1*; and* PS2*) and several genes associated with AD (APOE4, *α*-2 macroglobulin, chromosome 10, and chromosome 12). APP is a precursor to amyloid beta peptide. There are several APP mutations (i.e., Artic, Flemish London, Dutch, Italian, Indiana, Iranian, etc.) that cause AD by many different mechanisms (i.e., protofibril formation, dense senile plaques, and increased A*β*(1–42) production) [457, 475–478]. The highly characterized Swedish mutant Tg2576 transgenic mouse model for Alzheimer's disease contains human APP and has A*β* plaques deposits as early as 9 months old [479, 480], leading to increased memory decline with age. Presenilin 1 (PS1) and presenilin 2 (PS2) are catalytic components of *γ*-secretase and are highly involved in APP processing. Mutations in PS1 or PS2 show an increase in A*β*(1–42) production and are the cause of most FAD cases [456, 481–484]. Mutations in APP, PS1, and PS2 have been found in the first clinical stage of AD and MCI [485], early stage AD (EAD) [486], and late stage AD [487]. The APP/PS1 mutant mouse is a common model for AD as it exhibits early amyloid deposition and increased oxidative stress [488, 489]. A significant increase in levels of protein carbonyls, 4-hydroxynonenal, and 3-nitrotyrosine levels, markers of oxidative stress, was exhibited in APP/PS1 double mutant neurons compared to wild type [489]. A triple transgenic (3XTg-AD) mouse model has been recently used to study the pathogenesis of Alzheimer disease. Similarly to the double APP/PS1 mouse, the 3XTg-AD mouse model exhibits oxidative imbalance as antioxidant levels are reduced and lipid peroxidation and overall brain oxidation are increased [490].

These mice have mutations in the PS1 gene and are homozygous for the Swedish APP mutation and tau P301L mutation, making them highly representative of this disorder and an excellent model to use as amyloid deposition is observed at 3 months, hippocampal hyperphosphorylated tau appears at 12–15 months, and synaptic transmission is impaired at an early age, making them ideal to study this neurodegenerative disorder.

Chromosome 10 codes several particular genes of interest as possible risk factors of AD [491]. Insulin degrading enzyme (IDE) is one such gene. IDE degrades and clears A*β* in the brain [492, 493]. IDE levels are reduced in hippocampus [494] and this protein's catalytic activity is lowered in AD as well [495]. IDE, as the name infers, degrades insulin. Statin drugs have been shown to promote astrocytic IDE secretion in AD model to stimulate autophagy [496]. Abnormalities in insulin metabolism are associated with APOE status. AD patients with the APOE4 allele had higher insulin levels than patients without E4 allele [497] Elevated plasma insulin levels correlate to increased A*β* levels, which as previously mentioned has detrimental effects [498, 499]. Therefore, chromosome 10 mutations may be a possible risk factor in AD.

*α*-2-Macroglobulin (*α*_2_M) is encoded by chromosome 12 and is thought to be a possible risk factor for AD as well [500]. *α*_2_M protein binds to A*β* and transports it from neurons into cells for degradation using the LRP receptor [501]. APOE uses this same receptor to enter into cells. Therefore, APOE4 or excess APOE may prevent the A*β*/*α*_2_M complex from binding to the receptor and clearing for the neuron. *α*_2_M has been shown to be neuroprotective against *β* amyloid fibrils* in vitro* [502] and* in vivo* [503] inhibiting toxicity. Mutations in this protein may result in A*β* deposition and neuron death [504].

Microglial activation has been linked to A*β* plaques in Alzheimer's disease. This activation results in a neuroinflammation and phagocytic impairment [505]. This inflammatory response leads to the release of TNF-*α* and interleukin-1*β* [506]. Once the A*β* peptide activates the microglia, nitric oxide is released [413], thereby promoting an oxidative stress via peroxynitrite elevation which can alter calcium homeostasis and promote cellular apoptosis [507].

### 10.2. Parkinson's Disease

Parkinson disease (PD), the second most prevalent age-dependent neurodegenerative disorder, is classified as a tauopathy, a neurological disorder that exhibits excessive levels of phosphorylated tau.

Tau is a microtubule-associated protein that is responsible for stabilizing microtubules. Microtubules are neuron-resident, cylindrically shaped, dynamic structures composed of alternating rows of *α*- and *β*-tubulin. Microtubules play a pivotal role in facilitating intracellular transport by assisting in motor protein-driven transport of vesicles, mitochondria, and other cargos in neurons. If tau is damaged, it can no longer stabilize these microtubules, thereby reducing transport of critical factors to the neuron due to cytoskeletal disintegration. Ultimately, neurons become energy starved leading to apoptosis. Parkinson's disease is strongly correlated with neuroinflammation which is demonstrated by activated astrocytes and microglia in the central nervous system. The activation of these glial cells is detrimental to neurons and promotes neuronal loss.

Parkinson's disease presents as a decline in motor function in the form of resting tremors, muscle rigidity, and dyskinesia. This disease is attributed to protein aggregates of *α*-synuclein, a protein whose main function is mitochondrial functionality, vesicle trafficking, and synaptic vesicle formation [508, 509]. Alpha synuclein is predominantly located in the presynaptic terminals; thereby accumulation results in poor neurotransmission. These aggregates are the major component of Lewy bodies located primarily in the putamen and substantia nigra of the brain. These two regions are largely involved in motor movement and learning. Activated microglia are found largely in the substantia nigra and striatum of PD animals [510]. Oxidative stress in PD brain is evident by DNA damage [511] and increased levels of carbonylated proteins [512]. Neuronal dopamine, a key neurotransmitter involved in motor function, levels are significantly diminished in the substantia nigra causing substantial neuronal death. Aggregation also leads to Complex I impairment in dopaminergic neurons [513]. Protein aggregation in combination with dopamine loss causes a profound effect on the physical capabilities of persons suffering from Parkinson disease and late in the disease cognitive dysfunction is often observed. Posttranslational nitration of alpha synuclein [514] and Complex I [508] lead to altered energy metabolism which is evident in neurodegenerative disorders that are associated with oxidative stress.

The most commonly used models of PD include treatment with rotenone, 6-OHDA, or 1-methyl-4-phenyl-1,2,5,6-tetrahydropyridine (MPTP) induction. As muscle rigidity is a hallmark of PD, mitochondria are highly impacted by this neurodegenerative disease. Both the Krebs cycle and oxidative phosphorylation are the major energy generating processes of the mitochondria. When combined, both processes yield approximately 40 ATP molecules, providing the necessary energy for the body's daily function. Rotenone is an inhibitor to Complex I in the mitochondria, thereby reducing the proton gradient and energy production of the cell. Neurons treated with as little as 0.3 *μ*M of rotenone generated superoxide radicals, thereby promoting oxidative stress [515].

1-Methyl-4-phenyl-4-propionoxy-piperidine (MPPP) and 1-methyl-4-phenyl-1,2,5,6-tetrahydropyridine (MPTP) have been shown to induce PD pathology [516]. Originally, they were discovered in heroin patients who developed Parkinsonian-like symptoms following use of a “synthetic heroin” distributed in California in the 1980s. This synthetic blend was a mixture of MPTP and MPPP. MPTP easily crosses the blood-brain barrier and is taken up by astrocytes and metabolized into 1-methyl-4-phenylpyridinium (MPP^+^), which further breaks down into toxic compounds including 3,4-dihydroxyphenylacetaldehyde (DOPAL) [517], a compound that removes dopamine from synaptic vesicles. The oxidized product, MPP^+^, inhibits Complex I of the electron transport chain working in a similar mechanism as rotenone. MPTP induces a slow progression of the nonmotor and motor symptoms of PD, allowing for longitudinal studies that demonstrate a realistic progression of PD. This is preferred by PD researchers over other toxins such as 6-hydroxydopamine (6-OHDA) [517].

Recent research has investigated the use of gliptins to prevent neuronal loss in animal models of Parkinson's disease. Gliptins are peptides that prevent neurodegeneration by preventing apoptosis and neuroinflammation by altering GLP-1, a glucagon-like peptide-1. Abdelsalam has shown that vildagliptin, a dipeptidyl peptidase, reduced iNOS, caspase-3, and myeloperoxidase levels. The antioxidant potential of this drug also blocked the RAGE/NFK-*β* cascade, thereby lessening neuroinflammation in the rodent rotenone Parkinsonian model [518]. Additionally, activation of aldehyde dehydrogenase, a mitochondrial enzyme responsible for detoxification of toxic aldehydes, has been shown to protect SH-SY5Y cells against rotenone-induced apoptotic cell death, thereby lowering oxidative stress levels [519]. Other studies involving the upregulation of antioxidants to promote cell survival in rotenone cell mediated toxicity and MPTP-induced Parkinson disease include ferulic acid (Ojha, DDT, 2015), catalase [520], a sirtuin, SIRT5 [521], pyrroloquinoline quinone [522], and the phenylpropanoid glycoside salidroside [523]. These results are promising in the development of a potential Parkinson's disease strategy.

The neurotoxin 6-hydroxydopamine (6-OHDA) is also capable of inducing Parkinson's-like symptoms, and its use in animal models of PD predates the discovery of MPTP's neurotoxic properties [524]. This destroys tyrosine hydroxylase (TH) containing neurons [517]. TH catalyzes the rate-limiting step for the production of dopamine, a pivotal neurotransmitter involved in motor functions. This neurotoxin plays a key role in modifying the nigrostriatal pathway in which dopaminergic neurons are produced.

Recently, Kostrzewa et al. have shown that early administration of 6-OHPA promotes a lifelong model for severe Parkinson's disease [525]. In this study, bilateral intracisternal or intracerebroventricular administration of 6-hydroxydopamine (6-OHDA) was given to perinatal rats. Elevated levels of hydroxyl radical and extreme loss of striatal dopaminergic neurons were observed. However, there were no differences in lifespan, feeding behavior, or motor function. This novel model promotes lifelong Parkinsonian symptoms without the characteristic motor deficits observed in PD, providing a truly unique system to study Parkinson's disease progression. Other studies aimed at reducing the oxidative stress promoted by 6-OHDA induction include the administration of antioxidants that also have anti-inflammatory properties such as echinacoside [526], PEG conjugated recombinant human FGF-2 [527], madopar (a combination of the dopamine precursor, levodopa, and benserazide, a decarboxylase inhibitor) [528], guanosine, an MPP^+^ antagonist [529], and carnosine [530]. A safe, effective, combinatorial therapy of GAD and AAV2 vector which codes for the dopamine synthetic enzyme, aromatic-l-amino decarboxylase (AADC), has yielded encouraging results in a small number of PD patients by improving motor performance [531, 532]. This drug is currently in Phase II clinical trial testing. These treatment strategies bolster the theory of oxidative stress as a contributor to neurodegeneration observed in neurodegenerative diseases.

### 10.3. Amyotrophic Lateral Sclerosis

Amyotrophic lateral sclerosis (ALS), commonly known as Lou Gehrig's disease, is a progressive neurodegenerative disorder in which all voluntary muscle movement is lost within 1–5 years after disease diagnosis. This disorder rapidly progresses to death within 2–5 years after the first symptoms are observed. ALS has two forms: sporadic or inherited (familial), with approximately 90% of all ALS cases classified as sporadic. Like PD, motor neurons are greatly affected in ALS resulting in muscle weakness, spasticity, and atrophy [533]. In the motor cortex, cerebellum, and parietal cortex, both forms of ALS display protein oxidation, DNA damage, and MDA modified proteins. Patients suffering from sporadic ALS showed a significant increase in these aforementioned oxidative insults compared to familial ALS subjects, thereby supporting the role of oxidative stress in amyotrophic lateral sclerosis [452]. Twenty percent of persons with inherited ALS have a mutation in the antioxidant enzyme superoxide dismutase, which leads to cellular toxicity [534]. This mutation lowers the ability to combat potentially harmful free radicals, thereby increasing levels of oxidative stress [535]. Mutant SOD1 can aggregate in the cytoplasm in motor neurons of inherited ALS patients and in various mouse models. These aggregates are capable of inducing apoptosis in cortical neurons of the G93A-SOD1 mouse, which has been widely studied as it has pathology similar to that of ALS. In the G93A-SOD1 mouse model, Ala for Gly substitution occurs at position 93. This transgenic mouse overexpresses mutant human SOD1 and exhibits the age-dependent motor neuronal characteristics associated with amyotrophic lateral sclerosis. In familial ALS patients with SOD1 mutations, a significant increase in oxidative stress, as indexed by protein carbonyls, was observed compared to control subjects [536].

It has been speculated that autophagy may play a direct role in neurodegeneration via this glutamate mechanism. This prevents the accumulation of proteins thereby reducing the risk of several neurodegenerative disorders including Alzheimer's disease (accumulation of A*β*), Parkinson's disease (accumulation of alpha synuclein), and Huntington's disease (accumulation of the huntingtin protein). Interestingly, Manchon has shown that the huntingtin protein is degraded by sphingosine kinase 1 in the sphingosine-1-phosphate pathway to promote cell survival via autophagy [537]. These results show a novel target for treatment of neurodegenerative disease. Defects in this process have been strongly associated with neurodegeneration [538, 539].

As ALS is a motor neuron disorder, the motor neurons of the central nervous system are greatly affected. Rojas et al. have shown that, in astrocytes of conditioned media that express human SOD1 in the G93A mouse model, c-Abl was activated [540]. This activation also caused opening of the mitochondrial permeability transition pore. C-Abl is a tyrosine kinase that promotes apoptosis and ROS generation. Administration of the antioxidants trolox, esculetin, and tiron prevented c-Abl activation, thereby reducing oxidative stress and neuronal loss. In cell culture, it has been demonstrated that cells from the spinal cords of G93A mice overexpress transcription factor EB, which regulates autophagy. The expression of beclin-1 and LC3-II, both crucial autophagic markers, was observed as well in this study [541].

### 10.4. Mitochondrial Effect

Mitochondria are the powerhouse of ATP production in the cell. Mitochondria travel on microtubules with assistance of motor proteins (kinesin and dynein) to the presynaptic terminal and return to the soma. Mitochondrial dysfunction is a classic event observed in neurodegeneration as increased oxidative stress has deleterious effects on the mitochondria. Reduced energy metabolism has been observed in most neurodegenerative disorders. Dysregulation of mitochondria may also lead to alterations in the mitochondrial membrane potential which is an early characteristic of apoptosis. One leading theory to prevent neurodegeneration is reversing mitochondrial dysfunction. Several antioxidant therapies have been strategically targeted to the mitochondria to support this notion. Coenzyme Q_10_ (CoQ_10_) plays a key role in oxidative phosphorylation and has neuroprotective properties. It has been widely investigated in the treatment of neurodegenerative disorders [542, 543]. This quinone delays functional decline but does not increase dopamine levels. Although coenzyme Q_10_ has shown promising results, its transport across the blood-brain barrier is poor. A new family of mitochondrial antioxidants, the Szeto-Schiller (SS) peptides, have shown an increase in motor function and cell survival [544]. These antioxidants have also protected dopaminergic neurons against MPTP neurotoxicity, thereby reducing effects of Parkinson's disease and stimulating the field of Parkinson's disease research. Another avenue of treatment includes stimulation of the high mobility group box 1 protein (HMGB-1). The high mobility group box 1 protein is a chromatin binding protein that recognizes DNA damage and promotes binding to p53 to stimulate an oxidative stress response, namely, autophagy or apoptosis. Studies investigating the correlation of HMGB-1 and mitochondrial dysfunction in 3-nitropropionic acid treated animals have been conducted to study the role HGMB-1 may have on striatal neurodegeneration* in vivo* and* in vitro* [545]. They demonstrated that HMGB-1 binds to beclin-1 to regulate autophagy, thereby establishing a new mechanism to study in striatal neurodegeneration via autophagy and apoptosis.

Oxidative stress is a well-established phenomenon that occurs in neurodegenerative disease. This coupled with an increase in apoptosis and autophagy contributes to the neurodegeneration and memory loss observed in Alzheimer's disease, Parkinson's disease, and amyotrophic lateral sclerosis. Reactive oxygen and nitrogen species are highly abundant in these disorders as well. New antioxidant and mitochondrial based therapies show promise to reduce neuronal cell loss and promote neuroprotection, which will have a positive effect on patient outcomes.

## 11. Conclusions

In the whole, available data indicate that mitochondria are a significant source of ROS, but evidence for or against mitochondria being the main source of cellular ROS is lacking. However, there is convincing evidence that increases in mitochondrial ROS production can lead to mitochondrial and cellular oxidative stress and dysfunction, even though the same ROS can trigger mechanisms of tissue protection against excessive oxidative stress. Less information is available on role played by other cellular ROS sources in these processes. However, accumulating evidence favors the idea that, in many cells and conditions, such sources play a role in cellular oxidative damage as well as in survival mechanisms activated by oxidative stress, contributing to tissue rescue from excessive damage and dysfunction.

## Figures and Tables

**Table 1:** Main reactive oxygen (ROS) and nitrogen (RNS) species.

Radicals	O_2_^•−^	Superoxide anion
	^•^OH	Hydroxyl
	NO^•^	Nitric oxide
	NO_2_^•^	Nitrogen dioxide

Nonradicals	H_2_O_2_	Hydrogen peroxide
	HOCl	Hypochlorous acid
	ONOO^−^	Peroxynitrite

## References

[1] Gomberg M. (1900). An instance of trivalent carbon: triphenylmethyl. *Journal of the American Chemical Society*.

[2] Commoner B., Townsend J., Pake G. E. (1954). Free radicals in biological materials. *Nature*.

[3] Gerschman R., Gilbert D. L., Nye S. W., Dwyer P., Fenn W. O. (1954). Oxygen poisoning and X-irradiation: a mechanism in common. *Science*.

[4] Harman D. (1956). Aging: a theory based on free radical and radiation chemistry. *Journal of Gerontology*.

[5] McCord J. M., Fridovich I. (1969). Superoxide dismutase. An enzymic function for erythrocuprein (hemocuprein). *The Journal of Biological Chemistry*.

[6] Bartosz G. (2009). Reactive oxygen species: destroyers or messengers?. *Biochemical Pharmacology*.

[7] Halliwell B. (1987). Oxidants and human disease: some new concepts. *The FASEB Journal*.

[8] Radi R. (2013). Peroxynitrite, a stealthy biological oxidant. *The Journal of Biological Chemistry*.

[9] Yu B. P. (1994). Cellular defenses against damage from reactive oxygen species. *Physiological Reviews*.

[10] Sies H. (1991). *Oxidative Stress, Oxidants and Antioxidants*.

[11] Valko M., Leibfritz D., Moncol J., Cronin M. T. D., Mazur M., Telser J. (2007). Free radicals and antioxidants in normal physiological functions and human disease. *International Journal of Biochemistry & Cell Biology*.

[12] Aboul-Enein H. Y., Berczyński P., Kruk I. (2013). Phenolic compounds: the role of redox regulation in neurodegenerative disease and cancer. *Mini-Reviews in Medicinal Chemistry*.

[13] Mittal C. K., Murad F. (1977). Activation of guanylate cyclase by superoxide dismutase and hydroxyl radical: a physiological regulator of guanosine 3′,5′-monophosphate formation. *Proceedings of the National Academy of Sciences of the United States of America*.

[14] Brieger K., Schiavone S., Miller F. J., Krause K.-H. (2012). Reactive oxygen species: from health to disease. *Swiss Medical Weekly*.

[15] Harman D. (2009). Origin and evolution of the free radical theory of aging: a brief personal history, 1954–2009. *Biogerontology*.

[16] Ristow M., Schmeisser S. (2011). Extending life span by increasing oxidative stress. *Free Radical Biology and Medicine*.

[17] Ignarro L. J., Buga G. M., Wood K. S., Byrns R. E., Chaudhuri G. (1987). Endothelium-derived relaxing factor produced and released from artery and vein is nitric oxide. *Proceedings of the National Academy of Sciences of the United States of America*.

[18] Bogdan C. (2001). Nitric oxide and the regulation of gene expression. *Trends in Cell Biology*.

[19] Pacher P., Beckman J. S., Liaudet L. (2007). Nitric oxide and peroxynitrite in health and disease. *Physiological Reviews*.

[20] Finkel T., Holbrook N. J. (2000). Oxidants, oxidative stress and the biology of ageing. *Nature*.

[21] Lambert A. J., Brand M. D. (2009). Reactive oxygen species production by mitochondria. *Methods in Molecular Biology*.

[22] Dai D.-F., Chiao Y. A., Marcinek D. J., Szeto H. H., Rabinovitch P. S. (2014). Mitochondrial oxidative stress in aging and healthspan. *Longevity & Healthspan*.

[23] Brown G. C., Borutaite V. (2012). There is no evidence that mitochondria are the main source of reactive oxygen species in mammalian cells. *Mitochondrion*.

[24] Camões F., Bonekamp N. A., Delille H. K., Schrader M. (2009). Organelle dynamics and dysfunction: a closer link between peroxisomes and mitochondria. *Journal of Inherited Metabolic Disease*.

[25] Vannuvel K., Renard P., Raes M., Arnould T. (2013). Functional and morphological impact of ER stress on mitochondria. *Journal of Cellular Physiology*.

[26] Freeman B. A., Crapo J. D. (1982). Biology of disease: free radicals and tissue injury. *Laboratory Investigation*.

[27] McCord J. M., Roy R. S., Schaffer S. W. (1985). Free radicals and myocardial ischemia. The role of xanthine oxidase. *Advances in Myocardiology*.

[28] Navarro A., Boveris A. (2007). The mitochondrial energy transduction system and the aging process. *American Journal of Physiology—Cell Physiology*.

[29] Mitchell P., Moyle J. (1965). Stoichiometry of proton translocation through the respiratory chain and adenosine triphosphatase systems of rat liver mitochondria. *Nature*.

[30] Walker J. E., Collinson I. R., Van Raaij M. J., Runswick M. J. (1995). Structural analysis of ATP synthase from bovine heart mitochondria. *Methods in Enzymology*.

[31] Jensen P. K. (1966). Antimycin-insensitive oxidation of succinate and reduced nicotinamide-adenine dinucleotide in electron-transport particles. I. pH dependency and hydrogen peroxide formation. *Biochimica et Biophysica Acta (BBA)—Enzymology and Biological Oxidation*.

[32] Loschen G., Azzi A., Richter C., Flohé L. (1974). Superoxide radicals as precursors of mitochondrial hydrogen peroxide. *FEBS Letters*.

[33] Turrens J. F., Boveris A. (1980). Generation of superoxide anion by the NADH dehydrogenase of bovine heart mitochondria. *Biochemical Journal*.

[34] Quinlan C. L., Perevoshchikova I. V., Hey-Mogensen M., Orr A. L., Brand M. D. (2013). Sites of reactive oxygen species generation by mitochondria oxidizing different substrates. *Redox Biology*.

[35] Orr A. L., Quinlan C. L., Perevoshchikova I. V., Brand M. D. (2012). A refined analysis of superoxide production by mitochondrial sn-glycerol 3-phosphate dehydrogenase. *The Journal of Biological Chemistry*.

[36] Brand M. D. (2010). The sites and topology of mitochondrial superoxide production. *Experimental Gerontology*.

[37] Kwong L. K., Sohal R. S. (1998). Substrate and site specificity of hydrogen peroxide generation in mouse mitochondria. *Archives of Biochemistry and Biophysics*.

[38] Boveris A., Oshino N., Chance B. (1972). The cellular production of hydrogen peroxide. *Biochemical Journal*.

[39] St-Pierre J., Buckingham J. A., Roebuck S. J., Brand M. D. (2002). Topology of superoxide production from different sites in the mitochondrial electron transport chain. *Journal of Biological Chemistry*.

[40] Andreyev A. Y., Kushnareva Y. E., Starkov A. A. (2005). Mitochondrial metabolism of reactive oxygen species. *Biochemistry*.

[41] Hauptmann N., Grimsby J., Shih J. C., Cadenas E. (1996). The metabolism of tyramine by monoamine oxidase A/B causes oxidative damage to mitochondrial DNA. *Archives of Biochemistry and Biophysics*.

[42] Löffler M., Becker C., Wegerle E., Schuster G. (1996). Catalytic enzyme histochemistry and biochemical analysis of dihydroorotate dehydrogenase/oxidase and succinate dehydrogenase in mammalian tissues, cells and mitochondria. *Histochemistry and Cell Biology*.

[43] Zhang L., Yu L., Yu C.-A. (1998). Generation of superoxide anion by succinate-cytochrome c reductase from bovine heart mitochondria. *The Journal of Biological Chemistry*.

[44] Starkov A. A., Fiskum G., Chinopoulos C. (2004). Mitochondrial *α*-ketoglutarate dehydrogenase complex generates reactive oxygen species. *The Journal of Neuroscience*.

[45] De Duve C., Baudhuin P. (1966). Peroxisomes (microbodies and related particles). *Physiological Reviews*.

[46] Fransen M. (2012). Peroxisome dynamics: molecular players, mechanisms, and (Dys)functions. *ISRN Cell Biology*.

[47] Singh I. (1997). Biochemistry of peroxisomes in health and disease. *Molecular and Cellular Biochemistry*.

[48] Antonenkov V. D., Grunau S., Ohlmeier S., Hiltunen J. K. (2010). Peroxisomes are oxidative organelles. *Antioxidants and Redox Signaling*.

[49] Rokka A., Antonenkov V. D., Soininen R. (2009). Pxmp2 is a channel-forming protein in mammalian peroxisomal membrane. *PLoS ONE*.

[50] Fritz R., Bol J., Hebling U. (2007). Compartment-dependent management of H_2_O_2_ by peroxisomes. *Free Radical Biology and Medicine*.

[51] Angermüller S., Bruder G., Völkl A., Wesch H., Fahimi H. D. (1987). Localization of xanthine oxidase in crystalline cores of peroxisomes. A cytochemical and biochemical study. *European Journal of Cell Biology*.

[52] Stolz D. B., Zamora R., Vodovotz Y. (2002). Peroxisomal localization of inducible nitric oxide synthase in hepatocytes. *Hepatology*.

[53] Groenendyk J., Michalak M. (2005). Endoplasmic reticulum quality control and apoptosis. *Acta Biochimica Polonica*.

[54] Koch G. L. E. (1990). The endoplasmic reticulum and calcium storage. *BioEssays*.

[55] Crib A. E., Peyrou M., Muruganandan S., Schneider L. (2005). The endoplasmic reticulum in xenobiotic toxicity. *Drug Metabolism Reviews*.

[56] Gillette J. R., Brodie B. B., La Du B. N. (1957). The oxidation of drugs by liver microsomes: on the role of TPNH and oxygen. *The Journal of Pharmacology and Experimental Therapeutics*.

[57] Estabrook R. W., Kawano S., Werringloer J. (1979). Oxycytochrome P-450: its breakdown to superoxide for the formation of hydrogen peroxide. *Acta Biologica et Medica Germanica*.

[58] Kuthan H., Ullrich V. (1982). Oxidase and oxygenase function of the microsomal cytochrome P450 monooxygenase system. *European Journal of Biochemistry*.

[59] Stadtman E. R. (1986). Oxidation of proteins by mixed-function oxidation systems: implication in protein turnover, ageing and neutrophil function. *Trends in Biochemical Sciences*.

[60] Aguilar P. S., de Mendoza D. (2006). Control of fatty acid desaturation: a mechanism conserved from bacteria to humans. *Molecular Microbiology*.

[61] Thiede M. A., Ozols J., Strittmatter P. (1986). Construction and sequence of cDNA for rat liver stearyl coenzyme A desaturase. *The Journal of Biological Chemistry*.

[62] Napier J. A., Michaelson L. V., Sayanova O. (2003). The role of cytochrome b5 fusion desaturases in the synthesis of polyunsaturated fatty acids. *Prostaglandins Leukotrienes and Essential Fatty Acids*.

[63] Mitchell A. G., Martin C. E. (1995). A novel cytochrome b5-like domain is linked to the carboxyl terminus of the *Saccharomyces cerevisiae*Δ-9 fatty acid desaturase. *The Journal of Biological Chemistry*.

[64] Schenkman J. B., Jansson I. (2003). The many roles of cytochrome b5. *Pharmacology & Therapeutics*.

[65] Samhan-Arias A. K., Gutierrez-Merino C. (2014). Purified NADH-cytochrome b5 reductase is a novel superoxide anion source inhibited by apocynin: sensitivity to nitric oxide and peroxynitrite. *Free Radical Biology and Medicine*.

[66] Hwang C., Sinskey A. J., Lodish H. F. (1992). Oxidized redox state of glutathione in the endoplasmic reticulum. *Science*.

[67] Frand A. R., Kaiser C. A. (1999). Ero1p oxidizes protein disulfide isomerase in a pathway for disulfide bond formation in the endoplasmic reticulum. *Molecular Cell*.

[68] Tu B. P., Weissman J. S. (2002). The FAD- and O_2_-dependent reaction cycle of Ero1-mediated oxidative protein folding in the endoplasmic reticulum. *Molecular Cell*.

[69] Tu B. P., Weissman J. S. (2004). Oxidative protein folding in eukaryotes: mechanisms and consequences. *The Journal of Cell Biology*.

[70] Skulachev V. P. (1996). Why are mitochondria involved in apoptosis? Permeability transition pores and apoptosis as selective mechanisms to eliminate superoxide-producing mitochondria and cell. *FEBS Letters*.

[71] Cho K.-J., Seo J.-M., Kim J.-H. (2011). Bioactive lipoxygenase metabolites stimulation of NADPH oxidases and reactive oxygen species. *Molecules and Cells*.

[72] Babior B. M. (1999). NADPH oxidase: an update. *Blood*.

[73] Chanock S. J., El Benna J., Smith R. M., Babior B. M. (1994). The respiratory burst oxidase. *The Journal of Biological Chemistry*.

[74] Suh Y.-A., Arnold R. S., Lassegue B. (1999). Cell transformation by the superoxide-generating oxidase Mox1. *Nature*.

[75] Katsuyama M. (2010). NOX/NADPH oxidase, the superoxide-generating enzyme: its transcriptional regulation and physiological roles. *Journal of Pharmacological Sciences*.

[76] Kim C., Dinauer M. C. (2006). Impaired NADPH oxidase activity in Rac2-deficient murine neutrophils does not result from defective translocation of p47phox and p67 phox and can be rescued by exogenous arachidonic acid. *The Journal of Leukocyte Biology*.

[77] Mahipal S. V. K., Subhashini J., Reddy M. C. (2007). Effect of 15-lipoxygenase metabolites, 15-(*S*)-HPETE and 15-(*S*)-HETE on chronic myelogenous leukemia cell line K-562: reactive oxygen species (ROS) mediate caspase-dependent apoptosis. *Biochemical Pharmacology*.

[78] Hong H.-Y., Jeon W.-K., Kim B.-C. (2008). Up-regulation of heme oxygenase-1 expression through the Rac1/NADPH oxidase/ROS/p38 signaling cascade mediates the anti-inflammatory effect of 15-deoxy-Δ12,14-prostaglandin J2 in murine macrophages. *FEBS Letters*.

[79] Sun-Wada G.-H., Wada Y., Futai M. (2003). Lysosome and lysosome-related organelles responsible for specialized functions in higher organisms, with special emphasis on vacuolar-type proton ATPase. *Cell Structure and Function*.

[80] Arai K., Kanaseki T., Ohkuma S. (1991). Isolation of highly purified lysosomes from rat liver: identification of electron carrier components on lysosomal membranes. *Journal of Biochemistry*.

[81] Gille L., Nohl H. (2000). The existence of a lysosomal redox chain and the role of ubiquinone. *Archives of Biochemistry and Biophysics*.

[82] Nohl H., Gille L. (2002). The bifunctional activity of ubiquinone in lysosomal membranes. *Biogerontology*.

[83] Mayer B., Hemmens B. (1997). Biosynthesis and action of nitric oxide in mammalian cells. *Trends in Biochemical Sciences*.

[84] Nathan C., Xie Q.-W. (1994). Nitric oxide synthases: roles, tolls, and controls. *Cell*.

[85] Feron O., Belhassen L., Kobzik L., Smith T. W., Kelly R. A., Michel T. (1996). Endothelial nitric oxide synthase targeting to caveolae. Specific interactions with caveolin isoforms in cardiac myocytes and endothelial cells. *The Journal of Biological Chemistry*.

[86] Sánchez F. A., Savalia N. B., Durán R. G., Lal B. K., Boric M. P., Durán W. N. (2006). Functional significance of differential eNOS translocation. *American Journal of Physiology-Heart and Circulatory Physiology*.

[87] Loughran P. A., Stolz D. B., Vodovotz Y., Watkins S. C., Simmons R. L., Billiar T. R. (2005). Monomeric inducible nitric oxide synthase localizes to peroxisomes in hepatocytes. *Proceedings of the National Academy of Sciences of the United States of America*.

[88] Parihar M. S., Nazarewicz R. R., Kincaid E., Bringold U., Ghafourifar P. (2008). Association of mitochondrial nitric oxide synthase activity with respiratory chain complex I. *Biochemical and Biophysical Research Communications*.

[89] Paolocc N., Ekelund U. E. G., Isoda T. (2000). cGMP-independent inotropic effects of nitric oxide and peroxynitrite donors: potential role for nitrosylation. *American Journal of Physiology—Heart and Circulatory Physiology*.

[90] Cleeter M. W. J., Cooper J. M., Darley-Usmar V. M., Moncada S., Schapira A. H. V. (1994). Reversible inhibition of cytochrome c oxidase, the terminal enzyme of the mitochondrial respiratory chain, by nitric oxide. Implications for neurodegenerative diseases. *FEBS Letters*.

[91] Poderoso J. J., Peralta J. G., Lisdero C. L. (1998). Nitric oxide regulates oxygen uptake and hydrogen peroxide release by the isolated beating rat heart. *American Journal of Physiology—Cell Physiology*.

[92] Simon D. I., Mullins M. E., Jia L., Gaston B., Singel D. J., Stamler J. S. (1996). Polynitrosylated proteins: characterization, bioactivity, and functional consequences. *Proceedings of the National Academy of Sciences of the United States of America*.

[93] Borutaite V., Budriunaite A., Brown G. C. (2000). Reversal of nitric oxide-, peroxynitrite- and S-nitrosothiol-induced inhibition of mitochondrial respiration or complex I activity by light and thiols. *Biochimica et Biophysica Acta—Bioenergetics*.

[94] Radi R., Cassina A., Hodara R., Quijano C., Castro L. (2002). Peroxynitrite reactions and formation in mitochondria. *Free Radical Biology and Medicine*.

[95] Lukyanov K. A., Belousov V. V. (2014). Genetically encoded fluorescent redox sensors. *Biochimica et Biophysica Acta—General Subjects*.

[96] Hearse D. J. (1977). Reperfusion of the ischemic myocardium. *Journal of Molecular and Cellular Cardiology*.

[97] Hess M. L., Manson N. H. (1984). Molecular oxygen: friend and foe. The role of the oxygen free radical system in the calcium paradox, the oxygen paradox and ischemia/reperfusion injury. *Journal of Molecular and Cellular Cardiology*.

[98] McCord J. M. (1985). Oxygen-derived free radicals in postischemic tissue injury. *The New England Journal of Medicine*.

[99] Beckman J. S., Beckman T. W., Chen J., Marshall P. A., Freeman B. A. (1990). Apparent hydroxyl radical production by peroxynitrite: implications for endothelial injury from nitric oxide and superoxide. *Proceedings of the National Academy of Sciences of the United States of America*.

[100] Braunersreuther V., Montecucco F., Asrih M. (2013). Role of NADPH oxidase isoforms NOX1, NOX2 and NOX4 in myocardial ischemia/reperfusion injury. *Journal of Molecular and Cellular Cardiology*.

[101] Otani H., Tanaka H., Inoue T. (1984). In vitro study on contribution of oxidative metabolism of isolated rabbit heart mitochondria to myocardial reperfusion injury. *Circulation Research*.

[102] Ambrosio G., Zweier J. L., Duilio C. (1993). Evidence that mitochondrial respiration is a source of potentially toxic oxygen free radicals in intact rabbit hearts subjected to ischemia and reflow. *The Journal of Biological Chemistry*.

[103] Halestrap A. P., Griffiths E. J., Connern C. P. (1993). Mitochondrial calcium handling and oxidative stress. *Biochemical Society Transactions*.

[104] Kane J. J., Murphy M. L., Bisset J. K., deSoyza N., Doherty J. E., Straub K. D. (1975). Mitochondrial function, oxygen extraction, epicardial S-T segment changes and tritiated digoxin distribution after reperfusion of ischemic myocardium. *The American Journal of Cardiology*.

[105] Venditti P., Masullo P., Di Meo S. (2001). Effects of myocardial ischemia and reperfusion on mitochondrial function and susceptibility to oxidative stress. *Cellular and Molecular Life Sciences*.

[106] Brookes P. S., Digerness S. B., Parks D. A., Darley-Usmar V. (2002). Mitochondrial function in response to cardiac ischemia-reperfusion after oral treatment with quercetin. *Free Radical Biology and Medicine*.

[107] Liu P., Hock C. E., Nagele R., Wong P. Y.-K. (1997). Formation of nitric oxide, superoxide, and peroxynitrite in myocardial ischemia-reperfusion injury in rats. *American Journal of Physiology—Heart and Circulatory Physiology*.

[108] Borutaité V., Brown G. C. (1996). Rapid reduction of nitric oxide by mitochondria, and reversible inhibition of mitochondrial respiration by nitric oxide. *Biochemical Journal*.

[109] Radi R., Rodriguez M., Castro L., Telleri R. (1994). Inhibition of mitochondrial electron transport by peroxynitrite. *Archives of Biochemistry and Biophysics*.

[110] Venditti P., De Rosa R., Cigliano L., Agnisola C., Di Meo S. (2004). Role of nitric oxide in the functional response to ischemia-reperfusion of heart mitochondria from hyperthyroid rats. *Cellular and Molecular Life Sciences*.

[111] Zorov D. B., Filburn C. R., Klotz L.-O., Zweier J. L., Sollott S. J. (2000). Reactive oxygen species (ROS)-induced ROS release: a new phenomenon accompanying induction of the mitochondrial permeability transition in cardiac myocytes. *The Journal of Experimental Medicine*.

[112] Vercesi A. E., Kowaltowski A. J., Grijalba M. T., Meinicke A. R., Castilho R. F. (1997). The role of reactive oxygen species in mitochondrial permeability transition. *Bioscience Reports*.

[113] Garlid K. D., Beavis A. D. (1986). Evidence for the existence of an inner membrane anion channel in mitochondria. *Biochimica et Biophysica Acta (BBA)—Reviews on Bioenergetics*.

[114] McCormack J. G., Halestrap A. P., Denton R. M. (1990). Role of calcium ions in regulation of mammalian intramitochondrial metabolism. *Physiological Reviews*.

[115] Aon M. A., Cortassa S., Marbán E., O'Rourke B. (2003). Synchronized whole cell oscillations in mitochondrial metabolism triggered by a local release of reactive oxygen species in cardiac myocytes. *The Journal of Biological Chemistry*.

[116] Venditti P., Napolitano G., Di Meo S. (2015). Role of mitochondria and other ROS sources in hyperthyroidism-linked oxidative stress. *Immunology, Endocrine & Metabolic Agents in Medicinal Chemistry*.

[117] Moncada S., Erusalimsky J. D. (2002). Does nitric oxide modulate mitochondrial energy generation and apoptosis?. *Nature Reviews Molecular Cell Biology*.

[118] Balakirev M. Yu., Khramtsov V. V., Zimmer G. (1997). Modulation of the mitochondrial permeability transition by nitric oxide. *European Journal of Biochemistry*.

[119] Vieira H., Kroemer G. (2003). Mitochondria as targets of apoptosis regulation by nitric oxide. *IUBMB Life*.

[120] Migliaccio E., Giogio M., Mele S. (1999). The p66(shc) adaptor protein controls oxidative stress response and life span in mammals. *Nature*.

[121] Giorgio M., Migliaccio E., Orsini F. (2005). Electron transfer between cytochrome c and p66Shc generates reactive oxygen species that trigger mitochondrial apoptosis. *Cell*.

[122] Nemoto S., Combs C. A., French S. (2006). The mammalian longevity-associated gene product p66shc regulates mitochondrial metabolism. *The Journal of Biological Chemistry*.

[123] Gertz M., Fischer F., Wolters D., Steegborn C. (2008). Activation of the lifespan regulator p66Shc through reversible disulfide bond formation. *Proceedings of the National Academy of Sciences of the United States of America*.

[124] Frazier A. E., Kiu C., Stojanovski D., Hoogenraad N. J., Ryan M. T. (2006). Mitochondrial morphology and distribution in mammalian cells. *Biological Chemistry*.

[125] Nunnari J., Suomalainen A. (2012). Mitochondria: in sickness and in health. *Cell*.

[126] Wu S., Zhou F., Zhang Z., Xing D. (2011). Mitochondrial oxidative stress causes mitochondrial fragmentation via differential modulation of mitochondrial fission-fusion proteins. *The FEBS Journal*.

[127] Yu T., Sheu S.-S., Robotham J. L., Yoon Y. (2008). Mitochondrial fission mediates high glucose-induced cell death through elevated production of reactive oxygen species. *Cardiovascular Research*.

[128] Handy D. E., Loscalzo J. (2012). Redox regulation of mitochondrial function. *Antioxidants and Redox Signaling*.

[129] Cho D.-H., Nakamura T., Fang J. (2009). S-Nitrosylation of Drp1 mediates *β*-amyloid-related mitochondrial fission and neuronal injury. *Science*.

[130] De Palma C., Falcone S., Pisoni S. (2010). Nitric oxide inhibition of Drp1-mediated mitochondrial fission is critical for myogenic differentiation. *Cell Death & Differentiation*.

[131] Issemann I., Green S. (1990). Activation of a member of the steroid hormone receptor superfamily by peroxisome proliferators. *Nature*.

[132] Rao S. M., Reddy J. K. (1987). Peroxisome proliferation and hepatocarcinogenesis. *Carcinogenesis*.

[133] Reddy J. K., Lalvvai N. D., Farber E. (1983). Carcinogenesis by hepatic peroxisome proliferators: evaluation of the risk of hypolipidemic drugs and industrial plasticizers to humans. *Critical Reviews in Toxicology*.

[134] Gonzalez F. J., Peters J. M., Cattley R. C. (1998). Mechanism of action of the nongenotoxic peroxisome proliferators: role of the peroxisome proliferator-activated receptor *α*. *Journal of the National Cancer Institute*.

[135] Peters J. M., Cheung C., Gonzalez F. J. (2005). Peroxisome proliferator-activated receptor-*α* and liver cancer: where do we stand?. *Journal of Molecular Medicine*.

[136] Wanders R. J. A. (2004). Peroxisomes, lipid metabolism, and peroxisomal disorders. *Molecular Genetics and Metabolism*.

[137] Fransen M., Nordgren M., Wang B., Apanasets O. (2012). Role of peroxisomes in ROS/RNS-metabolism: Implications for human disease. *Biochimica et Biophysica Acta - Molecular Basis of Disease*.

[138] Schrader M., Yoon Y. (2007). Mitochondria and peroxisomes: are the ‘Big Brother’ and the ‘Little Sister’ closer than assumed?. *BioEssays*.

[139] Xu B., Moritz J. T., Epstein P. N. (1999). Overexpression of catalase provides partial protection to transgenic mouse beta cells. *Free Radical Biology and Medicine*.

[140] Mueller S., Weber A., Fritz R. (2002). Sensitive and real-time determination of H_2_O_2_ release from intact peroxisomes. *Biochemical Journal*.

[141] Nordgren M., Fransen M. (2014). Peroxisomal metabolism and oxidative stress. *Biochimie*.

[142] Walton P. A., Pizzitelli M. (2012). Effects of peroxisomal catalase inhibition on mitochondrial function. *Frontiers in Physiology*.

[143] Dirkx R., Vanhorebeek I., Martens K. (2005). Absence of peroxisomes in mouse hepatocytes causes mitochondrial and ER abnormalities. *Hepatology*.

[144] López-Erauskin J., Galino J., Ruiz M. (2013). Impaired mitochondrial oxidative phosphorylation in the peroxisomal disease X-linked adrenoleukodystrophy. *Human Molecular Genetics*.

[145] Bhandary B., Marahatta A., Kim H.-R., Chae H.-J. (2013). An involvement of oxidative stress in endoplasmic reticulum stress and its associated diseases. *International Journal of Molecular Sciences*.

[146] Smith M. H., Ploegh H. L., Weissman J. S. (2011). Road to ruin: targeting proteins for degradation in the endoplasmic reticulum. *Science*.

[147] Tabas I., Ron D. (2011). Integrating the mechanisms of apoptosis induced by endoplasmic reticulum stress. *Nature Cell Biology*.

[148] Malhotra J. D., Kaufman R. J. (2007). Endoplasmic reticulum stress and oxidative stress: a vicious cycle or a double-edged sword?. *Antioxidants & Redox Signaling*.

[149] Haynes C. M., Titus E. A., Cooper A. A. (2004). Degradation of misfolded proteins prevents ER-derived oxidative stress and cell death. *Molecular Cell*.

[150] Yoon H., Kim D., Lee G., Kim K., Kim H., Chae H. (2011). Apoptosis induced by manganese on neuronal SK-N-MC cell line: Endoplasmic Reticulum (ER) stress and mitochondria dysfunction. *Environmental Health and Toxicology*.

[151] Franzini-Armstrong C. (2007). ER-mitochondria communication. How privileged?. *Physiology*.

[152] Conney A. H. (1967). Pharmacological implications of microsomal enzyme induction. *Pharmacological Reviews*.

[153] Venditti P., Daniele C. M., De Leo T., Di Meo S. (1998). Effect of phenobarbital treatment on characteristics determining susceptibility to oxidants of homogenates, mitochondria and microsomes from rat liver. *Cellular Physiology and Biochemistry*.

[154] Hildebrandt A. G., Roots I. (1975). Reduced nicotinamide adenine dinucleotide phosphate (NADPH)-dependent formation and breakdown of hydrogen peroxide during mixed function oxidation reactions in liver microsomes. *Archives of Biochemistry and Biophysics*.

[155] Yu C. A., Gunsalus I. C. (1974). Cytochrome P-450_cam_. II. Interconversion with P-420. *The Journal of Biological Chemistry*.

[156] Levin W., Lu A. Y. H., Jacobson M., Kuntzman R., Poyer J. L., McCay P. B. (1973). Lipid peroxidation and the degradation of cytochrome P-450 heme. *Archives of Biochemistry and Biophysics*.

[157] Karuzina I. I., Archakov A. I. (1994). The oxidative inactivation of cytochrome P450 in monooxygenase reactions. *Free Radical Biology and Medicine*.

[158] Mitchell J. R., Smith C. V., Lauterburg B. H., Hughes H., Corcoran J. B., Horning E. C., Mitchell J. R., Horning M. G. (1984). Reactive metabolites and the pathophysiology of acutelethal injury. *Drug Metabolism and Drug Toxicity*.

[159] Talalay P., Prochaska H. J., Spencer S. R. (1990). Regulation of enzymes that detoxify the electrophilic forms of chemical carcinogens. *Princess Takamatsu symposia*.

[160] Conney A. H. (1982). Induction of microsomal enzymes by foreign chemicals and carcinogenesis by polycyclic aromatic hydrocarbons. *Cancer Research*.

[161] Marí M., Cederbaum A. I. (2001). Induction of catalase, alpha, and microsomal glutathione S-transferase in CYP2E1 overexpressing HepG2 cells and protection against short-term oxidative stress. *Hepatology*.

[162] Kurz T., Terman A., Gustafsson B., Brunk U. T. (2008). Lysosomes in iron metabolism, ageing and apoptosis. *Histochemistry and Cell Biology*.

[163] Persson H. L., Kurz T., Eaton J. W., Brunk U. T. (2005). Radiation-induced cell death: importance of lysosomal destabilization. *Biochemical Journal*.

[164] Scherer E., Streffer C., Trott K. R. (1991). *Radiopathology of Organs and Tissues*.

[165] Berndt C., Kurz T., Selenius M., Fernandes A. P., Edgren M. R., Brunk U. T. (2010). Chelation of lysosomal iron protects against ionizing radiation. *Biochemical Journal*.

[166] Kahles T., Brandes R. P. (2013). Which NADPH oxidase isoform is relevant for ischemic stroke? the case for Nox 2. *Antioxidants & Redox Signaling*.

[167] Braunersreuther V., Jaquet V. (2012). Reactive oxygen species in myocardial reperfusion injury: from physiopathology to therapeutic approaches. *Current Pharmaceutical Biotechnology*.

[168] Lassègue B., San Martín A., Griendling K. K. (2012). Biochemistry, physiology, and pathophysiology of NADPH oxidases in the cardiovascular system. *Circulation Research*.

[169] Braunersreuther V., Montecucco F., Asrih M. (2013). Role of NADPH oxidase isoforms NOX1, NOX2 and NOX4 in myocardial ischemia/reperfusion injury. *Journal of Molecular and Cellular Cardiology*.

[170] Daiber A. (2010). Redox signaling (cross-talk) from and to mitochondria involves mitochondrial pores and reactive oxygen species. *Biochimica et Biophysica Acta (BBA)—Bioenergetics*.

[171] Li W.-G., Miller F. J., Zhang H. J., Spitz D. R., Oberley L. W., Weintraub N. L. (2001). H_2_O_2_-induced O_2_—production by a non-phagocytic NAD(P)H Oxidase Causes Oxidant Injury. *The Journal of Biological Chemistry*.

[172] McNally J. S., Saxena A., Cai H., Dikalov S., Harrison D. G. (2005). Regulation of xanthine oxidoreductase protein expression by hydrogen peroxide and calcium. *Arteriosclerosis, Thrombosis, and Vascular Biology*.

[173] McNally J. S., Davis M. E., Giddens D. P. (2003). Role of xanthine oxidoreductase and NAD(P)H oxidase in endothelial superoxide production in response to oscillatory shear stress. *American Journal of Physiology—Heart and Circulatory Physiology*.

[174] Kaltschmidt B., Sparna T., Kaltschmidt C. (1999). Activation of NF-*κ*B by reactive oxygen intermediates in the nervous system. *Antioxidants & Redox Signaling*.

[175] Shackelford R. E., Kaufmann W. K., Paules R. S. (2000). Oxidative stress and cell cycle checkpoint function. *Free Radical Biology and Medicine*.

[176] Sen C. K. (2001). Cellular thiols and redox-regulated signal transduction. *Current Topics in Cellular Regulation*.

[177] Wolin M. S. (2000). Interactions of oxidants with vascular signaling systems. *Arteriosclerosis, Thrombosis, and Vascular Biology*.

[178] Kuehl F. A., Egan R. W. (1980). Prostaglandins, arachidonic acid, and inflammation. *Science*.

[179] Saugstad O. D. (2001). Update on oxygen radical disease in neonatology. *Current Opinion in Obstetrics and Gynecology*.

[180] Benhar M., Engelberg D., Levitzki A. (2002). ROS, stress-activated kinases and stress signaling in cancer. *EMBO Reports*.

[181] Scandalios J. G. (2005). Oxidative stress: molecular perception and transduction of signals triggering antioxidant gene defenses. *Brazilian Journal of Medical and Biological Research*.

[182] Thannickal V. J., Fanburg B. L. (2000). Reactive oxygen species in cell signaling. *American Journal of Physiology—Lung Cellular and Molecular Physiology*.

[183] Roos G., Messens J. (2011). Protein sulfenic acid formation: from cellular damage to redox regulation. *Free Radical Biology and Medicine*.

[184] Singh S., Vrishni S., Singh B. K., Rahman I., Kakkar P. (2010). Nrf2-ARE stress response mechanism: a control point in oxidative stress-mediated dysfunctions and chronic inflammatory diseases. *Free Radical Research*.

[185] Zhang D. D., Hannink M. (2003). Distinct cysteine residues in Keap1 are required for Keap1-dependent ubiquitination of Nrf2 and for stabilization of Nrf2 by chemopreventive agents and oxidative stress. *Molecular and Cellular Biology*.

[186] Ray P. D., Huang B.-W., Tsuji Y. (2012). Reactive oxygen species (ROS) homeostasis and redox regulation in cellular signaling. *Cellular Signalling*.

[187] Zoratti M., Szabò I. (1995). The mitochondrial permeability transition. *Biochimica et Biophysica Acta (BBA)—Reviews on Biomembranes*.

[188] Skulachev V. P. (1998). Uncoupling: new approaches to an old problem of bioenergetics. *Biochimica et Biophysica Acta—Bioenergetics*.

[189] Kuff E. L., Schneider W. C. (1954). Intracellular distribution of enzymes. XII. Biochemical heterogeneity of mitochondria. *The Journal of Biological Chemistry*.

[190] Venditti P., Costagliola I. R., Di Meo S. (2002). H_2_O_2_ production and response to stress conditions by mitochondrial fractions from rat liver. *Journal of Bioenergetics and Biomembranes*.

[191] Venditti P., Daniele M. C., Masullo P., Di Meo S. (1999). Antioxidant-sensitive triiodothyronine effects on characteristics of rat liver mitochondrial population. *Cellular Physiology and Biochemistry*.

[192] Venditti P., Masullo P., Di Meo S. (1999). Effect of exercise duration on characteristics of mitochondrial population from rat liver. *Archives of Biochemistry and Biophysics*.

[193] Venditti P., De Rosa R., Caldarone G., Di Meo S. (2004). Functional and biochemical characteristics of mitochondrial fractions from rat liver in cold-induced oxidative stress. *Cellular and Molecular Life Sciences*.

[194] Venditti P., Napolitano G., Di Stefano L., Di Meo S. (2011). Effect of vitamin E on characteristics of liver mitochondrial fractions from cold-exposed rats. *Journal of Bioenergetics and Biomembranes*.

[195] Levine B., Klionsky D. J. (2004). Development by self-digestion: molecular mechanisms and biological functions of autophagy. *Developmental Cell*.

[196] Debnath J., Baehrecke E. H., Kroemer G. (2005). Does autophagy contribute to cell death?. *Autophagy*.

[197] Kim I., Rodriguez-Enriquez S., Lemasters J. J. (2007). Selective degradation of mitochondria by mitophagy. *Archives of Biochemistry and Biophysics*.

[198] Kopitz J., Kisen G. Ø., Gordon P. B., Bohley P., Seglen P. O. (1990). Nonselective autophagy of cytosolic enzymes by isolated rat hepatocytes. *The Journal of Cell Biology*.

[199] Dunn W. A., Cregg J. M., Kiel J. A. K. W. (2005). Pexophagy: the selective autophagy of peroxisomes. *Autophagy*.

[200] Bernales S., Schuck S., Walter P. (2007). ER-phagy: selective autophagy of the endoplasmic reticulum. *Autophagy*.

[201] Roberts P., Moshitch-Moshkovitz S., Kvam E., O'Toole E., Winey M., Goldfarb D. S. (2003). Piecemeal microautophagy of nucleus in *Saccharomyces cerevisiae*. *Molecular Biology of the Cell*.

[202] Kim I., Lemasters J. J. (2011). Mitophagy selectively degrades individual damaged mitochondria after photoirradiation. *Antioxidants & Redox Signaling*.

[203] Bormann C., Sahm H. (1978). Degradation of microbodies in relation of activities of alcohol oxidase and catalase in *Candida boidinii*. *Archives of Microbiology*.

[204] Aksam E. B., Koek A., Kiel J. A. K. W., Jourdan S., Veenhuis M., Van Der Klei I. J. (2007). A peroxisomal Lon protease and peroxisome degradation by autophagy play key roles in vitality of *Hansenula polymorpha* cells. *Autophagy*.

[205] Huang J., Lam G. Y., Brumell J. H. (2011). Autophagy signaling through reactive oxygen species. *Antioxidants & Redox Signaling*.

[206] Scherz-Shouval R., Elazar Z. (2007). ROS, mitochondria and the regulation of autophagy. *Trends in Cell Biology*.

[207] Scherz-Shouval R., Shvets E., Fass E., Shorer H., Gil L., Elazar Z. (2007). Reactive oxygen species are essential for autophagy and specifically regulate the activity of Atg4. *The EMBO Journal*.

[208] Johansson A.-C., Steen H., Öllinger K., Roberg K. (2003). Cathepsin D mediates cytochrome c release and caspase activation in human fibroblast apoptosis induced by staurosporine. *Cell Death and Differentiation*.

[209] Galluzzi L., Vicencio J. M., Kepp O., Tasdemir E., Maiuri M. C., Kroemer G. (2008). To die or not to die: that is the autophagic question. *Current Molecular Medicine*.

[210] Shimizu S., Kanaseki T., Mizushima N. (2004). Role of Bcl-2 family proteins in a non-apoptotic programmed cell death dependent on autophagy genes. *Nature Cell Biology*.

[211] Gómez-Santos C., Ferrer I., Santidrián A. F., Barrachina M., Gil J., Ambrosio S. (2003). Dopamine induces autophagic cell death and *α*-synuclein increase in human neuroblastoma SH-SY5Y cells. *Journal of Neuroscience Research*.

[212] Kirkland R. A., Adibhatla R. M., Hatcher J. F., Franklin J. L. (2002). Loss of cardiolipin and mitochondria during programmed neuronal death: evidence of a role for lipid peroxidation and autophagy. *Neuroscience*.

[213] Lemasters J. J., Nieminen A.-L., Qian T. (1998). The mitochondrial permeability transition in cell death: a common mechanism in necrosis, apoptosis and autophagy. *Biochimica et Biophysica Acta—Bioenergetics*.

[214] Jangamreddy J. R., Los M. J. (2012). Mitoptosis, a novel mitochondrial death mechanism leading predominantly to activation of autophagy. *Hepatitis Monthly*.

[215] Lyamzaev K. G., Nepryakhina O. K., Saprunova V. B. (2008). Novel mechanism of elimination of malfunctioning mitochondria (mitoptosis): formation of mitoptotic bodies and extrusion of mitochondrial material from the cell. *Biochimica et Biophysica Acta (BBA)—Bioenergetics*.

[216] Gorman A. M., Healy S. J. M., Jäger R., Samali A. (2012). Stress management at the ER: regulators of ER stress-induced apoptosis. *Pharmacology and Therapeutics*.

[217] Doyle K. M., Kennedy D., Gorman A. M., Gupta S., Healy S. J. M., Samali A. (2011). Unfolded proteins and endoplasmic reticulum stress in neurodegenerative disorders. *Journal of Cellular and Molecular Medicine*.

[218] Szegezdi E., Logue S. E., Gorman A. M., Samali A. (2006). Mediators of endoplasmic reticulum stress-induced apoptosis. *EMBO Reports*.

[219] Ding W.-X., Ni H.-M., Gao W. (2007). Differential effects of endoplasmic reticulum stress-induced autophagy on cell survival. *The Journal of Biological Chemistry*.

[220] Nikoletopoulou V., Markaki M., Palikaras K., Tavernarakis N. (2013). Crosstalk between apoptosis, necrosis and autophagy. *Biochimica et Biophysica Acta—Molecular Cell Research*.

[221] Morishima N., Nakanishi K., Takenouchi H., Shibata T., Yasuhiko Y. (2002). An endoplasmic reticulum stress-specific caspase cascade in apoptosis. Cytochrome c-independent activation of caspase-9 by caspase-12. *The Journal of Biological Chemistry*.

[222] Ola M. S., Nawaz M., Ahsan H. (2011). Role of Bcl-2 family proteins and caspases in the regulation of apoptosis. *Molecular and Cellular Biochemistry*.

[223] O'Connor L., Strasser A., O'Reilly L. A. (1998). Bim: a novel member of the Bcl-2 family that promotes apoptosis. *The EMBO Journal*.

[224] Ott M., Gogvadze V., Orrenius S., Zhivotovsky B. (2007). Mitochondria, oxidative stress and cell death. *Apoptosis*.

[225] Ivashchenko O., Van Veldhoven P. P., Brees C., Ho Y.-S., Terlecky S. R., Fransen M. (2011). Intraperoxisomal redox balance in mammalian cells: oxidative stress and interorganellar cross-talk. *Molecular Biology of the Cell*.

[226] Wang B., Van Veldhoven P. P., Brees C. (2013). Mitochondria are targets for peroxisome-derived oxidative stress in cultured mammalian cells. *Free Radical Biology and Medicine*.

[227] Kaufmann S. H., Hengartner M. O. (2001). Programmed cell death: alive and well in the new millennium. *Trends in Cell Biology*.

[228] Zhao M., Antunes F., Eaton J. W., Brunk U. T. (2003). Lysosomal enzymes promote mitochondrial oxidant production, cytochrome c release and apoptosis. *European Journal of Biochemistry*.

[229] Antunes F., Cadenas E., Brunk U. T. (2001). Apoptosis induced by exposure to a low steady-state concentration of H_2_O_2_ is a consequence of lysosomal rupture. *Biochemical Journal*.

[230] Ogawa Y., Kobayashi T., Nishioka A. (2004). Reactive oxygen species-producing site in radiation-induced apoptosis of human peripheral T cells: involvement of lysosomal membrane destabilization. *International Journal of Molecular Medicine*.

[231] Nilsson E., Ghassemifar R., Brunk U. T. (1997). Lysosomal heterogeneity between and within cells with respect to resistance against oxidative stress. *Histochemical Journal*.

[232] Raymond M.-A., Mollica L., Vigneault N. (2003). Blockade of the apoptotic machinery by cyclosporin A redirects cell death toward necrosis in arterial endothelial cells: regulation by reactive oxygen species and cathepsin D. *The FASEB Journal*.

[233] Gollnick P. D., King D. W. (1969). Effect of exercise and training on mitochondria of rat skeletal muscle. *The American Journal of Physiology*.

[234] King D. W., Gollnick P. D. (1970). Ultrastructure of rat heart and liver after exhaustive exercise. *The American Journal of Physiology*.

[235] Ebbeling C. B., Clarkson P. M. (1989). Exercise-induced muscle damage and adaptation. *Sports Medicine*.

[236] McCutcheon L. J., Byrd S. K., Hodgson D. R. (1992). Ultrastructural changes in skeletal muscle after fatiguing exercise. *Journal of Applied Physiology*.

[237] Clarkson P. M. (1997). Eccentric exercise and muscle damage. *International Journal of Sports Medicine, Supplement*.

[238] Warburton D. E. R., Nicol C. W., Bredin S. S. D. (2006). Health benefits of physical activity: the evidence. *Canadian Medical Association Journal*.

[239] Colberg S. R. (2007). Physical activity, insulin action, and diabetes prevention and control. *Current Diabetes Reviews*.

[240] Wannamethee S. G., Shaper A. G., Walker M. (1998). Changes in physical activity, mortality, and incidence of coronary heart disease in older men. *The Lancet*.

[241] O'Gorman D. J., Krook A. (2008). Exercise and the treatment of diabetes and obesity. *Endocrinology and Metabolism Clinics of North America*.

[242] Edelmann F., Grabs V., Halle M. (2014). Exercise training in heart failure. *Internist*.

[243] Dillard C. J., Litov R. E., Savin W. M., Dumelin E. E., Tappel A. L. (1978). Effects of exercise, vitamin E, and ozone on pulmonary function and lipid peroxidation. *Journal of Applied Physiology Respiratory Environmental and Exercise Physiology*.

[244] Davies K. J. A., Quintanilha A. T., Brooks G. A., Packer L. (1982). Free radicals and tissue damage produced by exercise. *Biochemical and Biophysical Research Communications*.

[245] Kumar C. T., Reddy V. K., Prasad M., Thyagaraju K., Reddanna P. (1992). Dietary supplementation of vitamin E protects heart tissue from exercise-induced oxidant stress. *Molecular and Cellular Biochemistry*.

[246] Jackson M. J., Edwards R. H. T., Symons M. C. R. (1985). Electron spin resonance studies of intact mammalian skeletal muscle. *Biochimica et Biophysica Acta (BBA)—Molecular Cell Research*.

[247] Ashton T., Rowlands C. C., Jones E. (1998). Electron spin resonance spectroscopic detection of oxygen-centred radicals in human serum following exhaustive exercise. *European Journal of Applied Physiology and Occupational Physiology*.

[248] Mrakic-Sposta S., Gussoni M., Porcelli S. (2015). Training effects on ROS production determined by electron paramagnetic resonance in master swimmers. *Oxidative Medicine and Cellular Longevity*.

[249] Ashton T., Young I. S., Peters J. R. (1999). Electron spin resonance spectroscopy, exercise, and oxidative stress: an ascorbic acid intervention study. *Journal of Applied Physiology*.

[250] Alessio H. M., Goldfarb A. H. (1988). Lipid peroxidation and scavenger enzymes during exercise: adaptive response to training. *Journal of Applied Physiology*.

[251] Venditti P., Di Meo S. (1996). Antioxidants, tissue damage, and endurance in trained and untrained young male rats. *Archives of Biochemistry and Biophysics*.

[252] Venditti P., Di Meo S. (1997). Effect of training on antioxidant capacity, tissue damage, and endurance of adult male rats. *International Journal of Sports Medicine*.

[253] Carfagna S., Napolitano G., Barone D., Pinto G., Pollio A., Venditti P. (2015). Dietary supplementation with the microalga *Galdieria sulphuraria* (Rhodophyta) reduces prolonged exercise-induced oxidative stress in rat tissues. *Oxidative Medicine and Cellular Longevity*.

[254] Huang C.-C., Lin T.-J., Lu Y.-F., Chen C.-C., Huang C.-Y., Lin W.-T. (2009). Protective effects of L-arginine supplementation against exhaustive exercise-induced oxidative stress in young rat tissues. *Chinese Journal of Physiology*.

[255] Ziolkowski W., Flis D. J., Halon M. (2015). Prolonged swimming promotes cellular oxidative stress and p66Shc phosphorylation, but does not induce oxidative stress in mitochondria in the rat heart. *Free Radical Research*.

[256] Liu J., Yeo H. C., Övervik-Douki E. (2000). Chronically and acutely exercised rats: biomarkers of oxidative stress and endogenous antioxidants. *Journal of Applied Physiology*.

[257] Suzuki M., Katamine S., Tatsumi S. (1983). Exercise-induced enhancement of lipid peroxide metabolism in tissues and their transference into the brain in rat. *Journal of Nutritional Science and Vitaminology*.

[258] Sentürk Ü. K., Gündüz F., Kuru O. (2001). Exercise-induced oxidative stress affects erythrocytes in sedentary rats but not exercise-trained rats. *Journal of Applied Physiology*.

[259] Ramos D., Martins E. G., Viana-Gomes D., Casimiro-Lopes G., Salerno V. P. (2013). Biomarkers of oxidative stress and tissue damage released by muscle and liver after a single bout of swimming exercise. *Applied Physiology, Nutrition and Metabolism*.

[260] Asami S., Hirano T., Yamaguchi R., Tsurudome Y., Itoh H., Kasai H. (1998). Effects of forced and spontaneous exercise on 8-hydroxydeoxyguanosine levels in rat organs. *Biochemical and Biophysical Research Communications*.

[261] Lovlin R., Cottle W., Pyke I., Kavanagh M., Belcastro A. N. (1987). Are indices of free radical damage related to exercise intensity. *European Journal of Applied Physiology and Occupational Physiology*.

[262] Alessio H. M., Goldfarb A. H., Cutler R. G. (1988). MDA content increases in fast- and slow-twitch skeletal muscle with intensity of exercise in a rat. *The American Journal of Physiology*.

[263] Meister A., Anderson M. E. (1983). Glutathione. *Annual Review of Biochemistry*.

[264] Lew H., Pyke S., Quintanilha A. (1985). Changes in the glutathione status of plasma, liver and muscle following exhaustive exercise in rats. *FEBS Letters*.

[265] Sen C. K., Atalay M., Hanninen O. (1994). Exercise-induced oxidative stress: glutathione supplementation and deficiency. *Journal of Applied Physiology*.

[266] Pyke S., Lew H., Quintanilha A. (1986). Severe depletion in liver glutathione during physical exercise. *Biochemical and Biophysical Research Communications*.

[267] Lu S. C., Garcia-Ruiz C., Kuhlenkamp J., Ookhtens M., Salas-Prato M., Kaplowitz N. (1990). Hormonal regulation of glutathione efflux. *Journal of Biological Chemistry*.

[268] Galbo H. (1983). *Hormonal Adaptations to Exercise*.

[269] de Quiroga G. B. (1992). Brown fat thermogenesis and exercise: two examples of physiological oxidative stress?. *Free Radical Biology & Medicine*.

[270] Chance B., Sies H., Boveris A. (1979). Hydroperoxide metabolism in mammalian organs. *Physiological Reviews*.

[271] Green H. J., Duhamel T. A., Smith I. C. (2011). Muscle metabolic, enzymatic and transporter responses to a session of prolonged cycling. *European Journal of Applied Physiology*.

[272] Di Meo S., Venditti P. (2001). Mitochondria in exercise-induced oxidative stress. *Biological Signals and Receptors*.

[273] Venditti P., Bari A., Di Stefano L., Di Meo S. (2007). Role of mitochondria in exercise-induced oxidative stress in skeletal muscle from hyperthyroid rats. *Archives of Biochemistry and Biophysics*.

[274] Bo H., Jiang N., Ma G. (2008). Regulation of mitochondrial uncoupling respiration during exercise in rat heart: role of reactive oxygen species (ROS) and uncoupling protein 2. *Free Radical Biology and Medicine*.

[275] Sjödin B., Hellsten Westing Y., Apple F. S. (1990). Biochemical mechanisms for oxygen free radical formation during exercise. *Sports Medicine*.

[276] Brand M. D. (2000). Uncoupling to survive? The role of mitochondrial inefficiency in ageing. *Experimental Gerontology*.

[277] Fernström M., Tonkonogi M., Sahlin K. (2004). Effects of acute and chronic endurance exercise on mitochondrial uncoupling in human skeletal muscle. *The Journal of Physiology*.

[278] Nicholls D. G., Locke R. M. (1984). Thermogenic mechanisms in brown fat. *Physiological Reviews*.

[279] Zhou M., Lin B.-Z., Coughlin S., Vallega G., Pilch P. F. (2000). UCP-3 expression in skeletal muscle: effects of exercise, hypoxia, and AMP-activated protein kinase. *American Journal of Physiology—Endocrinology and Metabolism*.

[280] Ricquier D., Bouillaud F. (2000). The uncoupling protein homologues: UCP1, UCP2, UCP3, StUCP and AtUCP. *Biochemical Journal*.

[281] Talbot D. A., Lambert A. J., Brand M. D. (2004). Production of endogenous matrix superoxide from mitochondrial complex I leads to activation of uncoupling protein 3. *FEBS Letters*.

[282] Brookes P. S., Land J. M., Clark J. B., Heales S. J. R. (1998). Peroxynitrite and brain mitochondria: evidence for increased proton leak. *Journal of Neurochemistry*.

[283] Echtay K. S., Esteves T. C., Pakay J. L. (2003). A signalling role for 4-hydroxy-2-nonenal in regulation of mitochondrial uncoupling. *The EMBO Journal*.

[284] Zhang Y., Marcillat O., Giulivi C., Ernster L., Davies K. J. A. (1990). The oxidative inactivation of mitochondrial electron transport chain components and ATPase. *The Journal of Biological Chemistry*.

[285] Cassina A., Radi R. (1996). Differential inhibitory action of nitric oxide and peroxynitrite on mitochondrial electron transport. *Archives of Biochemistry and Biophysics*.

[286] Madsen K., Ertbjerg P., Djurhuus M. S., Pedersen P. K. (1996). Calcium content and respiratory control index of skeletal muscle mitochondria during exercise and recovery. *American Journal of Physiology—Endocrinology and Metabolism*.

[287] Gunter T. E., Pfeiffer D. R. (1990). Mechanisms by which mitochondria transport calcium. *American Journal of Physiology—Cell Physiology*.

[288] Vesce S., Jekabsons M. B., Johnson-Cadwell L. I., Nicholls D. G. (2005). Acute glutathione depletion restricts mitochondrial ATP export in cerebellar granule neurons. *The Journal of Biological Chemistry*.

[289] Sun Q.-A., Wang B., Miyagi M., Hess D. T., Stamler J. S. (2013). Oxygen-coupled redox regulation of the skeletal muscle ryanodine receptor/Ca^2+^ release channel (RyR1): sites and nature of oxidative modification. *The Journal of Biological Chemistry*.

[290] Hidalgo C., Sánchez G., Barrientos G., Aracena-Parks P. (2006). A transverse tubule NADPH oxidase activity stimulates calcium release from isolated triads via ryanodine receptor type 1 S-glutathionylation. *The Journal of Biological Chemistry*.

[291] Sakellariou G. K., Vasilaki A., Palomero J. (2013). Studies of mitochondrial and nonmitochondrial sources implicate nicotinamide adenine dinucleotide phosphate oxidase(*s*) in the increased skeletal muscle superoxide generation that occurs during contractile activity. *Antioxidants & Redox Signaling*.

[292] Espinosa A., Leiva A., Peña M. (2006). Myotube depolarization generates reactive oxygen species through NAD(P)H oxidase; ROS-elicited Ca^2+^ stimulates ERK, CREB, early genes. *Journal of Cellular Physiology*.

[293] Pearson T., Kabayo T., Ng R., Chamberlain J., McArdle A., Jackson M. J. (2014). Skeletal muscle contractions induce acute changes in cytosolic superoxide, but slower responses in mitochondrial superoxide and cellular hydrogen peroxide. *PLoS ONE*.

[294] Dröge W. (2002). Free radicals in the physiological control of cell function. *Physiological Reviews*.

[295] Jorquera G., Altamirano F., Contreras-Ferrat A. (2013). Cav1.1 controls frequency-dependent events regulating adult skeletal muscle plasticity. *Journal of Cell Science*.

[296] Díaz-Vegas A., Campos C. A., Contreras-Ferrat A. (2015). ROS production via P2Y_1_-PKC-NOX2 is triggered by extracellular ATP after electrical stimulation of skeletal muscle cells. *PLoS ONE*.

[297] Mortensen S. P., González-Alonso J., Nielsen J.-J., Saltin B., Hellsten Y. (2009). Muscle interstitial ATP and norepinephrine concentrations in the human leg during exercise and ATP infusion. *Journal of Applied Physiology*.

[298] Hellsten Y., Maclean D., Rådegran G., Saltin B., Bangsbo J. (1998). Adenosine concentrations in the interstitium of resting and contracting human skeletal muscle. *Circulation*.

[299] McKelvey T. G., Hollwarth M. E., Granger D. N., Engerson T. D., Landler U., Jones H. P. (1988). Mechanisms of conversion of xanthine dehydrogenase to xanthine oxidase in ischemic rat liver and kidney. *The American Journal of Physiology—Gastrointestinal and Liver Physiology*.

[300] Parks D. A., Williams T. K., Beckman J. S. (1988). Conversion of xanthine dehydrogenase to oxidase in ischemic rat intestine: a reevaluation. *American Journal of Physiology—Gastrointestinal and Liver Physiology*.

[301] Jarasch E.-D., Grund C., Bruder G., Heid H. W., Keenan T. W., Franke W. W. (1981). Localization of xanthine oxidase in mammary-gland epithelium and capillary endothelium. *Cell*.

[302] Kooij A., Schijns M., Frederiks W. M., Van Noorden C. J. F., James J. (1992). Distribution of xanthine oxidoreductase activity in human tissues: a histochemical and biochemical study. *Virchows Archiv B: Cell Pathology Including Molecular Pathology*.

[303] Felig P., Wahren J. (1971). Amino acid metabolism in exercising man. *The Journal of Clinical Investigation*.

[304] Hellsten-Westing Y., Kaijser L., Ekblom B., Sjödin B. (1994). Exchange of purines in human liver and skeletal muscle with short-term exhaustive exercise. *American Journal of Physiology*.

[305] Koyama K., Kaya M., Ishigaki T. (1999). Role of xanthine oxidase in delayed lipid peroxidation in rat liver induced by acute exhausting exercise. *European Journal of Applied Physiology and Occupational Physiology*.

[306] Gomez-Cabrera M.-C., Borrás C., Pallardo F. V., Sastre J., Ji L. L., Viña J. (2005). Decreasing xanthine oxidase-mediated oxidative stress prevents useful cellular adaptations to exercise in rats. *Journal of Physiology*.

[307] Gómez-Cabrera M.-C., Pallardó F. V., Sastre J., Viña J., Garcia-Del-Moral L. (2003). Allopurinol and markers of muscle damage among participants in the tour de France. *Journal of the American Medical Association*.

[308] Hellsten Y., Apple F. S., Sjödin B. (1996). Effect of sprint cycle training on activities of antioxidant enzymes in human skeletal muscle. *Journal of Applied Physiology*.

[309] Duarte J. A. R., Appell H.-J., Carvalho F., Bastos M. L., Soares J. M. C. (1993). Endothelium-derived oxidative stress may contribute to exercise-induced muscle damage. *International Journal of Sports Medicine*.

[310] Armstrong R. B., Warren G. L., Warren J. A. (1991). Mechanisms of exercise-induced muscle fibre injury. *Sports Medicine*.

[311] Veskoukis A. S., Nikolaidis M. G., Kyparos A. (2008). Effects of xanthine oxidase inhibition on oxidative stress and swimming performance in rats. *Applied Physiology, Nutrition and Metabolism*.

[312] Das D. K., Engelman R. M., Clement R., Otani H., Prasad M. R., Rao P. S. (1987). Role of xanthine oxidase inhibitor as free radical scavenger: a novel mechanism of action of allopurinol and oxypurinol in myocardial salvage. *Biochemical and Biophysical Research Communications*.

[313] Lindsay S., Liu T.-H., Xu J. (1991). Role of xanthine dehydrogenase and oxidase in focal cerebral ischemic injury to rat. *The American Journal of Physiology—Heart and Circulatory Physiology*.

[314] Novelli G. P., Bracciotti G., Falsini S. (1990). Spin-trappers and vitamin E prolong endurance to muscle fatigue in mice. *Free Radical Biology and Medicine*.

[315] Wadley G. D., Nicolas M. A., Hiam D. S., McConell G. K. (2013). Xanthine oxidase inhibition attenuates skeletal muscle signaling following acute exercise but does not impair mitochondrial adaptations to endurance training. *American Journal of Physiology—Endocrinology and Metabolism*.

[316] Venditti P., Napolitano G., Barone D., Di Meo S. (2014). Effect of training and vitamin e administration on rat liver oxidative metabolism. *Free Radical Research*.

[317] Venditti P., Napolitano G., Barone D., Di Meo S. (2014). Vitamin E supplementation modifies adaptive responses to training in rat skeletal muscle. *Free Radical Research*.

[318] Venditti P., Napolitano G., Barone D., Pervito E., Di Meo S. (2016). Vitamin E-enriched diet reduces adaptive responses to training determining respiratory capacity and redox homeostasis in rat heart. *Free Radical Research*.

[319] Venditti P., Bari A., Di Stefano L., Agnisola C., Di Meo S. (2008). Effect of T3 treatment on the response to ischemia-reperfusion of heart preparations from sedentary and trained rats. *Pflugers Archiv European Journal of Physiology*.

[320] Powers S. K., Ji L. L., Leeuwenburgh C. (1999). Exercise training-induced alterations in skeletal muscle antioxidant capacity: a brief review. *Medicine and Science in Sports and Exercise*.

[321] Atalay M., Sen C. K. (1999). Physical exercise and antioxidant defenses in the heart. *Annals of the New York Academy of Sciences*.

[322] Gore M., Fiebig R., Hollander J., Leeuwenburgh C., Ohno H., Ji L. L. (1998). Endurance training alters antioxidant enzyme gene expression in rat skeletal muscle. *Canadian Journal of Physiology and Pharmacology*.

[323] Hollander J., Fiebig R., Gore M. (1999). Superoxide dismutase gene expression in skeletal muscle: fiber-specific adaptation to endurance training. *American Journal of Physiology—Regulatory Integrative and Comparative Physiology*.

[324] Venditti P., Masullo P., Di Meo S. (1999). Effect of training on H_2_O_2_ release by mitochondria from rat skeletal muscle. *Archives of Biochemistry and Biophysics*.

[325] Starnes J. W., Barnes B. D., Olsen M. E. (2007). Exercise training decreases rat heart mitochondria free radical generation but does not prevent Ca^2+^-induced dysfunction. *Journal of Applied Physiology*.

[326] Servais S., Couturier K., Koubi H. (2003). Effect of voluntary exercise on H_2_O_2_ release by subsarcolemmal and intermyofibrillar mitochondria. *Free Radical Biology and Medicine*.

[327] Boss O., Samec S., Desplanches D. (1998). Effect of endurance training on mRNA expression of uncoupling proteins 1, 2, and 3 in the rat. *The FASEB Journal*.

[328] Lee Y., Kwak H.-B., Hord J., Kim J.-H., Lawler J. M. (2015). Exercise training attenuates age-dependent elevation of angiotensin II type 1 receptor and Nox2 signaling in the rat heart. *Experimental Gerontology*.

[329] Cocks M., Shaw C. S., Shepherd S. O. (2016). Sprint interval and moderate-intensity continuous training have equal benefits on aerobic capacity, insulin sensitivity, muscle capillarisation and endothelial eNOS/NAD(P)Hoxidase protein ratio in obese men. *Journal of Physiology*.

[330] Cocks M., Shaw C. S., Shepherd S. O. (2013). Sprint interval and endurance training are equally effective in increasing muscle microvascular density and eNOS content in sedentary males. *Journal of Physiology*.

[331] Touati S., Montezano A. C., Meziri F., Riva C., Touyz R. M., Laurant P. (2015). Exercise training protects against atherosclerotic risk factors through vascular NADPH oxidase, extracellular signal-regulated kinase 1/2 and stress-activated protein kinase/c-Jun N-terminal kinase downregulation in obese rats. *Clinical and Experimental Pharmacology and Physiology*.

[332] Baldwin K. M., Klinkerfuss G. H., Terjung R. L., Molé P. A., Holloszy J. O. (1972). Respiratory capacity of white, red, and intermediate muscle: adaptative response to exercise. *The American Journal of Physiology*.

[333] Davies K. J. A., Packer L., Brooks G. A. (1981). Biochemical adaptation of mitochondria, muscle, and whole-animal respiration to endurance training. *Archives of Biochemistry and Biophysics*.

[334] Wasserman D. H., Cherrington A. D. (1991). Hepatic fuel metabolism during muscular work: role and regulation. *American Journal of Physiology—Endocrinology and Metabolism*.

[335] Scarpulla R. C. (2002). Nuclear activators and coactivators in mammalian mitochondrial biogenesis. *Biochimica et Biophysica Acta (BBA)—Gene Structure and Expression*.

[336] Liang H., Ward W. F. (2006). PGC-1*α*: a key regulator of energy metabolism. *Advances in Physiology Education*.

[337] Irrcher I., Adhihetty P. J., Sheehan T., Joseph A.-M., Hood D. A. (2003). PPAR*γ* coactivator-1*α* expression during thyroid hormone- and contractile activity-induced mitochondrial adaptations. *American Journal of Physiology—Cell Physiology*.

[338] Baar K., Wende A. R., Jones T. E. (2002). Adaptations of skeletal muscle to exercise: rapid increase in the transcriptional coactivator PGC-1. *The FASEB Journal*.

[339] Wang L., Mascher H., Psilander N., Blomstrand E., Sahlin K. (2011). Resistance exercise enhances the molecular signaling of mitochondrial biogenesis induced by endurance exercise in human skeletal muscle. *Journal of Applied Physiology*.

[340] Haase T. N., Ringholm S., Leick L. (2011). Role of PGC-1*α* in exercise and fasting-induced adaptations in mouse liver. *The American Journal of Physiology—Regulatory Integrative and Comparative Physiology*.

[341] Kavazis A. N., Smuder A. J., Powers S. K. (2014). Effects of short-term endurance exercise training on acute doxorubicin-induced FoxO transcription in cardiac and skeletal muscle. *Journal of Applied Physiology*.

[342] Kang C., Ji L. L. (2012). Role of PGC-1*α* signaling in skeletal muscle health and disease. *Annals of the New York Academy of Sciences*.

[343] Olesen J., Kiilerich K., Pilegaard H. (2010). PGC-1*α*-mediated adaptations in skeletal muscle. *Pflugers Archiv European Journal of Physiology*.

[344] Kong X., Wang R., Xue Y. (2010). Sirtuin 3, a new target of PGC-1*α*, plays an important role in the suppression of ROS and mitochondrial biogenesis. *PLoS ONE*.

[345] Shi T., Wang F., Stieren E., Tong Q. (2005). SIRT3, a mitochondrial sirtuin deacetylase, regulates mitochondrial function and thermogenesis in brown adipocytes. *Journal of Biological Chemistry*.

[346] St-Pierre J., Lin J., Krauss S. (2003). Bioenergetic analysis of peroxisome proliferator-activated receptor *γ* coactivators 1*α* and 1*β* (PGC-1*α* and PGC-1*β*) in muscle cells. *The Journal of Biological Chemistry*.

[347] Silveira L. R., Pilegaard H., Kusuhara K., Curi R., Hellsten Y. (2006). The effect of reactive oxygen species and antioxidants on basal and contraction-induced gene expression of PGC-1*α*, UCP3 and HKII in primary rat skeletal muscle cells. *Biochemical and Biophysical Acta*.

[348] St-Pierre J., Drori S., Uldry M. (2006). Suppression of reactive oxygen species and neurodegeneration by the PGC-1 transcriptional coactivators. *Cell*.

[349] Higashida K., Kim S. H., Higuchi M., Holloszy J. O., Han D.-H. (2011). Normal adaptations to exercise despite protection against oxidative stress. *American Journal of Physiology—Endocrinology and Metabolism*.

[350] Gomez-Cabrera M.-C., Domenech E., Romagnoli M. (2008). Oral administration of vitamin C decreases muscle mitochondrial biogenesis and hampers training-induced adaptations in endurance performance. *American Journal of Clinical Nutrition*.

[351] Ristow M., Zarsea K., Oberbach A. (2009). Antioxidants prevent health-promoting effects of physical exercise in humans. *Proceedings of the National Academy of Sciences of the United States of America*.

[352] Paulsen G., Cumming K. T., Holden G. (2014). Vitamin C and E supplementation hampers cellular adaptation to endurance training in humans: a double-blind, randomised, controlled trial. *The Journal of Physiology*.

[353] Popkin B. M. (2006). Global nutrition dynamics: the world is shifting rapidly toward a diet linked with noncommunicable diseases. *The American Journal of Clinical Nutrition*.

[354] Rocha M., Apostolova N., Herance J. R., Rovira-Llopis S., Hernandez-Mijares A., Victor V. M. (2014). Perspectives and potential applications of mitochondria-targeted antioxidants in cardiometabolic diseases and type 2 diabetes. *Medicinal Research Reviews*.

[355] Apostolova N., Victor V. M. (2015). Molecular strategies for targeting antioxidants to mitochondria: therapeutic implications. *Antioxidants & Redox Signaling*.

[356] Brehm A., Krssak M., Schmid A. I., Nowotny P., Waldhäusl W., Roden M. (2006). Increased lipid availability impairs insulin-stimulated ATP synthesis in human skeletal muscle. *Diabetes*.

[357] Ritz P., Berrut G. (2005). Mitochondrial function, energy expenditure, aging and insulin resistance. *Diabetes and Metabolism*.

[358] Frisard M., Ravussin E. (2006). Energy metabolism and oxidative stress: impact on the metabolic syndrome and the aging process. *Endocrine*.

[359] Hernandez-Mijares A., Rocha M., Apostolova N. (2011). Mitochondrial complex I impairment in leukocytes from type 2 diabetic patients. *Free Radical Biology & Medicine*.

[360] Brownlee M. (2005). The pathobiology of diabetic complications: a unifying mechanism. *Diabetes*.

[361] Ashrafian H., Frenneaux M. P., Opie L. H. (2007). Metabolic mechanisms in heart failure. *Circulation*.

[362] Nishikawa T., Edelstein D., Du X. L. (2000). Normalizing mitochondrial superoxide production blocks three pathways of hyperglycaemic damage. *Nature*.

[363] Victor V. M., Rocha M., Herance R., Hernandez-Mijares A. (2011). Oxidative stress and mitochondrial dysfunction in type 2 diabetes. *Current Pharmaceutical Design*.

[364] Tatsch E., Carvalho J. A. M. D., Hausen B. S. (2015). Oxidative DNA damage is associated with inflammatory response, insulin resistance and microvascular complications in type 2 diabetes. *Mutation Research/Fundamental and Molecular Mechanisms of Mutagenesis*.

[365] Padmalayam I. (2012). Targeting mitochondrial oxidative stress through lipoic acid synthase: a novel strategy to manage diabetic cardiovascular disease. *Cardiovascular and Hematological Agents in Medicinal Chemistry*.

[366] Faid I., Al-Hussaini H., Kilarkaje N. (2015). Resveratrol alleviates diabetes-induced testicular dysfunction by inhibiting oxidative stress and c-Jun N-terminal kinase signaling in rats. *Toxicology and Applied Pharmacology*.

[367] Verkaart S., Koopman W. J. H., Cheek J. (2007). Mitochondrial and cytosolic thiol redox state are not detectably altered in isolated human NADH:ubiquinone oxidoreductase deficiency. *Biochimica et Biophysica Acta (BBA)—Molecular Basis of Disease*.

[368] Murphy M. P. (2014). Antioxidants as therapies: can we improve on nature?. *Free Radical Biology and Medicine*.

[369] Rovira-Llopis S., Rocha M., Falcon R. (2013). Is myeloperoxidase a key component in the ROS-induced vascular damage related to nephropathy in type 2 diabetes?. *Antioxidants & Redox Signaling*.

[370] Rovira-Llopis S., Diáz-Morales N., Bañuls C. (2015). Is autophagy altered in the leukocytes of type 2 diabetic patients?. *Antioxidants & Redox Signaling*.

[371] Rovira-Llopis S., Bañuls C., Apostolova N. (2014). Is glycemic control modulating endoplasmic reticulum stress in leukocytes of type 2 diabetic patients?. *Antioxidants and Redox Signaling*.

[372] Victor V. M., Rocha M., Bañuls C. (2011). Induction of oxidative stress and human leukocyte/endothelial cell interactions in polycystic ovary syndrome patients with insulin resistance. *Journal of Clinical Endocrinology and Metabolism*.

[373] Persson M. F., Franzén S., Catrina S.-B. (2012). Coenzyme Q10 prevents GDP-sensitive mitochondrial uncoupling, glomerular hyperfiltration and proteinuria in kidneys from db/db mice as a model of type 2 diabetes. *Diabetologia*.

[374] Hernandez-Mijares A., Rocha M., Rovira-Llopis S. (2013). Human leukocyte/endothelial cell interactions and mitochondrial dysfunction in type 2 diabetic patients and their association with silent myocardial ischemia. *Diabetes Care*.

[375] Green K., Brand M. D., Murphy M. P. (2004). Prevention of mitochondrial oxidative damage as a therapeutic strategy in diabetes. *Diabetes*.

[376] Chacko B. K., Reily C., Srivastava A. (2010). Prevention of diabetic nephropathy in Ins2^+/−AkitaJ^ mice by the mitochondria-targeted therapy MitoQ. *The Biochemical Journal*.

[377] Morino K., Petersen K. F., Shulman G. I. (2006). Molecular mechanisms of insulin resistance in humans and their potential links with mitochondrial dysfunction. *Diabetes*.

[378] Björnheden T., Babyi A., Bondjers G., Wiklund O. (1996). Accumulation of lipoprotein fractions and subfractions in the arterial wall, determined in an in vitro perfusion system. *Atherosclerosis*.

[379] Campos H., Arnold K. S., Balestra M. E., Innerarity T. L., Krauss R. M. (1996). Differences in receptor binding of LDL subfractions. *Arteriosclerosis, Thrombosis, and Vascular Biology*.

[380] Galeano N. F., Milne R., Marcel Y. L. (1994). Apoprotein B structure and receptor recognition of triglyceride-rich low density lipoprotein (LDL) is modified in small LDL but not in triglyceride-rich LDL of normal size. *The Journal of Biological Chemistry*.

[381] Tribble D. L., Holl L. G., Wood P. D., Krauss R. M. (1992). Variations in oxidative susceptibility among six low density lipoprotein subfractions of differing density and particle size. *Atherosclerosis*.

[382] Nishikawa T., Araki E. (2007). Impact of mitochondrial ROS production in the pathogenesis of diabetes mellitus and its complications. *Antioxidants & Redox Signaling*.

[383] Khodaeian M., Tabatabaei-Malazy O., Qorbani M., Farzadfar F., Amini P., Larijani B. (2015). Effect of vitamins C and E on insulin resistance in diabetes: A Meta-Analysis Study. *European Journal of Clinical Investigation*.

[384] Petersen K. F., Dufour S., Befroy D., Garcia R., Shulman G. I. (2004). Impaired mitochondrial activity in the insulin-resistant offspring of patients with type 2 diabetes. *The New England Journal of Medicine*.

[385] Oseid S., Beck Nielsen H., Pedersen O., Sovik O. (1977). Decreased binding of insulin to its receptor in patients with congenital generalized lipodystrophy. *New England Journal of Medicine*.

[386] Banerjee R. R., Rangwala S. M., Shapiro J. S. (2004). Regulation of fasted blood glucose by resistin. *Science*.

[387] Yamauchi T., Kamon J., Waki H. (2001). The fat-derived hormone adiponectin reverses insulin resistance associated with both lipoatrophy and obesity. *Nature Medicine*.

[388] Bogacka I., Xie H., Bray G. A., Smith S. R. (2005). Pioglitazone induces mitochondrial biogenesis in human subcutaneous adipose tissue in vivo. *Diabetes*.

[389] Parish R., Petersen K. F. (2005). Mitochondrial dysfunction and type 2 diabetes. *Current Diabetes Reports*.

[390] Kostis J. B., Sanders M. (2005). The association of heart failure with insulin resistance and the development of type 2 diabetes. *American Journal of Hypertension*.

[391] Ferrannini E., Cushman W. C. (2012). Diabetes and hypertension: the bad companions. *The Lancet*.

[392] Katz R., Budoff M. J., O'Brien K. D., Wong N. D., Nasir K. (2016). The metabolic syndrome and diabetes mellitus as predictors of thoracic aortic calcification as detected by non-contrast computed tomography in the Multi-Ethnic Study of Atherosclerosis. *Diabetic Medicine*.

[393] McGavock J. M., Lingvay I., Zib I. (2007). Cardiac steatosis in diabetes mellitus: a 1H-magnetic resonance spectroscopy study. *Circulation*.

[394] Whaley-Connell A., Govindarajan G., Habibi J. (2007). Angiotensin II-mediated oxidative stress promotes myocardial tissue remodeling in the transgenic (mRen2) 27 Ren2 rat. *The American Journal of Physiology—ndocrinology and Metabolism*.

[395] Katakam P. V. G., Jordan J. E., Snipes J. A., Tulbert C. D., Miller A. W., Busija D. W. (2007). Myocardial preconditioning against ischemia-reperfusion injury is abolished in Zucker obese rats with insulin resistance. *American Journal of Physiology—Regulatory Integrative and Comparative Physiology*.

[396] Kumar B., Kowluru A., Kowluru R. A. (2015). Lipotoxicity augments glucotoxicity-induced mitochondrial damage in the development of diabetic retinopathy. *Investigative Ophthalmology & Visual Science*.

[397] Nishio Y., Kanazawa A., Nagai Y., Inagaki H., Kashiwagi A. (2004). Regulation and role of the mitochondrial transcription factor in the diabetic rat heart. *Annals of the New York Academy of Sciences*.

[398] Suematsu N., Tsutsui H., Wen J. (2003). Oxidative stress mediates tumor necrosis factor-*α*-induced mitochondrial DNA damage and dysfunction in cardiac myocytes. *Circulation*.

[399] Finck B. N., Kelly D. P. (2007). Peroxisome proliferator-activated receptor *γ* coactivator-1 (PGC-1) regulatory cascade in cardiac physiology and disease. *Circulation*.

[400] Kim J.-A., Montagnani M., Kwang K. K., Quon M. J. (2006). Reciprocal relationships between insulin resistance and endothelial dysfunction: molecular and pathophysiological mechanisms. *Circulation*.

[401] Davidson S. M., Duchen M. R. (2007). Endothelial mitochondria: contributing to vascular function and disease. *Circulation Research*.

[402] Zheng Z., Chen H., Ke G. (2009). Protective effect of perindopril on diabetic retinopathy is associated with decreased vascular endothelial growth factor-to-pigment epithelium-derived factor ratio: involvement of a mitochondria-reactive oxygen species pathway. *Diabetes*.

[403] Montagnani M., Chen H., Barr V. A., Quon M. J. (2001). Insulin-stimulated activation of eNOS is independent of Ca^2+^ but requires phosphorylation by Akt at Ser^1179^. *The Journal of Biological Chemistry*.

[404] Duplain H., Burcelin Ŕ., Sartori C. (2001). Insulin resistance, hyperlipidemia, and hypertension in mice lacking endothelial nitric oxide synthase. *Circulation*.

[405] Maechler P., Wollheim C. B. (2001). Mitochondrial function in normal and diabetic *β*-cells. *Nature*.

[406] Han J., Bae J. H., Kim S.-Y. (2004). Taurine increases glucose sensitivity of UCP2-overexpressing *β*-cells by ameliorating mitochondrial metabolism. *American Journal of Physiology—Endocrinology and Metabolism*.

[407] Soejima A., Inoue K., Takai D. (1996). Mitochondrial DNA is required for regulation of glucose-stimulated insulin secretion in a mouse pancreatic beta cell line, MIN6. *The Journal of Biological Chemistry*.

[408] Devasagayam T. P. A., Tilak J. C., Boloor K. K., Sane K. S., Ghaskadbi S. S., Lele R. D. (2004). Free radicals and antioxidants in human health: current status and future prospects. *The Journal of Association of Physicians of India*.

[409] Fang Y.-Z., Yang S., Wu G. (2002). Free radicals, antioxidants, and nutrition. *Nutrition*.

[410] Lander H. M. (1997). An essential role for free radicals and derived species in signal transduction. *FASEB Journal*.

[411] Villeda S. A., Plambeck K. E., Middeldorp J. (2014). Young blood reverses age-related impairments in cognitive function and synaptic plasticity in mice. *Nature Medicine*.

[412] Kaushik D. K., Basu A. (2013). A friend in need may not be a friend indeed: role of microglia in neurodegenerative diseases. *CNS & Neurological Disorders—Drug Targets*.

[413] Saha R. N., Pahan K. (2006). Regulation of inducible nitric oxide synthase gene in glial cells. *Antioxidants and Redox Signaling*.

[414] Aguzzi A., Barres B. A., Bennett M. L. (2013). Microglia: scapegoat, saboteur, or something else?. *Science*.

[415] Adler A. S., Kawahara T. L. A., Segal E., Chang H. Y. (2008). Reversal of aging by NF*κ*B blockade. *Cell Cycle*.

[416] Chauhan A. K., Mittra N., Kumar V., Patel D. K., Singh C. (2015). Inflammation and B-cell lymphoma-2 associated X protein regulate zinc-induced apoptotic degeneration of rat nigrostriatal dopaminergic neurons. *Molecular Neurobiology*.

[417] Sierra A., Gottfried-Blackmore A. C., Mcewen B. S., Bulloch K. (2007). Microglia derived from aging mice exhibit an altered inflammatory profile. *Glia*.

[418] Brandes R. P., Weissmann N., Schröder K. (2014). Nox family NADPH oxidases: molecular mechanisms of activation. *Free Radical Biology and Medicine*.

[419] Saud K., Herrera-Molina R., Bernhardi R. (2005). Pro- and anti-inflammatory cytokines regulate the ERK pathway: implication of the timing for the activation of microglial cells. *Neurotoxicity Research*.

[420] Tesseur I., Wyss-Coray T. (2006). A role for TGF-*β* signaling in neurodegeneration: evidence from genetically engineered models. *Current Alzheimer Research*.

[421] Fellner L., Jellinger K. A., Wenning G. K., Stefanova N. (2011). Glial dysfunction in the pathogenesis of *α*-synucleinopathies: emerging concepts. *Acta Neuropathologica*.

[422] Halliwell B. (2006). Oxidative stress and neurodegeneration: where are we now?. *Journal of Neurochemistry*.

[423] Aruoma O. I., Kaur H., Halliwell B. (1991). Oxygen free radicals and human diseases. *Journal of the Royal Society of Health*.

[424] Brown L. A. S., Harris F. L., Jones D. P. (1997). Ascorbate deficiency and oxidative stress in the alveolar type II cell. *American Journal of Physiology—Lung Cellular and Molecular Physiology*.

[425] Miquel J., Economos A. C., Fleming J., Johnson J. E. (1980). Mitochondrial role in cell aging. *Experimental Gerontology*.

[426] Sastre J., Pallardó F. V., de la Asunción J. G., Viña J. (2000). Mitochondria, oxidative stress and aging. *Free Radical Research*.

[427] Thomas C. E., Aust S. D. (1986). Free radicals and environmental toxins. *Annals of Emergency Medicine*.

[428] Niki E. (2000). Free radicals in the 1900's: from in vitro to in vivo. *Free Radical Research*.

[429] Schempp H., Albrecht-Goepfert E., Elstner E. F. (1999). Detection of the production of reactive oxygen species by neutrophils in whole blood: modulation by adamantanes and triggering by Fe^3+^-ions. *Zeitschrift für Naturforschung*.

[430] van der B. S., Veen M. P., Heeringa P. (2009). Myeloperoxidase: molecular mechanisms of action and their relevance to human health and disease. *Antioxidants & Redox Signaling*.

[431] Whiteman M., Spencer J. P. E., Jenner A., Halliwell B. (1999). Hypochlorous acid-induced DNA base modification: potentiation by nitrite: biomarkers of DNA damage by reactive oxygen species. *Biochemical and Biophysical Research Communications*.

[432] Saran M., Beck-Speier I., Fellerhoff B., Bauer G. (1999). Phagocytic killing of microorganisms by radical processes: consequences of the reaction of hydroxyl radicals with chloride yielding chlorine atoms. *Free Radical Biology and Medicine*.

[433] Eto K., Asada T., Arima K., Makifuchi T., Kimura H. (2002). Brain hydrogen sulfide is severely decreased in Alzheimer's disease. *Biochemical and Biophysical Research Communications*.

[434] Bergendi L., Beneš L., Ďuracková Z., Ferenčik M. (1999). Chemistry, physiology and pathology of free radicals. *Life Sciences*.

[435] Schulz J. B., Matthews R. T., Beal M. F. (1995). Role of nitric oxide in neurodegenerative diseases. *Current Opinion in Neurology*.

[436] Hara M. R., Cascio M. B., Sawa A. (2006). GAPDH as a sensor of NO stress. *Biochimica et Biophysica Acta (BBA)—Molecular Basis of Disease*.

[437] Castrillo A., Pennington D. J., Otto F., Parker P. J., Owen M. J., Boscá L. (2001). Protein kinase C*ε* is required for macrophage activation and defense against bacterial infection. *The Journal of Experimental Medicine*.

[438] Fagni L., Bockaert J. (1996). Effects of nitric oxide on glutamate-gated channels and other ionic channels. *Journal of Chemical Neuroanatomy*.

[439] Persinger R. L., Poynter M. E., Ckless K., Janssen-Heininger Y. M. W. (2002). Molecular mechanisms of nitrogen dioxide induced epithelial injury in the lung. *Molecular and Cellular Biochemistry*.

[440] Moldéus P. (1993). Toxicity induced by nitrogen dioxide in experimental animals and isolated cell systems. *Scandinavian Journal of Work, Environment & Health*.

[441] Sagai M., Ichinose T., Kubota K. (1984). Studies on the biochemical effects of nitrogen dioxide. IV. Relation between the change of lipid peroxidation and the antioxidative protective system in rat lungs upon life span exposure to low levels of NO_2_. *Toxicology and Applied Pharmacology*.

[442] Byun J., Mueller D. M., Fabjan J. S., Heinecke J. W. (1999). Nitrogen dioxide radical generated by the myeloperoxidase-hydrogen peroxide-nitrite system promotes lipid peroxidation of low density lipoprotein. *FEBS Letters*.

[443] Podrez E. A., Schmitt D., Hoff H. F., Hazen S. L. (1999). Myeloperoxidase-generated reactive nitrogen species convert LDL into an atherogenic form in vitro. *The Journal of Clinical Investigation*.

[444] Wang J. H., Duddle J., Devalia J. L., Davies R. J. (1995). Nitrogen dioxide increases eosinophil activation in the early-phase response to nasal allergen provocation. *International Archives of Allergy and Immunology*.

[445] Dedon P. C., Tannenbaum S. R. (2004). Reactive nitrogen species in the chemical biology of inflammation. *Archives of Biochemistry and Biophysics*.

[446] Zhang R., Brennan M.-L., Shen Z. (2002). Myeloperoxidase functions as a major enzymatic catalyst for initiation of lipid peroxidation at sites of inflammation. *The Journal of Biological Chemistry*.

[447] Chow C. K., Tappel A. L. (1974). Response of glutathione peroxidase to dietary selenium in rats. *The Journal of Nutrition*.

[448] Smith P. J., Tappel A. L., Chow C. K. (1974). Glutathione peroxidase activity as a function of dietary selenomethionine. *Nature*.

[449] Beal M. F. (1995). Aging, energy, and oxidative stress in neurodegenerative diseases. *Annals of Neurology*.

[450] Sharp A. H., Ross C. A. (1996). Neurobiology of Huntington's disease. *Neurobiology of Disease*.

[451] Bowling A. C., Beal M. F. (1995). Bioenergetic and oxidative stress in neurodegenerative diseases. *Life Sciences*.

[452] Ferrante R. J., Browne S. E., Shinobu L. A. (1997). Evidence of increased oxidative damage in both sporadic and familial amyotrophic lateral sclerosis. *Journal of Neurochemistry*.

[453] Butterfield D. A., Drake J., Pocernich C., Castegna A. (2001). Evidence of oxidative damage in Alzheimer's disease brain: central role for amyloid *β*-peptide. *Trends in Molecular Medicine*.

[454] Zhu X., Lee H.-G., Casadesus G. (2005). Oxidative imbalance in Alzheimer's disease. *Molecular Neurobiology*.

[455] Good P. F., Werner P., Hsu A., Olanow C. W., Perl D. P. (1996). Evidence of neuronal oxidative damage in Alzheimer's disease. *The American Journal of Pathology*.

[456] Nelson O., Tu H., Lei T., Bentahir M., De Strooper B., Bezprozvanny I. (2007). Familial Alzheimer disease-linked mutations specifically disrupt Ca^2+^ leak function of presenilin 1. *The Journal of Clinical Investigation*.

[457] Murrell J. R., Hake A. M., Quaid K. A., Farlow M. R., Ghetti B. (2000). Early-onset Alzheimer disease caused by a new mutation (V717L) in the amyloid precursor protein gene. *Archives of Neurology*.

[458] Ji Y., Gong Y., Gan W., Beach T., Holtzman D. M., Wisniewski T. (2003). Apolipoprotein E isoform-specific regulation of dendritic spine morphology in apolipoprotein E transgenic mice and Alzheimer's disease patients. *Neuroscience*.

[459] Baxter L. C., Caselli R. J., Johnson S. C., Reiman E., Osborne D. (2003). Apolipoprotein E *ε*4 affects new learning in cognitively normal individuals at risk for Alzheimer's disease. *Neurobiology of Aging*.

[460] Wilhelmus M. M. M., Otte-Höller I., Davis J., Van Nostrand W. E., De Waal R. M. W., Verbeek M. M. (2005). Apolipoprotein E genotype regulates amyloid-*β* cytotoxicity. *The Journal of Neuroscience*.

[461] Ye S., Huang Y., Müllendorff K. Apolipoprotein (apo) E4 enhances amyloid beta peptide production in cultured neuronal cells: apoE structure as a potential therapeutic target.

[462] Lauderback C. M., Kanski J., Hackett J. M., Maeda N., Kindy M. S., Butterfield D. A. (2002). Apolipoprotein E modulates Alzheimer's A*β*(1-42)-induced oxidative damage to synaptosomes in an allele-specific manner. *Brain Research*.

[463] Ramassamy C., Averill D., Beffert U. (2000). Oxidative insults are associated with apolipoprotein E genotype in Alzheimer's disease brain. *Neurobiology of Disease*.

[464] Blennow K., de Leon M. J., Zetterberg H. (2006). Alzheimer's disease. *The Lancet*.

[465] Jellinger K. A. (2004). Traumatic brain injury as a risk factor for Alzheimer's disease. *Journal of Neurology, Neurosurgery and Psychiatry*.

[466] Jellinger K. A., Paulus W., Wrocklage C., Litvan I. (2001). Traumatic brain injury as a risk factor for Alzheimer disease. Comparison of two retrospective autopsy cohorts with evaluation of ApoE genotype. *BMC Neurology*.

[467] Mortimer J. A., Borenstein A. R., Gosche K. M., Snowdon D. A. (2005). Very early detection of Alzheimer neuropathology and the role of brain reserve in modifying its clinical expression. *Journal of Geriatric Psychiatry and Neurology*.

[468] Mortimer J. A., Van Duijn C. M., Chandra V. (1991). Head trauma as a risk factor for Alzheimer's disease: a collaborative re-analysis of case-control studies. *International Journal of Epidemiology*.

[469] Turner P. R., O'Connor K., Tate W. P., Abraham W. C. (2003). Roles of amyloid precursor protein and its fragments in regulating neural activity, plasticity and memory. *Progress in Neurobiology*.

[470] Glenner G. G., Wong C. W. (1984). Alzheimer's disease and Down's syndrome: sharing of a unique cerebrovascular amyloid fibril protein. *Biochemical and Biophysical Research Communications*.

[471] Glenner G. G., Wong C. W., Quaranta V., Eanes E. D. (1984). The amyloid deposits in Alzheimer's disease: their nature and pathogenesis. *Applied Pathology*.

[472] Boyd-Kimball D., Castegna A., Sultana R. (2005). Proteomic identification of proteins oxidized by A*β*(1–42) in synaptosomes: implications for Alzheimer's disease. *Brain Research*.

[473] Boyd-Kimball D., Sultana R., Fai Poon H. (2005). Proteomic identification of proteins specifically oxidized by intracerebral injection of amyloid *β*-peptide (1–42) into rat brain: implications for Alzheimer's disease. *Neuroscience*.

[474] Butterfield D. A., Boyd-Kimball D. (2004). Amyloid *β*-peptide(1-42) contributes to the oxidative stress and neurodegeneration found in Alzheimer disease brain. *Brain Pathology*.

[475] Dewachter I., Van Dorpe J., Smeijers L. (2000). Aging increased amyloid peptide and caused amyloid plaques in brain of old APP/V717I transgenic mice by a different mechanism than mutant presenilin1. *Journal of Neuroscience*.

[476] Kumar-Singh S., Cras P., Wang R. (2002). Dense-core senile plaques in the Flemish variant of Alzheimer's disease are vasocentric. *The American Journal of Pathology*.

[477] Pasalar P., Najmabadi H., Noorian A. R. (2002). An Iranian family with Alzheimer's disease caused by a novel APP mutation (THr714ALa). *Neurology*.

[478] Tsubuki S., Takaki Y., Saido T. C. (2003). Dutch, Flemish, Italian, and Arctic mutations of APP and resistance of A*β* to physiologically relevant proteolytic degradation. *The Lancet*.

[479] Hsiao K. (1998). Transgenic mice expressing Alzheimer amyloid precursor proteins. *Experimental Gerontology*.

[480] Westerman M. A., Cooper-Blacketer D., Mariash A. (2002). The relationship between A*β* and memory in the Tg2576 mouse model of Alzheimer's disease. *The Journal of Neuroscience*.

[481] Jankowsky J. L., Fadale D. J., Anderson J. (2004). Mutant presenilins specifically elevate the levels of the 42 residue *β*-amyloid peptide in vivo: evidence for augmentation of a 42-specific *γ* secretase. *Human Molecular Genetics*.

[482] Knight W. D., Kennedy J., Mead S. (2007). A novel presenilin 1 deletion (p.L166del) associated with early onset familial Alzheimer's disease. *European Journal of Neurology*.

[483] Leissring M. A., Parker I., LaFerla F. M. (1999). Presenilin-2 mutations modulate amplitude and kinetics of inositol 1,4,5-trisphosphate-mediated calcium signals. *Journal of Biological Chemistry*.

[484] Shrimpton A. E., Schelper R. L., Linke R. P. (2007). A presenilin 1 mutation (L420R) in a family with early onset Alzheimer disease, seizures and cotton wool plaques, but not spastic paraparesis. *Neuropathology*.

[485] Almkvist O., Axelman K., Basun H. (2003). Clinical findings in nondemented mutation carriers predisposed to Alzheimer's disease: a model of mild cognitive impairment. *Acta Neurologica Scandinavica. Supplementum*.

[486] Zekanowski C., Styczyńska M., Pepłońska B. (2003). Mutations in presenilin 1, presenilin 2 and amyloid precursor protein genes in patients with early-onset Alzheimer's disease in Poland. *Experimental Neurology*.

[487] Grigorenko A. P., Rogaev E. I. (2007). Molecular basics of Alzheimer's disease. *Molekuliarnaia Biologiia*.

[488] Calabrese V., Scapagnini G., Colombrita C. (2003). Redox regulation of heat shock protein expression in aging and neurodegenerative disorders associated with oxidative stress: a nutritional approach. *Amino Acids*.

[489] Mohmmad Abdul H., Sultana R., Keller J. N., St Clair D. K., Markesbery W. R., Butterfield D. A. (2006). Mutations in amyloid precursor protein and presenilin-1 genes increase the basal oxidative stress in murine neuronal cells and lead to increased sensitivity to oxidative stress mediated by amyloid *β*-peptide (1–42), H_2_O_2_ and kainic acid: implications for Alzheimer's disease. *Journal of Neurochemistry*.

[490] Resende R., Moreira P. I., Proença T. (2008). Brain oxidative stress in a triple-transgenic mouse model of Alzheimer disease. *Free Radical Biology & Medicine*.

[491] Bertram L., Blacker D., Mullin K. (2000). Evidence for genetic linkage of Alzheimer's disease to chromosome 10q. *Science*.

[492] Fabian R. H., Perez-Polo J. R., Kent T. A. (2000). Electrochemical monitoring of superoxide anion production and cerebral blood flow: effect of interleukin-1*β* pretreatment in a model of focal ischemia and reperfusion. *Journal of Neuroscience Research*.

[493] Qiu W. Q., Walsh D. M., Ye Z. (1998). Insulin-degrading enzyme regulates extracellular levels of amyloid *β*-protein by degradation. *The Journal of Biological Chemistry*.

[494] Cook D. G., Leverenz J. B., McMillan P. J. (2003). Reduced hippocampal insulin-degrading enzyme in late-onset Alzheimer's disease is associated with the apolipoprotein E-*ε*4 allele. *The American Journal of Pathology*.

[495] Kim Y. O., Kim H.-J., Kim G. S. (2009). Panax ginseng protects against global ischemia injury in rat hippocampus. *Journal of Medicinal Food*.

[496] Son S. M., Kang S., Choi H., Mook-Jung I. (2015). Statins induce insulin-degrading enzyme secretion from astrocytes via an autophagy-based unconventional secretory pathway. *Molecular Neurodegeneration*.

[497] Craft S., Asthana S., Schellenberg G. (1999). Insulin metabolism in Alzheimer's disease differs according to apolipoprotein E genotype and gender. *Neuroendocrinology*.

[498] Craft S. (2007). Insulin resistance and Alzheimer's disease pathogenesis: potential mechanisms and implications for treatment. *Current Alzheimer Research*.

[499] Ertekin-Taner N., Graff-Radford N., Younkin L. H. (2000). Linkage of plasma A*β*42 to a quantitative locus on chromosome 10 in late-onset Alzheimer's disease pedigrees. *Science*.

[500] Van Uden E., Sagara Y., Van Uden J. (2000). A protective role of the low density lipoprotein receptor-related protein against amyloid *β*-protein toxicity. *Journal of Biological Chemistry*.

[501] Moir R. D., Tanzi R. E. (2005). LRP-mediated clearance of A*β* is inhibited by KPI-containing isoforms of APP. *Current Alzheimer Research*.

[502] Du Y., Bales K. R., Dodel R. C. (1998). *α*2-Macroglobulin attenuates *β*-amyloid peptide 1-40 fibril formation and associated neurotoxicity of cultured fetal rat cortical neurons. *Journal of Neurochemistry*.

[503] Hughes S. R., Khorkova O., Goyal S. (1998). *α*
_2_-macroglobulin associates with *β*-amyloid peptide and prevents fibril formation. *Proceedings of the National Academy of Sciences of the United States of America*.

[504] Dodel R. C., Du Y., Bales K. R. (2000). *α*2 Macroglobulin and the risk of Alzheimer's disease. *Neurology*.

[505] Harry G. J., Lefebvre d'Hellencourt C., Bruccoleri A., Schmechel D. (2000). Age-dependent cytokine responses: trimethyltin hippocampal injury in wild-type, APOE knockout, and APOE4 mice. *Brain, Behavior, and Immunity*.

[506] Chakraborty S., Kaushik D. K., Gupta M., Basu A. (2010). Inflammasome signaling at the heart of central nervous system pathology. *Journal of Neuroscience Research*.

[507] Qin L., Liu Y., Cooper C., Liu B., Wilson B., Hong J.-S. (2002). Microglia enhance *β*-amyloid peptide-induced toxicity in cortical and mesencephalic neurons by producing reactive oxygen species. *Journal of Neurochemistry*.

[508] Chinta S. J., Mallajosyula J. K., Rane A., Andersen J. K. (2010). Mitochondrial alpha-synuclein accumulation impairs complex I function in dopaminergic neurons and results in increased mitophagy in vivo. *Neuroscience Letters*.

[509] Bonini N. M., Giasson B. I. (2005). Snaring the function of *α*-synuclein. *Cell*.

[510] Pisanu A., Lecca D., Mulas G. (2014). Dynamic changes in pro-and anti-inflammatory cytokines in microglia after PPAR-*γ* agonist neuroprotective treatment in the MPTPp mouse model of progressive Parkinson's disease. *Neurobiology of Disease*.

[511] Alam Z. I., Jenner A., Daniel S. E. (1997). Oxidative DNA damage in the parkinsonian brain: an apparent selective increase in S-hydroxyguanine levels in substanzia nigra. *Journal of Neurochemistry*.

[512] Alam Z. I., Daniel S. E., Lees A. J., Marsden D. C., Jenner P., Halliwell B. (1997). A generalised increase in protein carbonyls in the brain in Parkinson's but not incidental Lewy body disease. *Journal of Neurochemistry*.

[513] Harischandra D. S., Jin H., Anantharam V., Kanthasamy A., Kanthasamy A. G. (2015). *α*-Synuclein protects against manganese neurotoxic insult during the early stages of exposure in a dopaminergic cell model of Parkinson's disease. *Toxicological Sciences*.

[514] Giasson B. I., Duda J. E., Murray I. V. J. (2000). Oxidative damage linked to neurodegeneration by selective *α*-synuclein nitration in synucleinopathy lesions. *Science*.

[515] Tiwari M., Lopez-Cruzan M., Morgan W. W., Herman B. (2011). Loss of caspase-2-dependent apoptosis induces autophagy after mitochondrial oxidative stress in primary cultures of young adult cortical neurons. *The Journal of Biological Chemistry*.

[516] Langston J. W., Ballard P., Tetrud J. W., Irwin I. (1983). Chronic parkinsonism in humans due to a product of meperidine-analog synthesis. *Science*.

[517] Jackson-Lewis V., Blesa J., Przedborski S. (2012). Animal models of Parkinson's disease. *Parkinsonism and Related Disorders*.

[518] Abdelsalam R. M., Safar M. M. (2015). Neuroprotective effects of vildagliptin in rat rotenone Parkinson's disease model: role of RAGE-NF*κ*B and Nrf2-antioxidant signaling pathways. *Journal of Neurochemistry*.

[519] Chiu C.-C., Yeh T.-H., Lai S.-C. (2015). Neuroprotective effects of aldehyde dehydrogenase 2 activation in rotenone-induced cellular and animal models of parkinsonism. *Experimental Neurology*.

[520] Eom S. A., Kim D. W., Shin M. J. (2015). Protective effects of PEP-1-Catalase on stress-induced cellular toxicity and MPTP-induced Parkinson's disease. *BMB Reports*.

[521] Liu L., Peritore C., Ginsberg J., Shih J., Arun S., Donmez G. (2015). Protective role of SIRT5 against motor deficit and dopaminergic degeneration in MPTP-induced mice model of Parkinson's disease. *Behavioural Brain Research*.

[522] Qin J., Wu M., Yu S. (2015). Pyrroloquinoline quinone-conferred neuroprotection in rotenone models of Parkinson's disease. *Toxicology Letters*.

[523] Wang S., He H., Chen L., Zhang W., Zhang X., Chen J. (2015). Protective effects of salidroside in the MPTP/MPP^+^-induced model of Parkinson's disease through ROS-NO-related mitochondrion pathway. *Molecular Neurobiology*.

[524] Bové J., Prou D., Perier C., Przedborski S. (2005). Toxin-induced models of Parkinson's disease. *NeuroRx*.

[525] Kostrzewa J. P., Kostrzewa R. A., Kostrzewa R. M., Brus R., Nowak P. (2015). Perinatal 6-hydroxydopamine to produce a lifelong model of severe Parkinson's disease. *Current Topics in Behavioral Neurosciences*.

[526] Wang Y.-H., Xuan Z.-H., Tian S., Du G.-H. (2015). Echinacoside protects against 6-hydroxydopamine-induced mitochondrial dysfunction and inflammatory responses in PC12 cells via reducing ROS production. *Evidence-Based Complementary and Alternative Medicine*.

[527] Zhu G., Chen G., Shi L. (2014). PEGylated rhFGF-2 conveys long-term neuroprotection and improves neuronal function in a rat model of Parkinson’s disease. *Molecular Neurobiology*.

[528] Pan X., Chen C., Huang J., Wei H., Fan Q. (2015). Neuroprotective effect of combined therapy with hyperbaric oxygen and madopar on 6-hydroxydopamine-induced Parkinson's disease in rats. *Neuroscience Letters*.

[529] Giuliani P., Ballerini P., Buccella S. (2015). Guanosine protects glial cells against 6-hydroxydopamine toxicity. *Advances in Experimental Medicine and Biology*.

[530] Afshin-Majd S., Khalili M., Roghani M., Mehranmehr N., Baluchnejadmojarad T. (2015). Carnosine exerts neuroprotective effect against 6-hydroxydopamine toxicity in hemiparkinsonian rat. *Molecular Neurobiology*.

[531] Kaplitt M. G., Feigin A., Tang C. (2007). Safety and tolerability of gene therapy with an adeno-associated virus (AAV) borne GAD gene for Parkinson's disease: an open label, phase I trial. *The Lancet*.

[532] LeWitt P. A., Rezai A. R., Leehey M. A. (2011). AAV2-GAD gene therapy for advanced Parkinson's disease: a double-blind, sham-surgery controlled, randomised trial. *The Lancet Neurology*.

[533] Cleveland D. W., Rothstein J. D. (2001). From Charcot to Lou Gehrig: deciphering selective motor neuron death in ALS. *Nature Reviews Neuroscience*.

[534] Nirmalananthan N., Greensmith L. (2005). Amyotrophic lateral sclerosis: recent advances and future therapies. *Current Opinion in Neurology*.

[535] Mitsumoto H., Santella R., Liu X. (2008). Oxidative stress biomarkers in sporadic ALS. *Amyotrophic Lateral Sclerosis*.

[536] Bowling A. C., Schulz J. B., Brown R. H., Beal M. F. (1993). Superoxide dismutase activity, oxidative damage, and mitochondrial energy metabolism in familial and sporadic amyotrophic lateral sclerosis. *Journal of Neurochemistry*.

[537] Moruno Manchon J. F., Uzor N.-E., Dabaghian Y., Furr-Stimming E. E., Finkbeiner S., Tsvetkov A. S. (2015). Cytoplasmic sphingosine-1-phosphate pathway modulates neuronal autophagy. *Scientific Reports*.

[538] Rubinsztein D. C. (2006). The roles of intracellular protein-degradation pathways in neurodegeneration. *Nature*.

[539] Wong E., Cuervo A. M. (2010). Autophagy gone awry in neurodegenerative diseases. *Nature Neuroscience*.

[540] Rojas F., Gonzalez D., Cortes N. (2015). Reactive oxygen species trigger motoneuron death in non-cell-autonomous models of als through activation of c-Abl signaling. *Frontiers in Cellular Neuroscience*.

[541] Chen Y., Liu H., Guan Y. (2015). The altered autophagy mediated by TFEB in animal and cell models of amyotrophic lateral sclerosis. *American Journal of Translational Research*.

[542] Hwang J.-Y., Min S.-W., Jeon Y.-T. (2015). Effect of coenzyme Q_10_ on spinal cord ischemia-reperfusion injury. *Journal of Neurosurgery: Spine*.

[543] Jing L., He M.-T., Chang Y. (2015). Coenzyme Q10 protects astrocytes from ROS-Induced damage through inhibition of Mitochondria-Mediated cell death pathway. *International Journal of Biological Sciences*.

[544] Moreira P. I., Zhu X., Wang X. (2010). Mitochondria: a therapeutic target in neurodegeneration. *Biochimica et Biophysica Acta—Molecular Basis of Disease*.

[545] Qi L., Sun X., Li F.-E. (2015). Hmgb1 promotes mitochondrial dysfunction-triggered striatal neurodegeneration via autophagy and apoptosis activation. *PLoS ONE*.

